# Use of Live Interactive Webcasting for an International Postgraduate Module in eHealth: Case Study Evaluation

**DOI:** 10.2196/jmir.1225

**Published:** 2009-11-13

**Authors:** Ray B Jones, Inocencio Maramba, Maged N Kamel Boulos, Tara Alexander

**Affiliations:** ^1^Faculty of HealthUniversity of PlymouthPlymouthUK

**Keywords:** Webcasting, synchronous e-learning, eHealth

## Abstract

**Background:**

Producing “traditional” e-learning can be time consuming, and in a topic such as eHealth, it may have a short shelf-life. Students sometimes report feeling isolated and lacking in motivation. Synchronous methods can play an important part in any blended approach to learning.

**Objective:**

The aim was to develop, deliver, and evaluate an international postgraduate module in eHealth using live interactive webcasting.

**Methods:**

We developed a hybrid solution for live interactive webcasting using a scan converter, mixer, and digitizer, and video server to embed a presenter-controlled talking head or copy of the presenter’s computer screen (normally a PowerPoint slide) in a student chat room. We recruited 16 students from six countries and ran weekly 2.5-hour live sessions for 10 weeks. The content included the use of computers by patients, patient access to records, different forms of e-learning for patients and professionals, research methods in eHealth, geographic information systems, and telehealth. All sessions were recorded—presentations as video files and the student interaction as text files. Students were sent an email questionnaire of mostly open questions seeking their views of this form of learning. Responses were collated and anonymized by a colleague who was not part of the teaching team.

**Results:**

Sessions were generally very interactive, with most students participating actively in breakout or full-class discussions. In a typical 2.5-hour session, students posted about 50 messages each. Two students did not complete all sessions; one withdrew from the pressure of work after session 6, and one from illness after session 7. Fourteen of the 16 responded to the feedback questionnaire. Most students (12/14) found the module useful or very useful, and all would recommend the module to others. All liked the method of delivery, in particular the interactivity, the variety of students, and the “closeness” of the group. Most (11/14) felt “connected” with the other students on the course. Many students (11/14) had previous experience with asynchronous e-learning, two as teachers; 12/14 students suggested advantages of synchronous methods, mostly associated with the interaction and feedback from teachers and peers.

**Conclusions:**

This model of synchronous e-learning based on interactive live webcasting was a successful method of delivering an international postgraduate module. Students found it engaging over a 10-week course. Although this is a small study, given that synchronous methods such as interactive webcasting are a much easier transition for lecturers used to face-to-face teaching than are asynchronous methods, they should be considered as part of the blend of e-learning methods. Further research and development is needed on interfaces and methods that are robust and accessible, on the most appropriate blend of synchronous and asynchronous work for different student groups, and on learning outcomes and effectiveness.

## Introduction

To date, most e-learning has tended to be asynchronous through Internet access to websites and other interactive materials, which are used by students in their own time. With the development of new technologies, however, there is potential for the development of interactive, participatory, synchronous methods of e-learning [1]. Webcasting is one such method that also offers students the ability to participate in real-time discussion with each other and with the presenting lecturer, from any Internet-connected computer that plays sound.

DiMaria-Ghalili and Ostrow were among the first to use webcasting routinely. They changed from interactive TV to webcasting in the spring of 2003 to deliver distance learning for graduate nurses in rural West Virginia, USA [2,3]. Although some of their students were still using dial-up connections to the Internet, the method was acceptable and thought to be more interactive than interactive TV. A number of centers have used or experimented with either live streaming [4] or filmed lectures [5], but these have generally not included any synchronous interactivity. Others have used Web conferencing with video connection from all participants [6], which is more suitable for peer-to-peer meetings than for student education. Webcasting has been used nationally in continuing education in pathology [7] and nursing [8] but sometimes fails to become routine practice [9]. Some found problems with webcasting because of the lack of interpersonal interaction [10]. However, Reynolds et al reported successful trials of webcasting in dental undergraduate and postgraduate education [11]; for example, they found that students preferred webcasting to traditional lectures because of active and nonthreatening participation. We developed interactive webcasting that combined a live video stream with chat room interactivity [12] and had used this extensively for open “webinars” and occasional lectures, but not for a complete module.

The aim of the present study was to develop, deliver, and evaluate an international postgraduate module in eHealth using live interactive webcasting.

## Methods

### Participants

We advertised the module (cost £220) on the University of Plymouth website and via various email discussion lists. Eleven paying students applied and were accepted to the module. In addition, we invited five (three full time, two part time) of our “distance” PhD students who had an interest in eHealth. Students came from a wide range of backgrounds and posts: academics in health or medicine (2), health service public health (1), diagnostic imaging technologist (1), clinical governance (1), health visiting (ie, home nursing) (1), private sector health informatics (2), complementary cancer care information department (1), computing science student (1), head of hospital IT department (1), librarian (2), journalist (1) working in NHS (National Health Service in England), and health services research (2). The 16 students came from six countries: Malaysia (1), Mauritius (1), Saudi Arabia (1), England (10), St Vincent and Grenadines (1), and Canada (2). There were 11 male and five female students, with an age range of about 24-50 years.

### Module

We ran 2.5-hour live sessions (UK time 2:00-4:30 pm) weekly for 10 weeks from October to December 2008. Although the module was available as part of a masters program, on this first occasion, all students took the module as a “stand-alone” continuing professional development. The content included the use of computers by patients, patient access to records, different forms of e-learning for patients and professionals, research methods in eHealth, geographic information systems, and telehealth. One session was a student-defined session in which students, either singly or in groups, prepared their own presentations. These were given via the video window with discussion, as usual, through the chat room.

### Webcasting System

**Figure 1:**
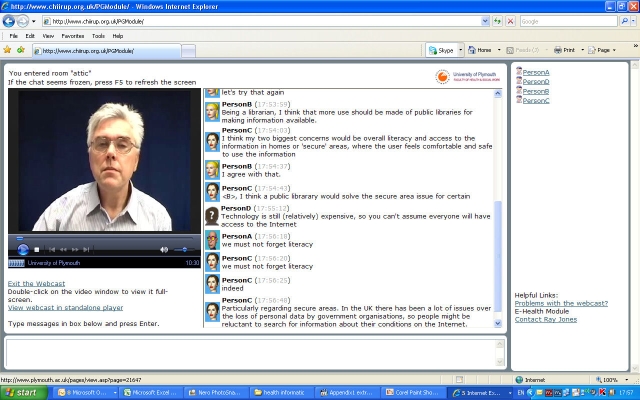
An anonymized mockup of a webcast (cartoon heads replace real photos of participants, and “PersonA” to “PersonD” replace real names)

Coming from a background of using interactive satellite TV [13], we developed a hybrid solution for live interactive webcasting using a scan converter that converted the PC signal (PowerPoint) to an analogue signal, where it was mixed by the presenter with the camera signal. The combined signal was digitized and sent via a video server to a Web page where it was embedded in a student “chat room” (Figure 1) developed using open source software. We used this webcasting system with access via a module portal. Table 1 shows the main features of this approach. All sessions were recorded: presentations were recorded as video files (see Multimedia Appendix 1) and student interaction was recorded as text files (see Multimedia Appendix 2). Handouts prior to sessions and recordings after sessions were posted as blogs on the module portal. The possibility existed for students to post their own blogs to the portal.

**Table 1:** Features of our webcasting

Live Interactive Webcasting Feature	Comment
Webcast audience is online only	We do not stream live lectures since we believe that the online audience will feel excluded. Unless the presenter concentrates fully on the distance audience, they are unlikely to achieve suitable interaction.
Live quality video showing talking head of presenter	By using a video server and good quality cameras, we achieve high-quality video and sound (rather than the familiar poor-quality video from low-end webcams). This was important for delivery but is a trade-off with the need to deliver from a mini-studio and the introduction of a 30-second delay for the video signal to reach the students (while typed chat remains instantaneous).
Live PowerPoint or presentation display	The presenter could fade between the talking head and PowerPoint using a desktop joystick.
List of people participating can be seen by presenter and participants	It is important for the presenter and participants to know who else is there and, if in groups, the composition of the groups.
Photo of participants can be seen by other participants	A photo of each student (avatar in the terminology of this software) was shown against their comments.
Participants can comment in real time by typing in chat room (text chat)	Participants did not use audio or video input. See results for student views on this design aspect.
Participants can be divided into breakout rooms for discussion	Participants can create a chat room on the fly simply by changing rooms. We typically used three or four rooms (attic, cellar, hall, kitchen), dividing participants by their birthday month or student name.
Recording of talking head, PowerPoint presentation, and participant discussion	Recordings of video, presentation, and chat room transcripts were made available on the module portal.

### Evaluation

One week after the module was completed, students were sent an email questionnaire (see Multimedia Appendix 3) that included a mix of closed and open questions seeking their views on this form of learning. The email questionnaire was divided into six sections:

A. Overall (five open and one closed question)

B. Method of delivery: with subsections on breakout groups, pace, screen layout, audio vs text, robustness and technical difficulty, connectivity with other students, downloads, portal, video window, presentation style, overall delivery method (13 open and three closed questions)

C. Content (three open questions)

D. Assessment (five open and one closed question)

E. Future and other possibilities (three open questions)

F. Marketing and promotion (four open questions)

Several reminders were sent over the next few weeks. The data generated by the survey were placed into a chart for each individual by a colleague (TA) who was not part of the teaching team (RJ, MKB, IM).

For closed questions and open questions that generated a limited range of answers, responses were noted and counted. For example, for question A3 (“Would you recommend the module to others amongst your colleagues? If so which job functions or roles?”), all respondents noted groups to whom they would recommend the module, so we concluded that all said yes. We classified their answers into five groups (see Multimedia Appendix 4).

For open questions that generated a narrative response, we used generic qualitative analysis [13,14]. Using an iterative process in order to generate themes, starting with the first question, comments were clustered under the corresponding question and then read through for themes (see Multimedia Appendix 4). Where there were multiple themes, items were tallied and the quotation that best represented the theme was selected. If a participant’s response seemed unclear, the researcher went back to the participant’s own data set to verify and clarify the information. In order to check for researcher bias, the researcher looked for conflicting data and noted those quotations. Unusual comments not related to a theme were not incorporated but were retained for team discussion. As one example, question A6 was about overall value for money and referred to the possible use of more asynchronous materials. This prompted some to comment on synchronous vs asynchronous methods, which we asked a specific question about in B2, so some A6 comments were therefore combined with others from B2. Triangulation was used in order to confirm the results. TA reviewed the anonymized results with the teaching team to compare with feedback received from participants over the course of the semester. The final data were then shared with participants to verify that their information was correctly represented.

## Results

### Response Rate to Email Questionnaire

The majority of students (14/16) responded to the request for feedback, most of them in some depth. The average length of email response was 1619 words (range 1144-2244 words), of which the questions comprised 955 words. That fact that students were prepared to respond to this degree suggests that a good degree of “connection” with the course and teaching team had been built over the 10 weeks.

### Overall Satisfaction With the Course

There were 12/14 students who found the module useful or very useful, and two who were undecided or had no opinion. However, all 14 would recommend the module to others among their colleagues. All liked the method of delivery and would be interested in taking other modules using this same method (Table 2). Some students mentioned features that they particularly liked: the variety of other students’ backgrounds (n = 3), the interactivity (n = 7), and the “closeness” of the group (n = 7).

**Table 2:** Sample quotations from participants

Aspect of Course		Quotation
Overall satisfaction with the course		“It made it easy to fit the course in around my day job.”
		“I very much liked the live webcasting as it is interactive and facilitates discussion and debates among the participants.”
Value for money and group size		“The size of the group matters. If it was large, then some members taking part would be silent. This could possibly be overcome by breaking the group into smaller discussion groups and sticking with the same breakout groups across all sessions.”
**Connectivity with other students**		
	Positive	“Surprisingly felt more connected than I thought I would, I think for two main reasons: - [I had] the ability to associate thoughts and questions through the text chats with specific individuals (this would be lost if audio interaction was adopted instead) - The group activities split us up into smaller groups, which were more manageable and interactive. I felt connected and respected. The pace was set by the presenters—they greeted everyone as if they were equally important and welcomed.”
	Negative	“There were times in the chat rooms when I felt very isolated; the others were chatting and my comment/query was missed in the exchange. Sometimes this led me to think that my input was not valid/valued.”
	Suggestions for change	“Would it be possible to have information about people closer at hand (ie, when someone has commented, it’s hard to remember who they are, where they are from, and what they do)?”
**Connection and physical environment of participation**		
	Positive (for at home)	“I was able to concentrate better, had the liberty to move around (to take water or go to toilet) and to eat without disturbing the cohorts; if I didn’t understand any concept and if I was not convinced by explanation by the tutors, I had the chance to surf the net for clarifications.”
	Negative (for at work)	“Sometimes not having somewhere to be ‘physically’ made it more difficult to take time out of everyday work to attend. I’d be sitting at my computer with headphones on but still very much ‘at work’ (ie, people in my office chatting and occasionally talking to me)—I think in a classroom situation it is perhaps easier to focus. A note to all participants to buy themselves a good pair of earphones if they are planning to listen whilst at work (ie, if they have a shared office) would be very helpful.”
	Use of more asynchronous materials such as recorded webcasts from the year before	“I think [the] risk of asynchronous [materials is that the experience] becomes less engaging—like watching a TV program rather than discussing thoughts [and] ideas with students in real time.”
		“I like the fact that the course uses real-time interaction. The real-time, two-way communication provided by the chat room is very important to the course delivery.”
	Interface	“I discovered that you could send ‘a secret message’—this was excellent, and I have had excellent 1:1 conversations with some of my colleagues.”
		“This [session where we used a slide share] worked quite well, although it reduced the space available for the chat window. If you can find a way of accommodating all three things, that’d be ideal, but on balance I think a large chat area is more important than providing access to the slides from within the Web application.”
	Audio vs text input	“Would have liked to have [the] ability to talk sometimes (although not very often, surprisingly).”
		“The advantage of text is that we could all speak simultaneously; text was also instantaneous. Bearing in mind that some participants were at the other side of the world, I am sure that there would be problems with audio feeds. Text works. We are all, increasingly, becoming used to typing/texting, and it is a comfortable medium. However, English may not be the first language for all.”
		“I do like audio, but then you need a ‘hand raising’ tool too, like in e-class.”

### Value for Money and Group Size

All but one student thought that the course was worth the &pound;220 fee; one students that the current fee level and number of students was not economic for the university and asked for their views about increasing the cost to &pound;400; opinion was divided, and one student suggested smaller incremental increases to test the level of fee. When asked about increasing the class size, some students (n = 6) said no to larger groups, one noting that it might inhibit contribution. Another suggested that this could be overcome by the use of breakout groups (see Table 2).

### Connectivity With Other Students

Most students (n = 11) felt connected with the other students in the course. One suggested improving connectivity by using the same combination of students in the breakout rooms more often. There was, however, room for improvement in the facilitation of chat rooms and in providing other prompts and information to enable connection between participants (see Table 2.)

### Volume of Chat

Multimedia Appendix 2 gives an anonymized extract of a chat transcript. To illustrate the volume of chats, we will use session 5 as a typical session. A total of 14/16 students took some part in the discussion (two were unavailable). One student had Internet connection problems and only posted three messages and then took no further part. In total, there were 756 comments posted, including 108 by two members of the teaching team (RJ, IM). The 13 students who participated in the whole session posted an average of 48 messages (range 14-124) during the 2.5-hour session. 

### Connection and Physical Environment of Participation

During the course of the module, some students had hardware connection problems; in particular, two or three connecting from NHS sites often found it more convenient to go home for the session. A few students mentioned bandwidth-related problems in the questionnaire. Home appeared the best place to study, with no interruptions from work colleagues and the ability to “be comfortable” (see Table 2).

### Use of More Asynchronous Materials Such as Recorded Webcasts From the Year Before

Students were asked if re-use of recorded materials from one year to the next would affect the course. Some students commented that use of more asynchronous material would be fine as long as there was still some sort of group facilitation and follow-up discussion, but others did not want asynchronous materials.

### Synchronous Compared to Asynchronous Methods

Some students had previous experience of asynchronous e-learning (11/14), two as teachers. Most students (12/14) suggested advantages of synchronous methods, mostly associated with the interaction and feedback from teachers and peers. Although the disadvantage of the time zone differences was noted, nine students thought that the timing of the module (2:00-4:30 pm UK time) was convenient and three students, not so convenient. One noted issues around unreliability of the synchronous technology. Another suggested that with asynchronous methods more in-depth answers can be given, but some noted that asynchronous was more impersonal and less engaging.

The advantage of interaction with other students and the lecturers was supported by comments about individual sessions. Two or three sessions that gave the students less opportunity to interact were reviewed less favourably and suggestions were made to change them: “They could have been broken into two sessions each. I didn’t get enough time to reflect on the teaching and hence was not able to contribute anything for the discussions.”

When asked about breakout groups, students thought the balance was about right, apart from the two to three sessions in which they thought that there were too few opportunities for discussion. Various comments suggested that it was important for the presenter to facilitate breakout group discussions. One student said, “I guess that this is challenging for the teacher—knowing how long to allocate for the session and being flexible enough/savvy [enough] to facilitate longer sessions where discussion is animated and cut short those where the level of discussion is clearly non-existent!”

Another student noted the problem of multiple streams of thought in chat rooms. We addressed this by using breakout rooms, but the presenters’ decisions of when to use breakout rooms, how many, and which participants to have in each room were critical in making this work.

### Improving Communications and the Interface

Although most students thought that there was a lot of interaction and that this was the best part of the course, useful comments were made on how the course could be further improved, such as through one-to-one chat. The importance of the interactivity and space available for chatting were always rated highly (see Table 2).

One student wondered if the interface could be changed on the presenter’s side so that occurrences of presenter error, such as talking while the sound was turned off, could be reduced.

Students were asked if they would prefer audio to text input. More preferred text, the advantage being that all participants can contribute simultaneously and that there may be problems with audio feeds. But still, some would like audio (see Table 2).

One student noted a specific problem with the chat room: “When attempting to scroll up to find something someone previously wrote, [the] cursor would immediately jump back down to the bottom as soon as someone [wrote] something new (so [it was] difficult to scroll through [the] history).” Two commented on the copyright-free music that we used when students were working on their own or perhaps in groups. One said, “I liked the music”; another stated, “The music was ghastly but very necessary as it was an easy way of knowing that the link was active.”

Students generally thought that the way we used the video window was acceptable. Comments included the following: “The fading back and forth was very effective when used. It not only gave a bit of a feeling of ‘interactivity’ but also broke up the slideshow nicely (great for people with shorter attention spans and a million thoughts a minute like myself!)” Another student said, “Nice to see [a] real face now and then rather than disembodied slides/voice.” But having the PowerPoint slides to download was also seen as useful: “I liked the simplicity of the slides onscreen, but having the presentation available as well meant that the complicated ones weren’t lost as they could be studied after or on printout or in a different window. Our choice.”

### Module Portal

All but one student used the portal to download materials. Nine downloaded both papers (extra reading) and the presentations; four downloaded the papers only. The portal was never used by students to post items. Comments suggested that it was not easy to use for that purpose or had not been sufficiently explained; one student said, “I would have liked to be able to share resources with other students, eg, useful websites found following the sessions.” Another said, “I still haven’t worked out how to use the blog.”

### Module Content

Students were asked about adding or subtracting from the module content. Various suggestions were made as to additional content: two suggested including eHealth education, two, behavioral theories, and three, patient information systems. If a session had to be dropped, six students would have cut session 4 (on 3D virtual worlds), but three students thought this to be the most relevant session. Votes for other sessions were spread fairly evenly, but some commented that they would not want to see the number of sessions reduced. However, to achieve more interactivity in all sessions, we will probably need to reduce the density of topics by dropping one.

### Assessment

Only five of the 16 students took the assessment, the others opting for “attendance only.” All were invited to comment on the amount of assessment (a 3-hour exam in the last of the 10 weeks and one coursework essay to be handed in 6 weeks later). Four students thought that the assessment was about right, three thought it was too heavy, and the others did not comment. Six thought that the timing was okay, and three thought the exam should be later. No one felt that there should be any concerns about the open book and distance nature of the exam.

### Marketing

Six students heard about the course from individual emails, two found it on a Google search, four heard about it from email distribution lists (two from Patient Information Forum [PIF] Aware), one from consumer-health informatics list on jiscmail, one from Afro-nets, one from NHS Connecting for Health “Health Informatics” eSpace community, and one from the Plymouth University website. Students recommended a number of sites where the module could be advertised.

## Discussion

The use of e-learning in the health professions is expanding rapidly [16]. Traditionally, e-learning has been asynchronous, but the development of learning objects can be very time consuming and not within the skill set of many academic staff. Various initiatives are underway to develop shared e-learning resources (eg, [17]), but there is a growing body of research exploring how student-student or staff-student communication (either synchronous or asynchronous) can be used in addition to, or instead of, fixed learning objects [18,19]. For example, Baecker et al have developed methods (ePresence [20,21]) that support real-time video and voice and video conferencing for a few participants, while streaming an event to many others. Methods such as these seem particularly relevant in situations such as eHealth where the academic content changes rapidly and investment in learning objects has a short shelf-life.

We have tested a novel method for delivery of a postgraduate module. As such, our evaluation was limited to a first-level study [22] in which we assessed the feasibility and the reaction of our participants. Although we cannot draw any solid conclusions from just one case study with 16 participants, we found that the use of interactive live webcasting was successful for a 10-week international course in eHealth.

This was the first time we have run this module, and the methods of delivery are novel. By definition, therefore, our participants were early adopters and so may not be typical of later recruits. Nevertheless, students from Malaysia through to mid-Canada, from a variety of backgrounds, were able to participate in a 2.5-hour online session once a week. Live presentations in which the presenter was seen and heard in a (good quality) video window fading between a talking head and PowerPoint slide worked well. Participants particularly found the discussions (by typed chat) in smaller breakout groups an important and successful element of the delivery. This agrees with our own studies with nursing and midwifery students [15,16] who knew each other from face-to-face teaching and with Reynolds et al’s trials with dental students [11]. Most of our students had experience with asynchronous e-learning and found the interactivity of this synchronous method engaging.

There were, of course, some teething problems with our webcasting, with probably the most important being limited bandwidth in NHS sites. Others who have used webcasting have had problems with connection speed, bandwidth, and server access [8,10]. Others have pointed to the need for students and staff to receive training in how the technology works [6,8]. We agree that there is a need for some staff training, but with an emphasis on how to use the technology effectively for teaching and learning rather than on how the technology works per se.

Of the 16 students who started the module, only two dropped out: one missed the last four sessions because of NHS workload, and one missed the last two sessions because of illness. Participating from home offered other advantages for some students, by having fewer interruptions or distractions.

Students participated in this synchronous international module at times ranging from 9-12 pm (Malaysia), through 2-5 pm (United Kingdom), and 7-10 am (Alberta, Canada). Most students said that they found the timing convenient, but clearly this is a selected group who chose to take the course. Given sufficient students worldwide wishing to participate in a synchronous module, it should be possible to run it at different times of the day to suit different time zones and lifestyles, although participation from Australia and New Zealand in a European module is unlikely.

E-learning based on webcasting, such as we have used, is also a much easier transition for lecturers used to face-to-face teaching, and it allows on-the-fly content tailoring guided by audience needs as one would do in a conventional lecture. Given the positive responses of our students over a 10-week course, it would appear a useful way forward. More traditional asynchronous e-learning, of course, should form part of a blend of methods. Within the structure of our webcast sessions, we were able to ask students to spend some time looking at some reusable learning objects or previously recorded webcasts. We hope also to re-use some of the recorded webcasts in subsequent years and that creating a bank of learning materials from the live webcasts will produce a useful learning resource. The research questions then center on the most cost-effective blend of synchronous and asynchronous methods.

We used an in-house developed system [12], combining TV methods with open source software. The equipment for our webcasting mini-studio cost about £8000 2 years ago. Costs of alternative hardware may now cost less, but this approach requires that the presenter be located in a studio. There are now a variety of solutions (Table 3) on offer that may be able to deliver the same or better functionality, including webcasting from any desktop. Our own university is currently trialing MS Communicator, while our attached medical school is trialing Wimba.

**Table 3:** Examples of current interactive webcasting and Web conferencing solutions for e-learning

Webcasting or Web Conferencing Solution	URL
ePresence	http://epresence.tv/
Elluminate	http://www.elluminate.com/
Dimdim	http://www.dimdim.com/
Yugma	https://www.yugma.com/
Openmeetings	http://code.google.com/p/openmeetings/
Vyew	http://vyew.com/
iVocalize	https://www.ivocalize.com/
Qwaq	http://www.qwaq.com/
Adobe Connect	http://www.adobe.com/products/acrobatconnect/
Wimba	http://www.wimba.com/products/wimba_collaboration_suite/
MS Communicator	http://office.microsoft.com/en-us/communicator/FX101729051033.aspx
Mogulus ProCaster	http://www.procaster.com/video

There are various research questions related to the use of webcasting that remain:

1. What proportion of potential learners are able to participate in webcasting? For example, we had some problems with students accessing from UK health service sites, which apparently had low bandwidth availability. Are there other methods that can overcome this, or is the solution to find alternative learning sites?

2. What is the best blend of synchronous and asynchronous methods in different learning situations? Further work is needed to explore the cost-effectiveness of different proportions of live or asynchronous contact vs individual learning, and how this varies by learner groups or environments.

3. How do these synchronous methods compare with asynchronous methods, for example, through the use of recorded videos and an asynchronous discussion? Is the quality of participant discussion significantly better if participants have more time to reflect on their answers?

4. Does webcasting need a talking head and shared computer screen both permanently on? In our webcasting, the presenter could “mix” the camera shot with PowerPoint (ie, decide which was to be seen by the students). Some students commented that this works well. But using an embedded Slideshare presentation and having the talking head always present (as used by Reynolds [11]) is another option in which the slides are clearer. Most commercial packages use that method.

5. Which works best, voice or text chat? This small study and our other studies [23,24] suggest that text chat works well, but some students suggested, and most commercial packages include, the use of voice.

6. How much training or support do lecturers, new to webcasting or similar methods, require? We support the views of others who have noted that technical success is not always followed by organizational adoption of the technology [10]. Yagi et al [7] used webcasting in a large geographically dispersed pathology department and concluded that successful webcasting depends on the creation of a faculty steering committee to control resources and manage growth, the availability of support for technical staff, and embedding the service as part of the core departmental information technology infrastructure. These requirements have currently been met at our university. The module described in this paper ran again starting in September 2009. Webcasting has been adopted for a range of undergraduate [23,24] and postgraduate modules (Heather Skirton, personal communication 3/4/2009), and we have been able to issue some presenter guidelines (Multimedia Appendix 5) to support wider use. However, organizations new to these methods will need to appraise the costs and benefits of such developments.

7. How many students can be engaged in an interactive webcast while keeping it a good experience for the students? Making postgraduate modules cost-effective is a continuing challenge. With our own module, we will aim to recruit more students but to use breakout groups to keep them in the same tutorial group throughout. This will be challenging for the presenters, but we aim to involve other members of staff who are new to eHealth but who are experienced facilitators so that our students have breakout room support while the staff member has a professional development opportunity.

8. Should live webcasting be used to complement live lectures? In the past, we have had TV assistance to webcast live lectures [25], and Baecker et al [20,21], among others, have reported successful use of that approach. Our own view is that this disadvantages the distant webcast audience, who feel like flies on the wall rather than full participants. However, further work into the advantages and disadvantages may be worthwhile.

9. How well are learning outcomes achieved using webcasting compared to other methods?

In conclusion, this model of synchronous e-learning based on interactive live webcasting was a successful method of delivering an international postgraduate module. Students found it engaging over a 10-week course. Although this is a small study, given that synchronous methods such as interactive webcasting are a much easier transition for lecturers used to face-to-face teaching than asynchronous methods, they should be considered as part of the blend of e-learning methods. Further research and development is needed on interfaces and methods which are robust and accessible, and on the most appropriate blend of synchronous and asynchronous work for different student groups.

## Multimedia Appendix 1

Extract of recorded session (video file)

## Multimedia Appendix 2

Extract of chat room conversation (Excel file)

## Multimedia Appendix 3

Email questionnaire

## Multimedia Appendix 4

Illustrative extracts from the collated responses

## Multimedia Appendix 5

Guidelines for presenters using our webcasting (PowerPoint file)

## Abbreviations

NHS: National Health Service in England
TV: television
