# Supplementary material for: Use of Live Interactive Webcasting for an International Postgraduate Module in eHealth: Case Study Evaluation
Source: J Med Internet Res. 2009 Nov 13;11(4):e46. doi: 10.2196/jmir.1225 (PMC2802565; doi:10.2196/jmir.1225)
Supplement: Supplementary file 3 [file jmir_v11i4e46_app3.doc]

Dear

Thanks for taking this module. I wonder if I can ask you for your views about the module to help us improve it for next time. A colleague of ours, Tara Alexander, is helping us with this so that you can respond to her and your identity will remain confidential to Tara. We would also like to submit a paper entitled something like ‘Student and lecturer experiences of using live interactive webcasting for a postgraduate module in e-health’. If we do that we would acknowledge your contribution in the paper. The paper would be authored by Jones, Maramba, Boulos, Alexander with you named in acknowledgements. As such, we regard this as a collaborative report rather than regarding you as ‘subjects’ and we have not sought ethical approval to send you this email. If you wish to comment but do not wish to have your name cited in the acknowledgement please let Tara know and I can add ‘unnamed student’ to the list. We will want to include some idea of geographical spread and job function among the students without being specific (unless you are happy to be specifically named). We will also probably include a few examples of anonymised chat discussion as results. However, we will circulate draft papers for you to comment so you will be able to tell me if you want any wording changed or omitted.

Although we include a few closed questions most are open ended so that you are free to comment. Also feel free to answer questions that we did not ask! Please be honest rather than kind. By sending your responses to Tara you can happily say (eg) say Ray did not speak clearly enough, or Ray’s sessions were boring and did not cover enough material........whatever. Tara will remove your name before passing on aggregated/synthesised comments to the three of us (me, Cito, Maged).

Finally, after anonymising and synthesising the data from your responses we will post a summary on the module portal and invite you to check it and add any further comments.

Please use the numbering and questions as headers for your replies. To remind you of the sessions and so that you can refer to sessions by title or number there is a list at the end of this document.

1. **Overall**
2. What did you like and dislike about the module?
3. On the whole have you found the module useful (closed question): [ ] Very useful, [ ] Useful, [ ]OK/no definite opinion, [ ] Not very useful, [ ] Not at all useful
4. Would you recommend the module to others amongst your colleagues? If so which job functions or roles?
5. (Not for UoP Phd students). For you and whoever paid for you, was it worth the money?
6. (For UoP PhD students only). Do you think this module was worth £220 (compared to other courses or modules you may have taken)?
7. The income for the University of Plymouth from your fees only does not recompense the time put into the module. If we were to increase the charge to (say) £400 and also have more students (say 50 students) do you think it would be value for money? If we were to make more use of asynchronous materials (eg some of the webcast recordings from this year) do you think the quality of the sessions would decrease substantially?
8. **Method of delivery (ie live webcasting, chat room, recordings on the portal etc)**
9. In general, did this method of delivery ‘work’ for you?
10. Synch Vs asynch:
    1. Have you had experience of giving or using asynchronous e-learning in the past? (closed question, tick all that apply): [ ] As a teacher [ ] As a student [ ] No
    2. What do you think are the advantages and disadvantages of the method we used compared to asynchronous e-learning? You can answer even if you didn’t have much experience of those other methods. Also, you can answer both from the point of view of teacher or student.
    3. Closed question. Was the timing of the sessions (2-4.30 British Time) Very convenient/convenient/so-so/not very convenient/not at all convenient.
11. Break out groups: This may vary by session and presenter. Feel free to comment on individual sessions or presenters.
    1. Did you have too few or too many break out group discussions?
    2. Was there enough/too much opportunity for interaction between students?
    3. Was there enough/too much opportunity to ask questions make comments to the presenter?
12. Pace: This may vary by session and presenter. Feel free to comment on individual sessions or presenters . On the whole was the ‘pace’ too slow/too fast? Should there have been more/less time for reflection and discussion?
13. Screen layout: Do you have any views/ideas on how we could make better use of the screen (eg layout, features, etc)?
14. Audio Vs text. Some authors have used audio interaction with participants rather than text chat? Do you have any views on the advantages or disadvantages of each?
15. Robustness and technical difficulty. What were the main problems that you had, ie which are the most important things to address to make this method more accessible?
16. Connectivity with other students. Did you feel ‘connected’ with the other students on the course? Did you want to be more/less connected to the other students? What contributed to the connectivity or lack of it?
17. Downloads: Did you download the presentations and papers for a session before/after/sometimes before and sometimes after the session? Did you use them before/during/after webcasts or not used them (yet)?
18. Portal: Do you have any views on the student portal?
19. Video window: Do you have any views on the way we used the video window, such as the quality of the powerpoint slides, the use of fade between talking head and slides, relevance of each etc
20. Presentational style: Do you have any comments on the three presenters (Ray, Maged, Cito) and our presentational styles? Do you have any suggestions on how we could improve our presentations?
21. Overall delivery method: If we offered other modules with relevant content for you, would you be interested in taking them using this same method? Yes/No
22. **Content**
23. Which sessions were most and least relevant for you?
24. Was there other content or other sessions that you would have liked?
25. If we reduced the length of the course to (say) 6 sessions, which would you have dropped? Why?
26. **Assessment**
27. Was the assessment (one piece of coursework and exam) appropriate to the module?
28. (Closed question). Was it the assessment (coursework plus exam) too heavy/about right/too light in your view for this 10 credit postgraduate module?
29. Do you think the exam (in week 10) was an appropriate time (i.e. while you still remember, ’get it out of the way’) or would you have preferred it after a longer period? (if so when?)
30. If you were running a module like this, would you have any concerns about the ‘open book’ distance nature of the assessment?
31. Do you have any other comments about the assessment method?
32. **Future and other possibilities**
33. We may run the module for other institutions in which small groups watch the webcast with one group member acting as scribe for their group’s interaction with other groups. Do you have any views on this possibility?
34. We may use webcasting for patient education. Do you have any views on this possibility?
35. Are there some other questions we should have asked or other comments that you would like to make?
36. **Marketing and Promotion**
37. How did you hear about the course?
38. If it was word-of-mouth are you aware of other sources where it was advertised? If so what were they?
39. Was the information provided on enquiry useful? If no, what recommendations would you make to change it for the better?
40. Please recommend any sources of where you think this module should be advertised.

**THANKS VERY MUCH INDEED FOR YOUR RESPONSES**

To remind you of the sessions

| Session 1 | Wednesday 8th October 2008 | Ray | Introduction; definitions and scope of e-health; historical development of consumer health informatics; computer-patient interviewing. |
| --- | --- | --- | --- |
| Session 2 | Wednesday 15th October 2008 | Ray | Patient and public use of information; Tailoring of information. Ethical, legal, practical issues in giving patients access to their electronic record. Integration of patient’s use of computers into clinical practice and learning needs of clinicians. Quality and trust: web sites, peer-peer. |
| Session 3 | Wednesday 22nd October 2008 | Ray and Cito | Design of ICT based learning programmes for students, professionals and patients. Access to and evaluation of those programmes. Shared learning Patient, public, and professional use of synch/asynch methods for support. Virtual focus groups. |
| Session 4 | Wednesday 29th October 2008 | Maged | Virtual worlds |
| Session 5 | Wednesday 5th November 2008 | Ray | Public health and e-health. Using information for health needs assessment. Evaluation methods in e-health, outcomes, research designs and problems in e-health. Issues of equity: older people, deprivation, rurality. |
| Session 6 | Wednesday 12th November 2008 | Maged | Geographic information systems, confidentiality in using personal data for epidemiology and planning |
| Session 7 | Wednesday 19th November 2008 | Cito | Quality of Health Information on the Internet. Health Literacy. Ethics. |
| Session 8 | Wednesday 26th November 2008 | Ray | Student-presentations |
| Session 9 | Wednesday 3rd December 2008 | Maged | eHealth and telehealthcare: a panacea for Europe's socio-demographic changes in the 21st century?-- the reality, barriers and challenges ahead |
| Session 10 | Wednesday 10th December 2008 | Ray | Exam. 3 hours. Answer 2 questions (open book) from choice of 6 |
