# Supplementary material for: Use of Live Interactive Webcasting for an International Postgraduate Module in eHealth: Case Study Evaluation
Source: J Med Internet Res. 2009 Nov 13;11(4):e46. doi: 10.2196/jmir.1225 (PMC2802565; doi:10.2196/jmir.1225)
Supplement: Supplementary file 4 [file jmir_v11i4e46_app4.pdf]

On the whole have you found the module useful (closed question):

[ ] Very useful = 5
[ ] Useful = 7
[ ]OK/no definite opinion = 2
[ ] Not very useful = 0
[ ] Not at all useful = 0

Would you recommend the module to others amongst your colleagues? If so which job functions or roles?

Yes = 14

Comments:
All those working in health // gave non-specific list = 4

Recommend to those interested in e Health as a teaching/learning/research/ management activity = 2

Clinicians, nurses, informatics personnel, allied health professionals // direct contact with patients = 5

Producing information // communicating with service users, web developers // Marketing / PR // business analysis = 3

Senior journalists =1

Have you had experience of giving or using asynchronous e-learning in the past? (closed question, tick all that apply):

[ ] As a teacher = 2
[ ] As a student = 10
[ ] No = 3

[Students answered multiple items]

Screen layout: Do you have any views/ideas on how we could make better use of the screen (eg layout, features, etc)?

I liked the layout N=4
Make the viewer bigger N=4
Make layout customisable: N=1
Text too small: N=2

Comments:

I occasionally had problems when using Mozilla but switching to IE sorted these out. For example, in Mozilla rather than viewing the last comment the side bar would jump up and I would only be presented with the conversation several pages/minutes before.

I think it was <staff member> who had the slide-show running in a separate area alongside the video stream. This worked quite well, although it reduced the space available for the chat window. If you can find a way of accommodating all three things, that'd be ideal, but on balance I think a large chat area is more important than providing access to the slides from within the web application.

The quality of the power point on the screen was not that good. Unfortunately, some of the presentations offered by the presenters were Power point slideshow and thus opens in a full screen mode thus causing me not to follow with the presentation. Alternatively had to settle for the onscreen presentation.

- The chat box could use some improvement as mentioned before
- In some cases, we lost sound or picture because of the set-up on the lecturer's end, and though people were typing in the chat box that the sound was still turned off, sometimes the lecturer didn't notice because they were looking at a different screen. Not sure if the layout then can be improved somehow on the delivery side?

Ability to input into discussion does not have the priority it deserves. Needs to be visually stronger as is a key task for student

<u>Audio Vs text.</u> Some authors have used audio interaction with participants rather than text chat? Do you have any views on the advantages or disadvantages of each?

Prefer audio: N =4
Prefer text N= 9

<u>Comments:</u>
The advantage of text is that we could all speak simultaneously; Text was also instantaneous, bearing in mind that some participants were at the other side of the world, I am sure that there would be problems with audio feeds. Text works. We are all, increasingly, becoming used to typing/ texting and it is a comfortable medium. However, that English may not be the first language for all.

I do like audio, but then you need a "hand raising" tool too, like in e Class.

<u>Connectivity with other students</u>. Did you feel 'connected' with the other students on the course? Did you want to be more/less connected to the other students? What contributed to the connectivity or lack of it?

Felt connected: 11
So-so: N=3
Did not: N=0

<u>Comments</u>:

[Felt connected] if we wound up in the same breakout rooms a bit more often X2.

Because we were given only the option of writing our comments, they were recorded for future scrutiny.

There were times in the chat rooms, when I felt very isolated; the others were chatting and my comment/query was missed in the exchange. Sometimes this led me to think that my input was not valid/valued.

Would it be possible to have information about people closer at hand i.e. when someone has commented, it's hard to remember who they are (where they are from and what they do.

I felt Connected and respected. The pace was set by the presenters – they greeted everyone as if they were equally important and welcomed.

<u>Video window:</u> Do you have any views on the way we used the video window, such as the quality of the powerpoint slides, the use of fade between talking head and slides, relevance of each etc

Was OK// no suggestions: 4
Make viewer larger: N=4
Increase font size: N=2
No answer = 1

<u>Comments:</u>
I liked the music

The music was ghastly but very necessary as it was an easy way of knowing that the link was active.

Some presenters used fade in or other transitions – these were very off-putting! Perhaps a style guide could be put together ie minimum font size, request/rule for busy slides to be split and not squeezed into the space, background, transition etc.

I liked the simplicity of the slides onscreen but having the presentation available as well

meant that the complicated ones weren’t lost as they could be studied after or on printout or in a different window. Our choice.

<staff member> struggled a bit with the fade option.

The fading back and forth was very effective when used. It not only gave a bit of a feeling of “interactivity”, but also broke up the slideshow nicely (great for people with shorter attention spans and a million thoughts a minute like myself!)

Fade was good. PPT slides fine,. Nice to see real face now and then rather than disembodied slides/voice.
