# Supplementary material for: Use of Live Interactive Webcasting for an International Postgraduate Module in eHealth: Case Study Evaluation
Source: J Med Internet Res. 2009 Nov 13;11(4):e46. doi: 10.2196/jmir.1225 (PMC2802565; doi:10.2196/jmir.1225)
Supplement: Supplementary file 5 [file jmir_v11i4e46_app5.ppt]

## Slide 1
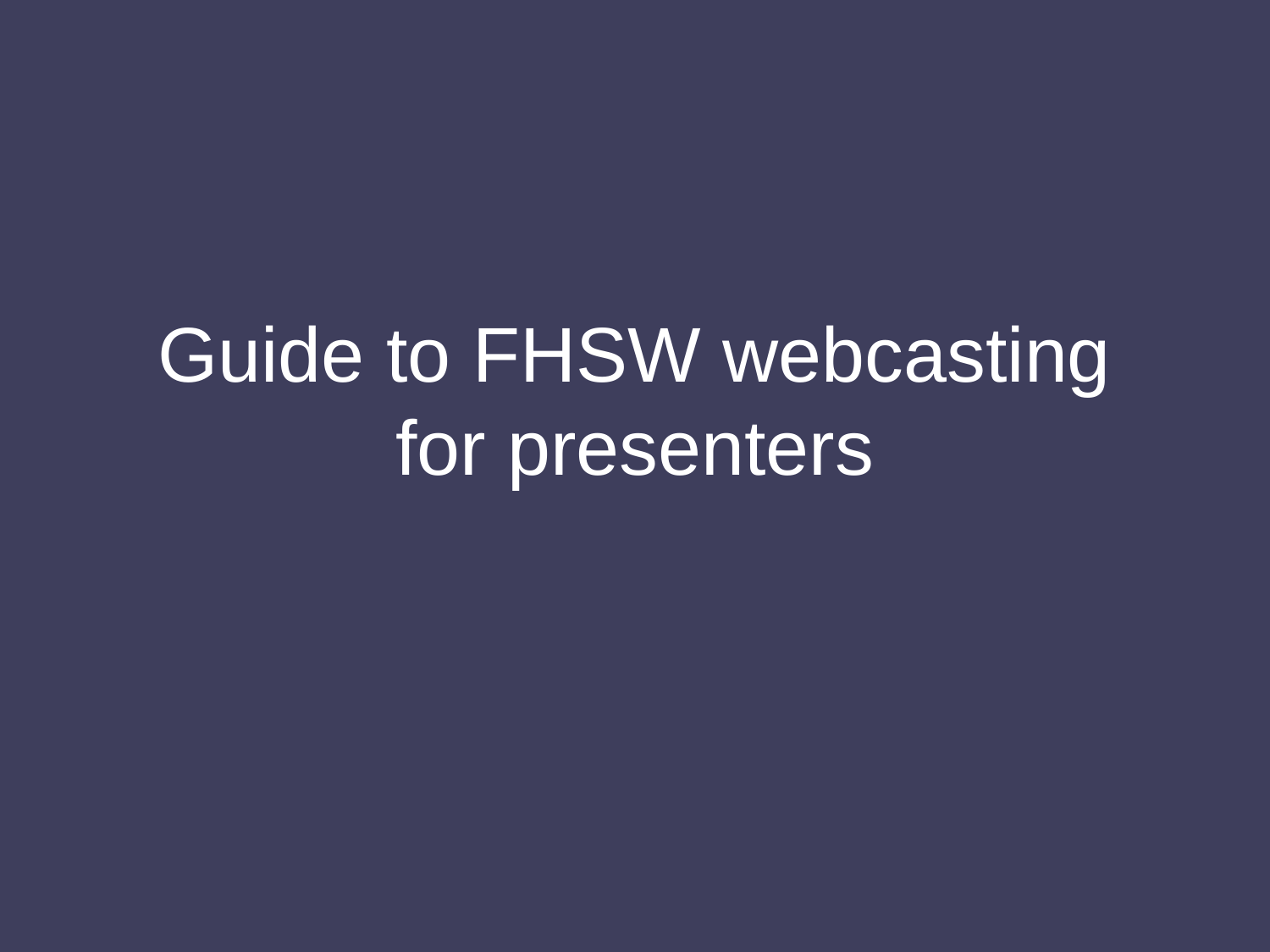

# Guide to FHSW webcasting for presenters

## Slide 2
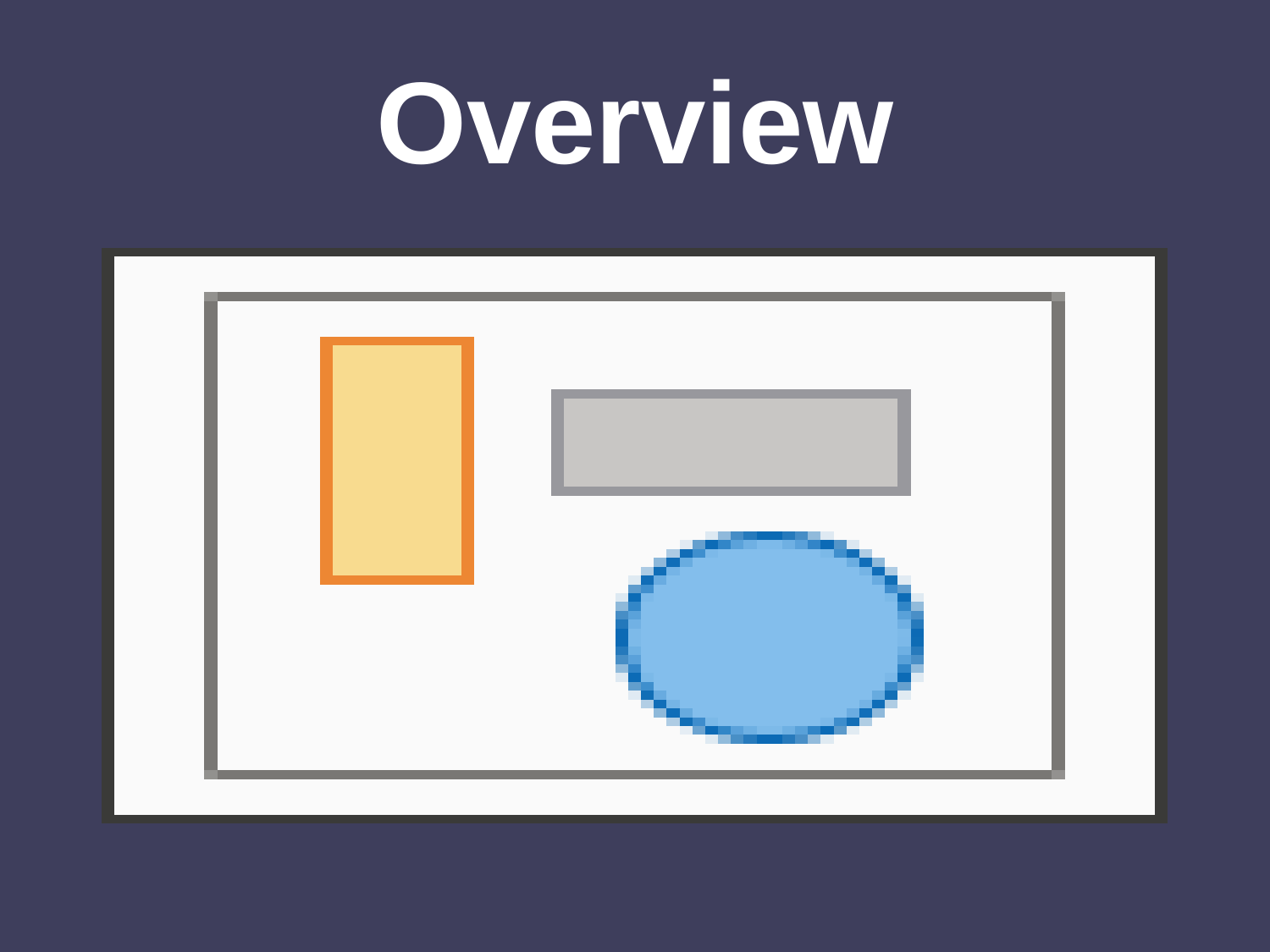

# Overview

## Slide 3
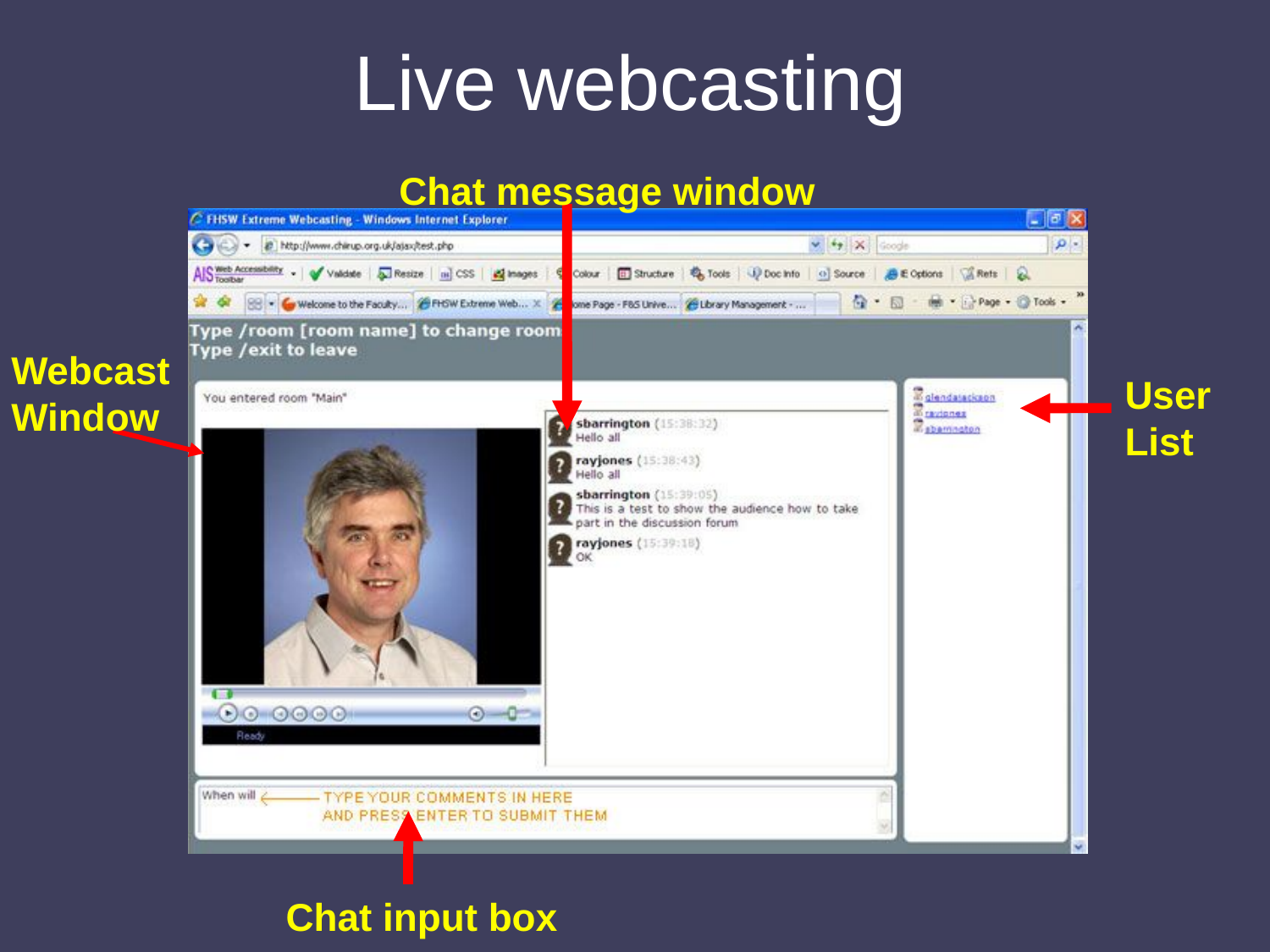

# Live webcasting
Chat message window
Webcast Window
User List
Chat input box

## Slide 4
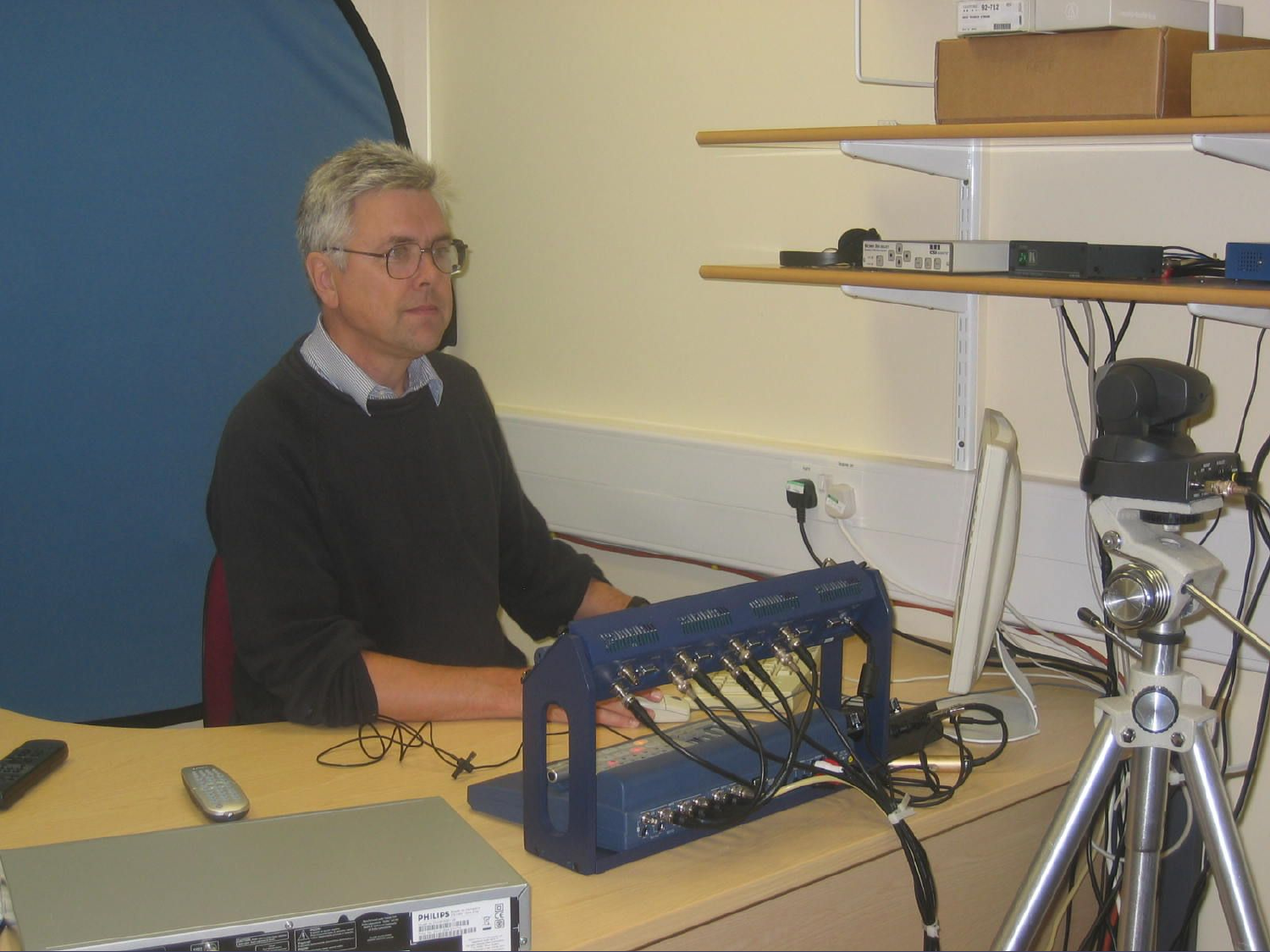

#

## Slide 5
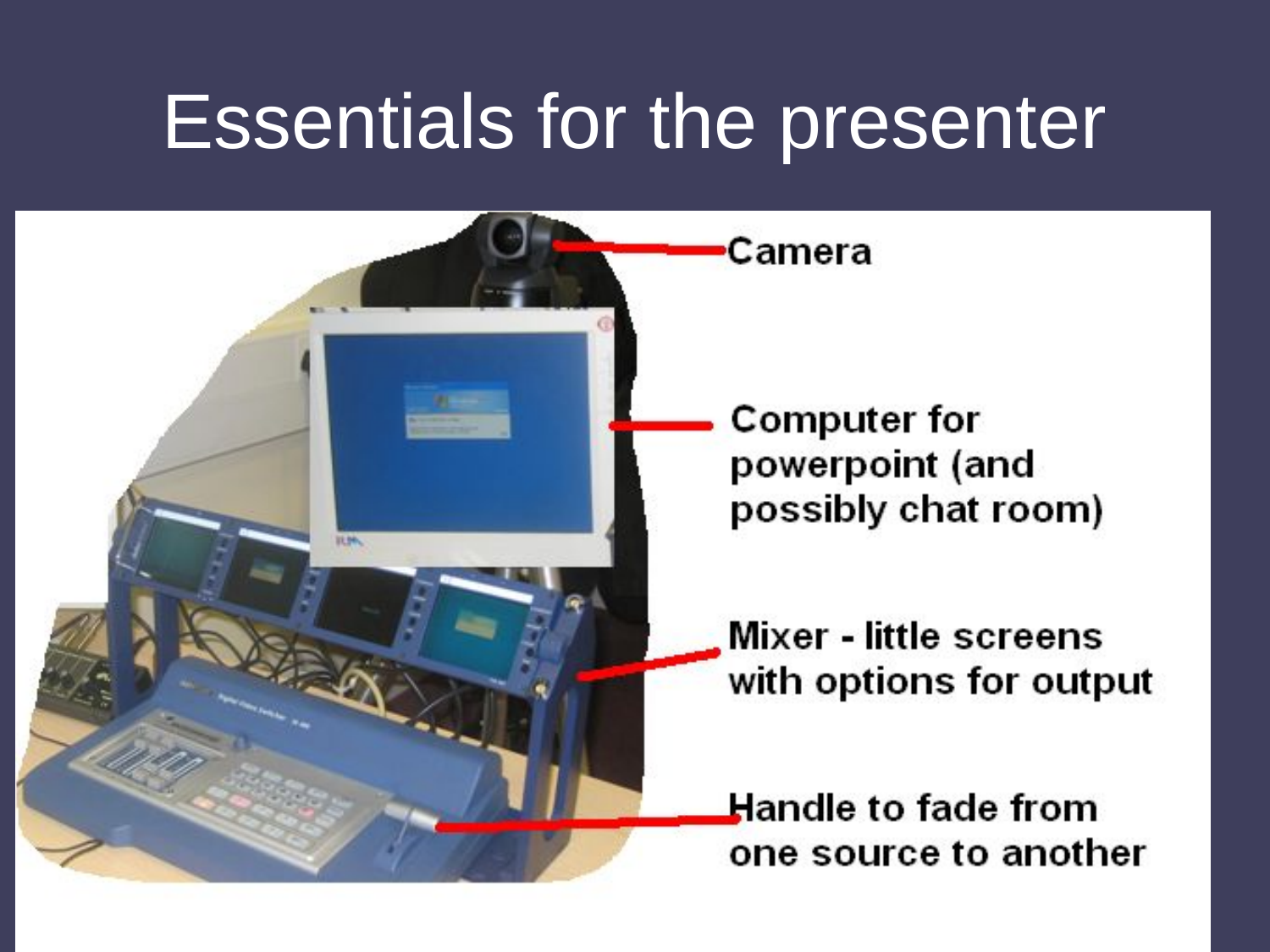

# Essentials for the presenter

## Slide 6
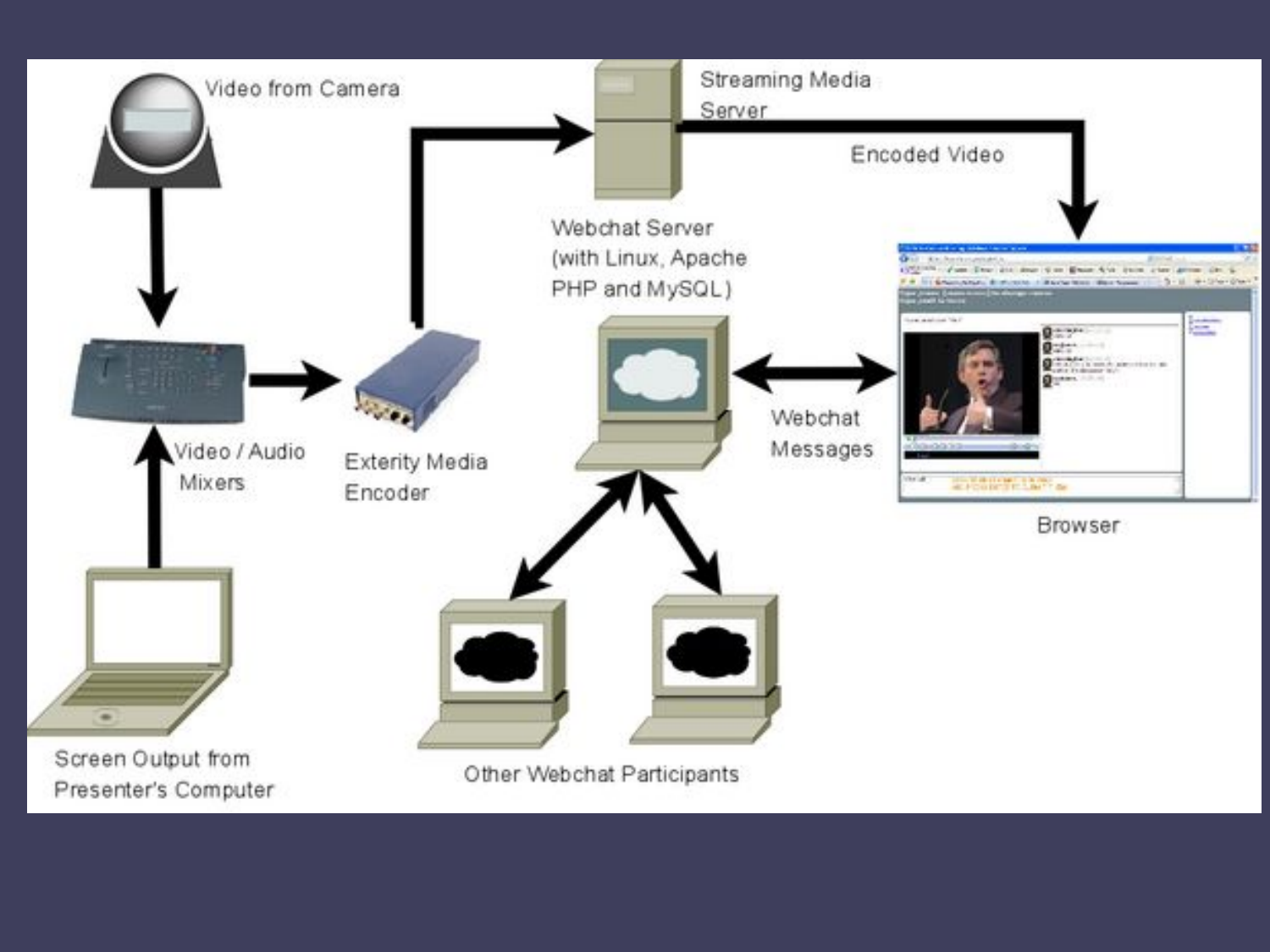

#

## Slide 7
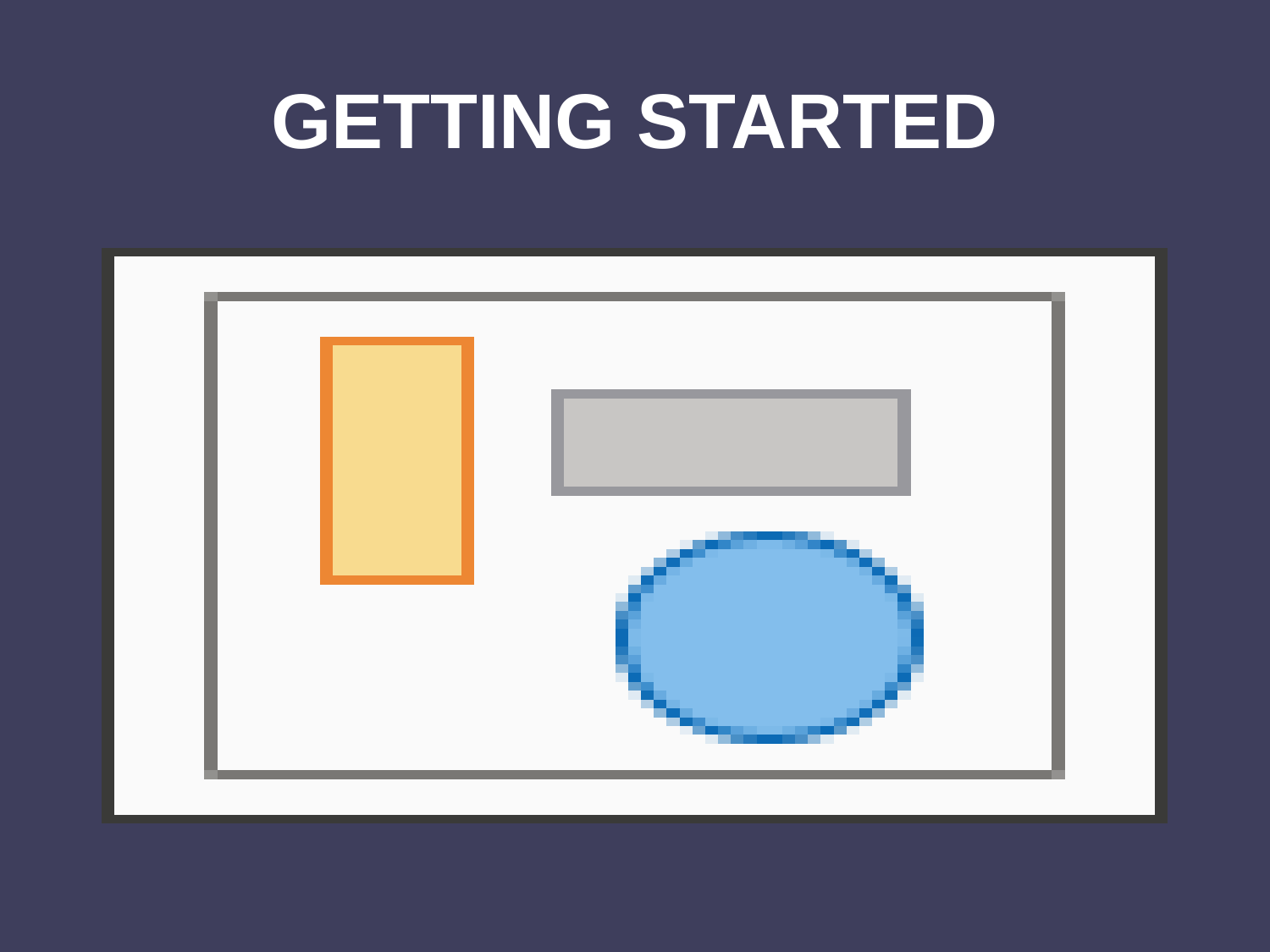

# GETTING STARTED

## Slide 8
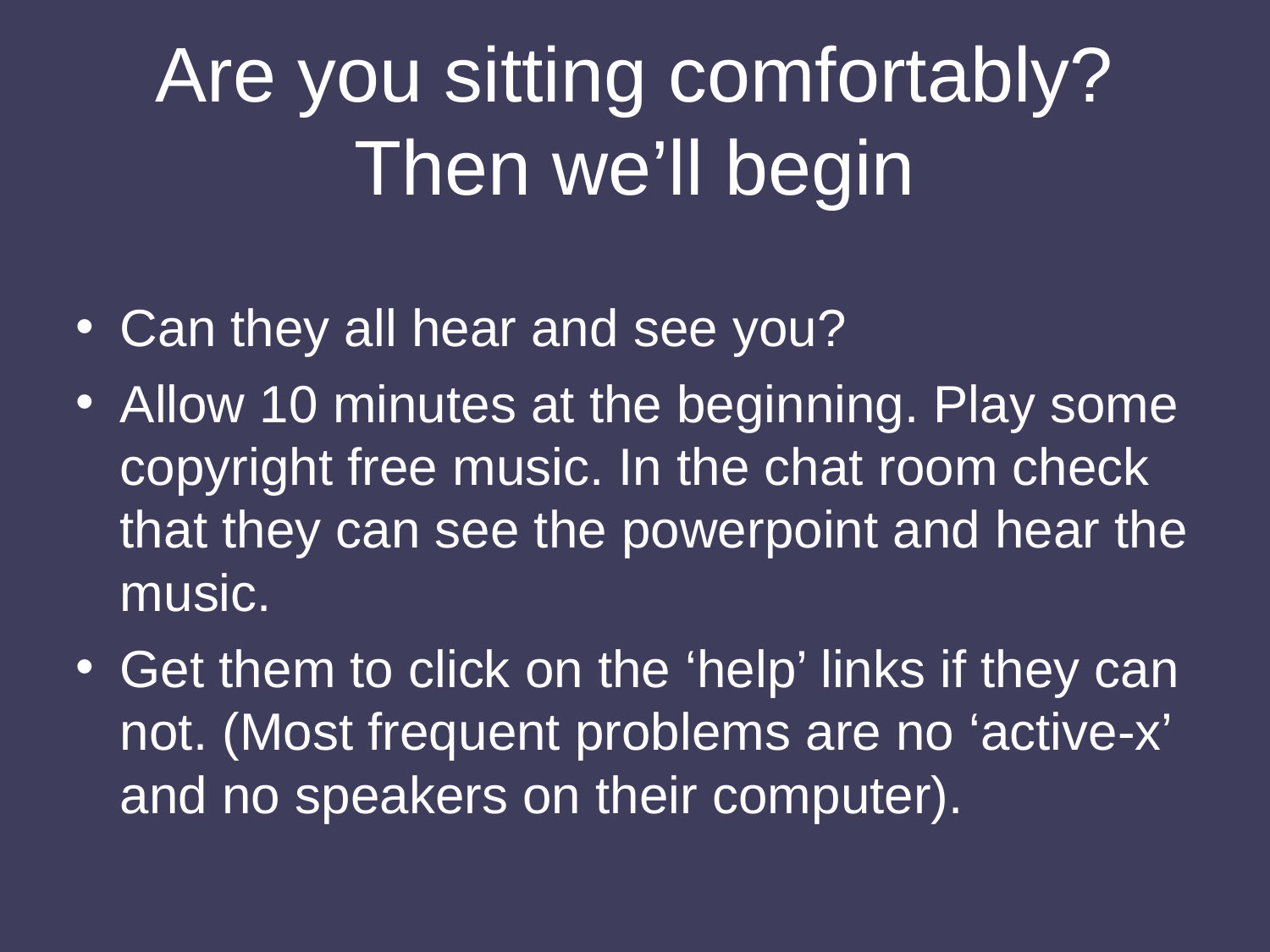

# Are you sitting comfortably? Then we’ll begin
Can they all hear and see you?
Allow 10 minutes at the beginning. Play some copyright free music. In the chat room check that they can see the powerpoint and hear the music.
Get them to click on the ‘help’ links if they can not. (Most frequent problems are no ‘active-x’ and no speakers on their computer).

## Slide 9
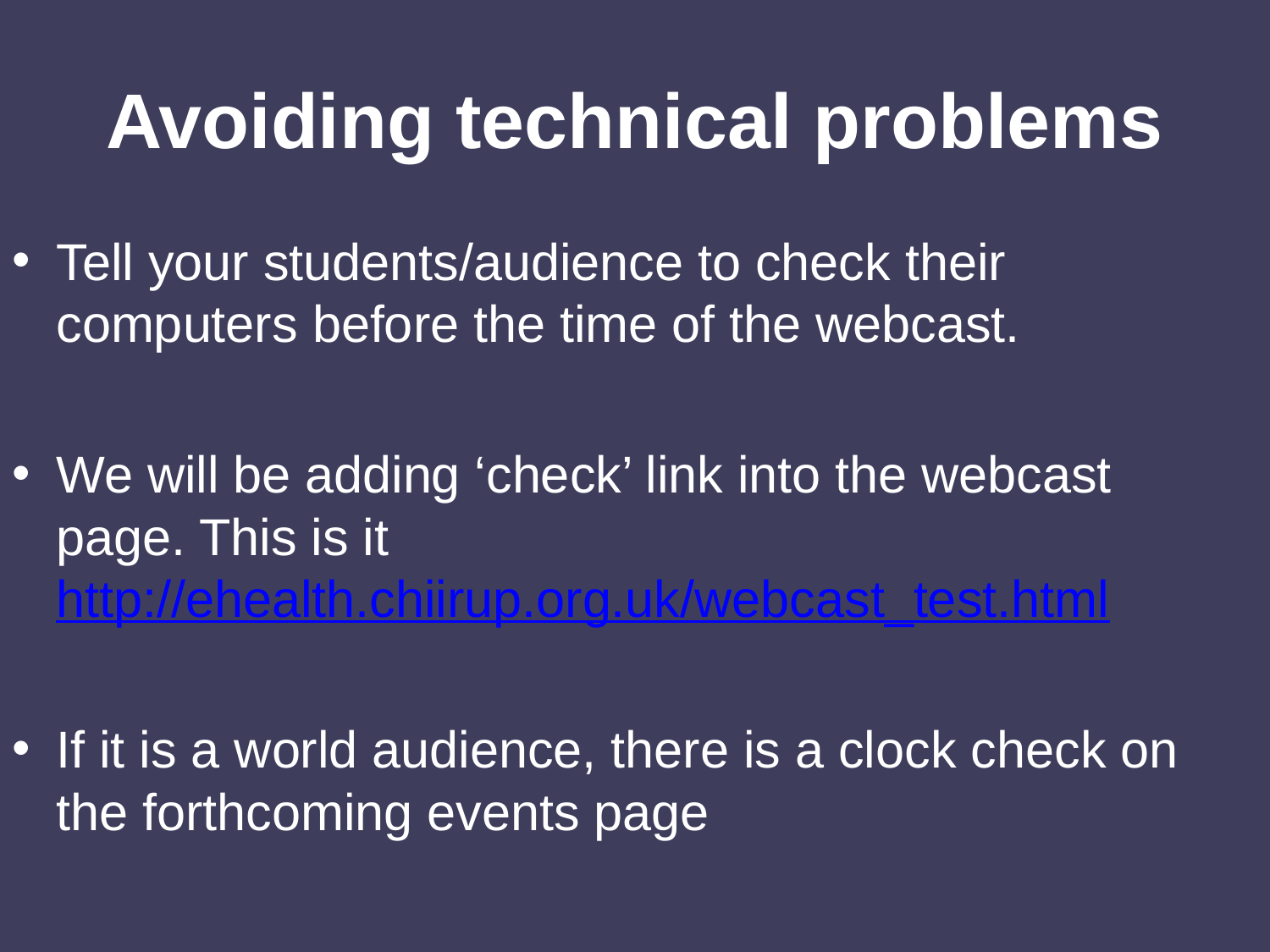

# Avoiding technical problems
Tell your students/audience to check their computers before the time of the webcast.
We will be adding ‘check’ link into the webcast page. This is it http://ehealth.chiirup.org.uk/webcast_test.html
If it is a world audience, there is a clock check on the forthcoming events page

## Slide 10
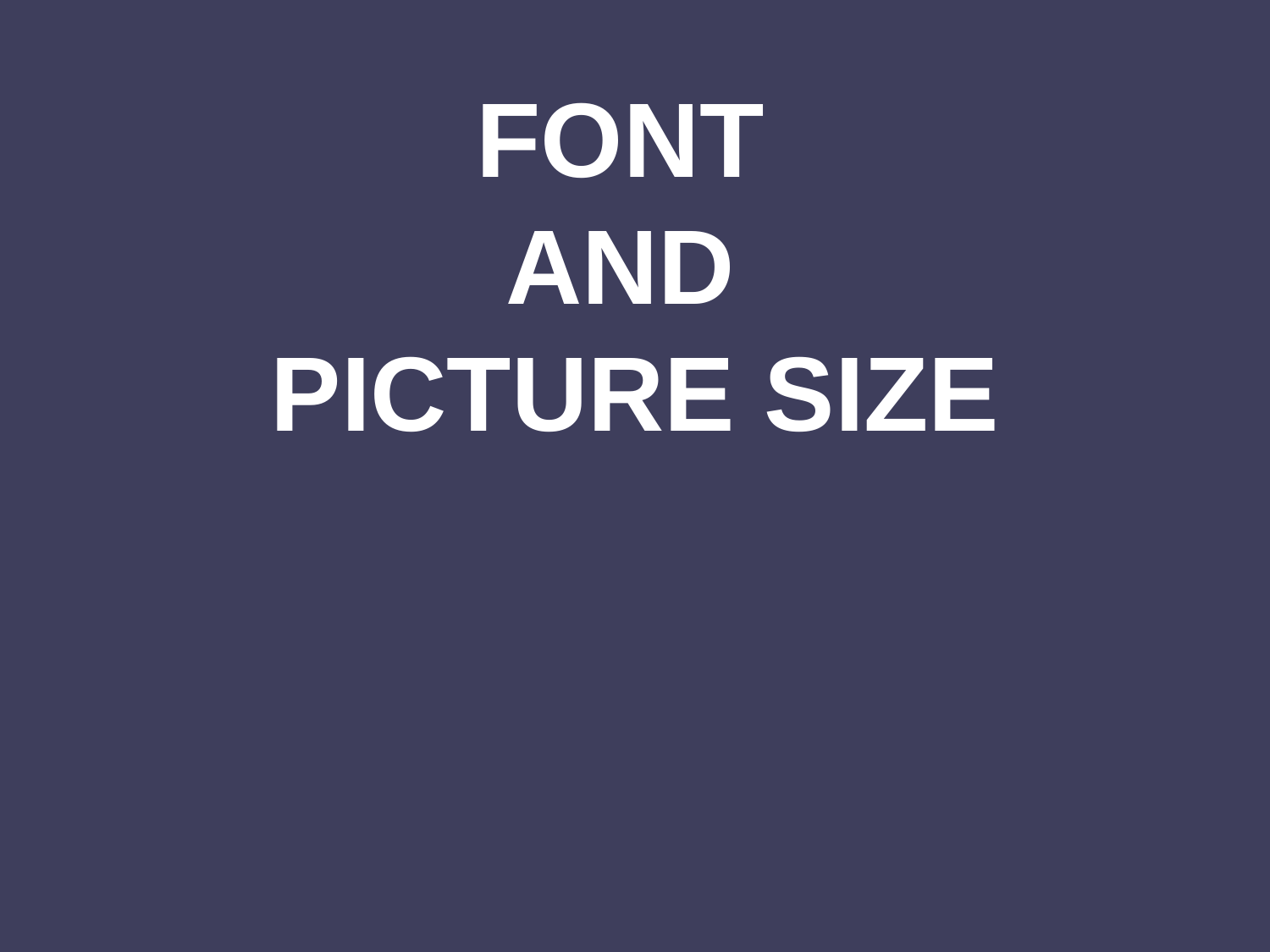

# FONT AND PICTURE SIZE

## Slide 11
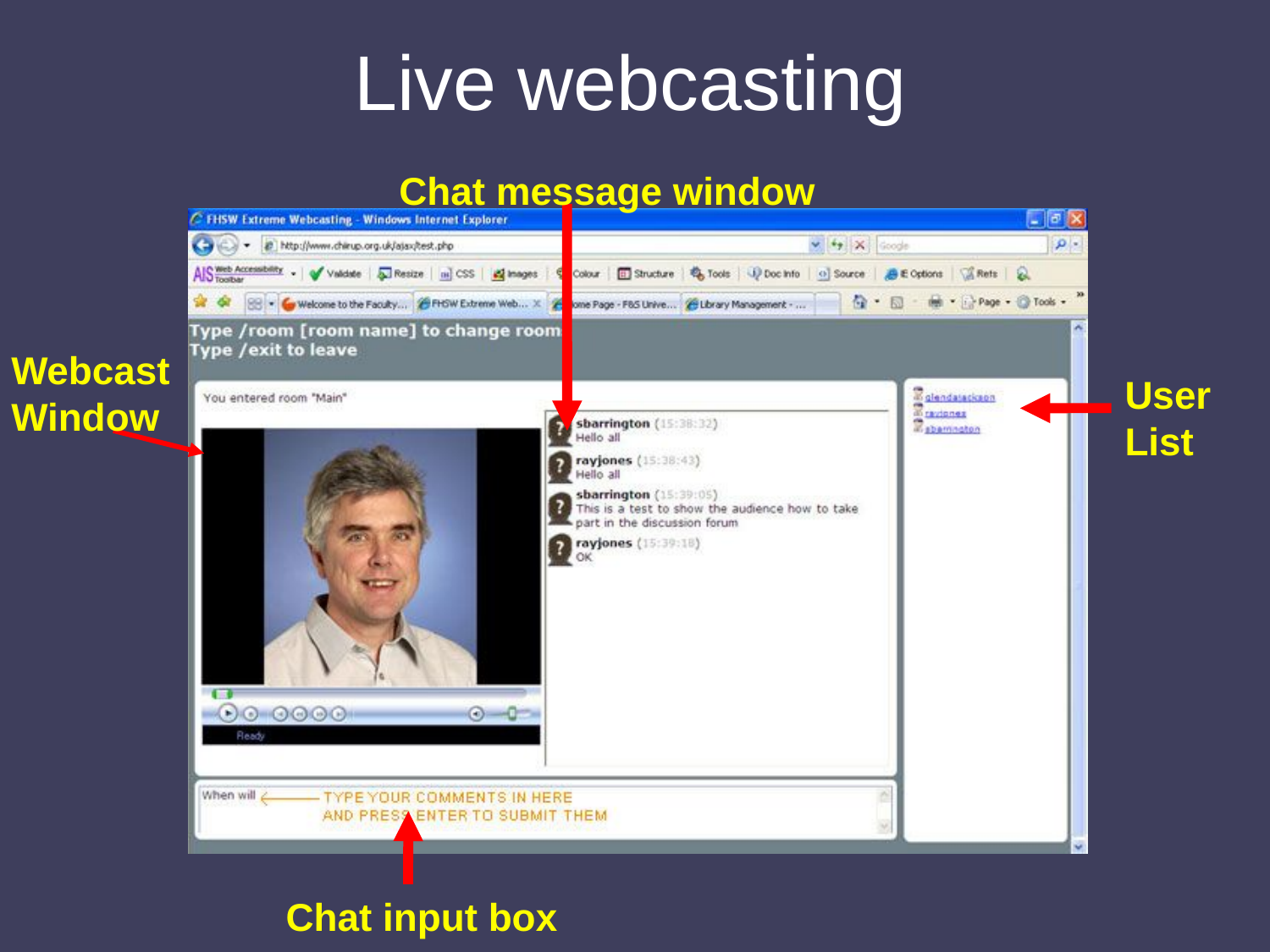

# Live webcasting
Chat message window
Webcast Window
User List
Chat input box

## Slide 12
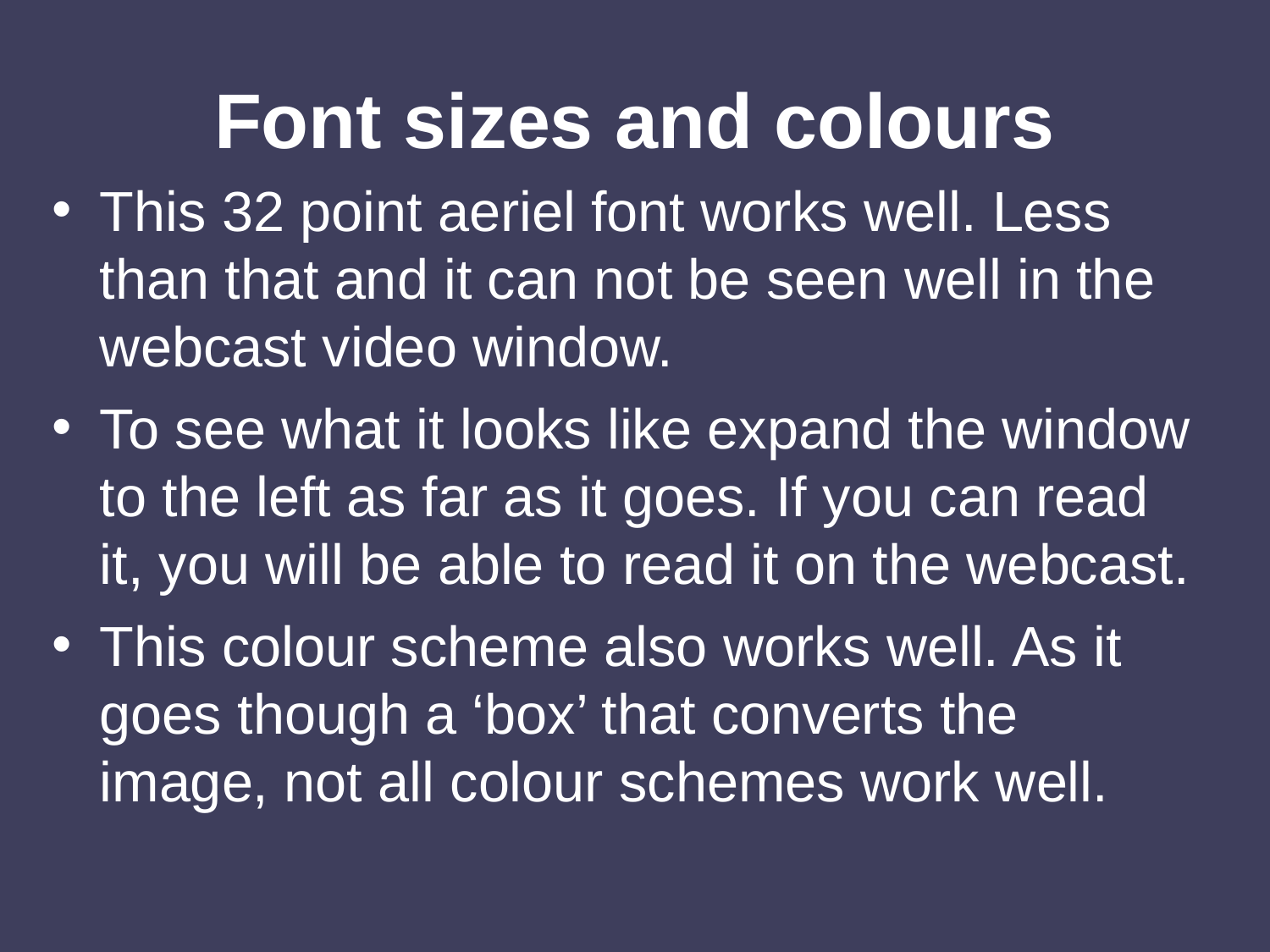

# Font sizes and colours
This 32 point aeriel font works well. Less than that and it can not be seen well in the webcast video window.
To see what it looks like expand the window to the left as far as it goes. If you can read it, you will be able to read it on the webcast.
This colour scheme also works well. As it goes though a ‘box’ that converts the image, not all colour schemes work well.

## Slide 13
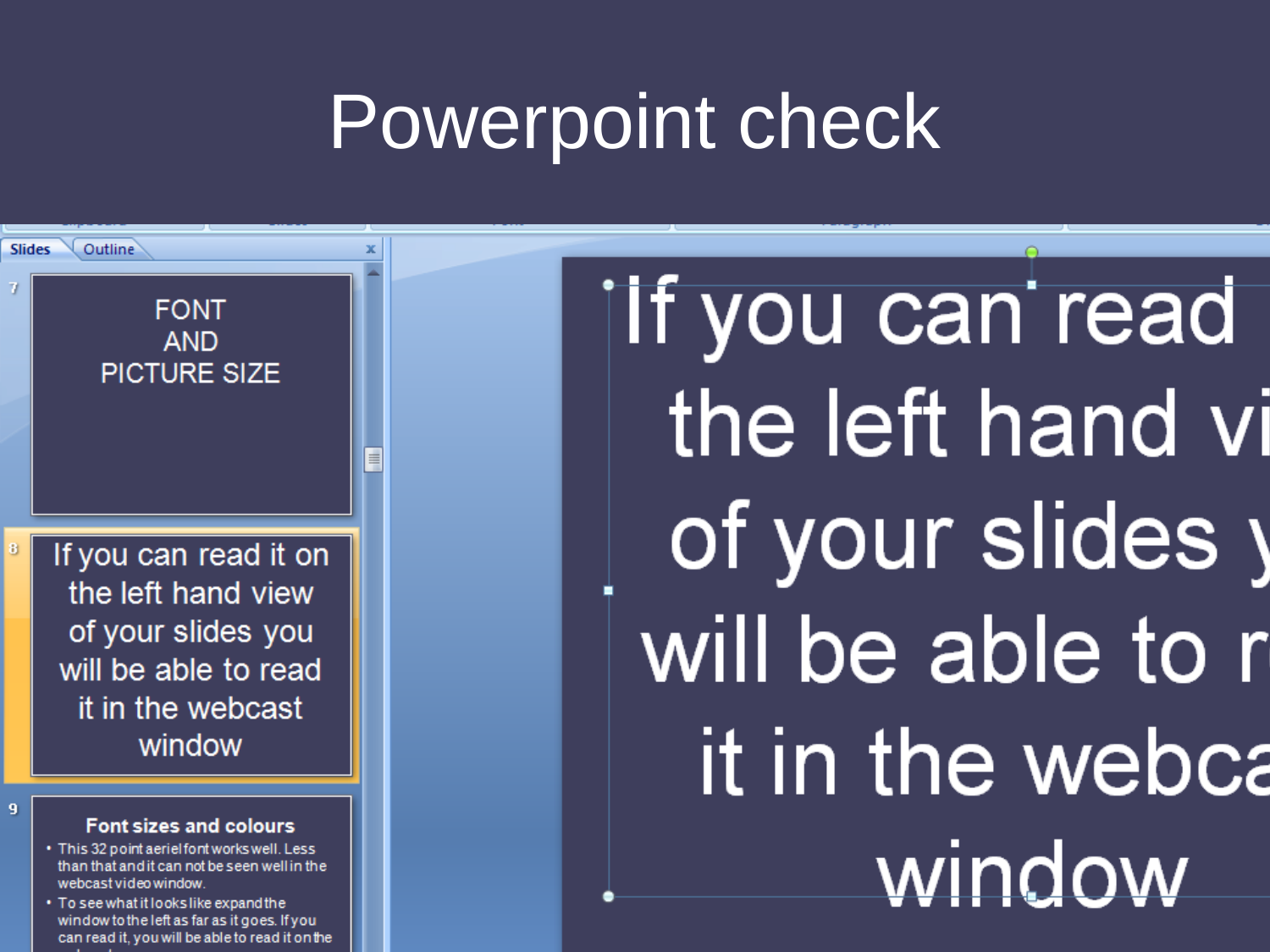

# Powerpoint check

## Slide 14
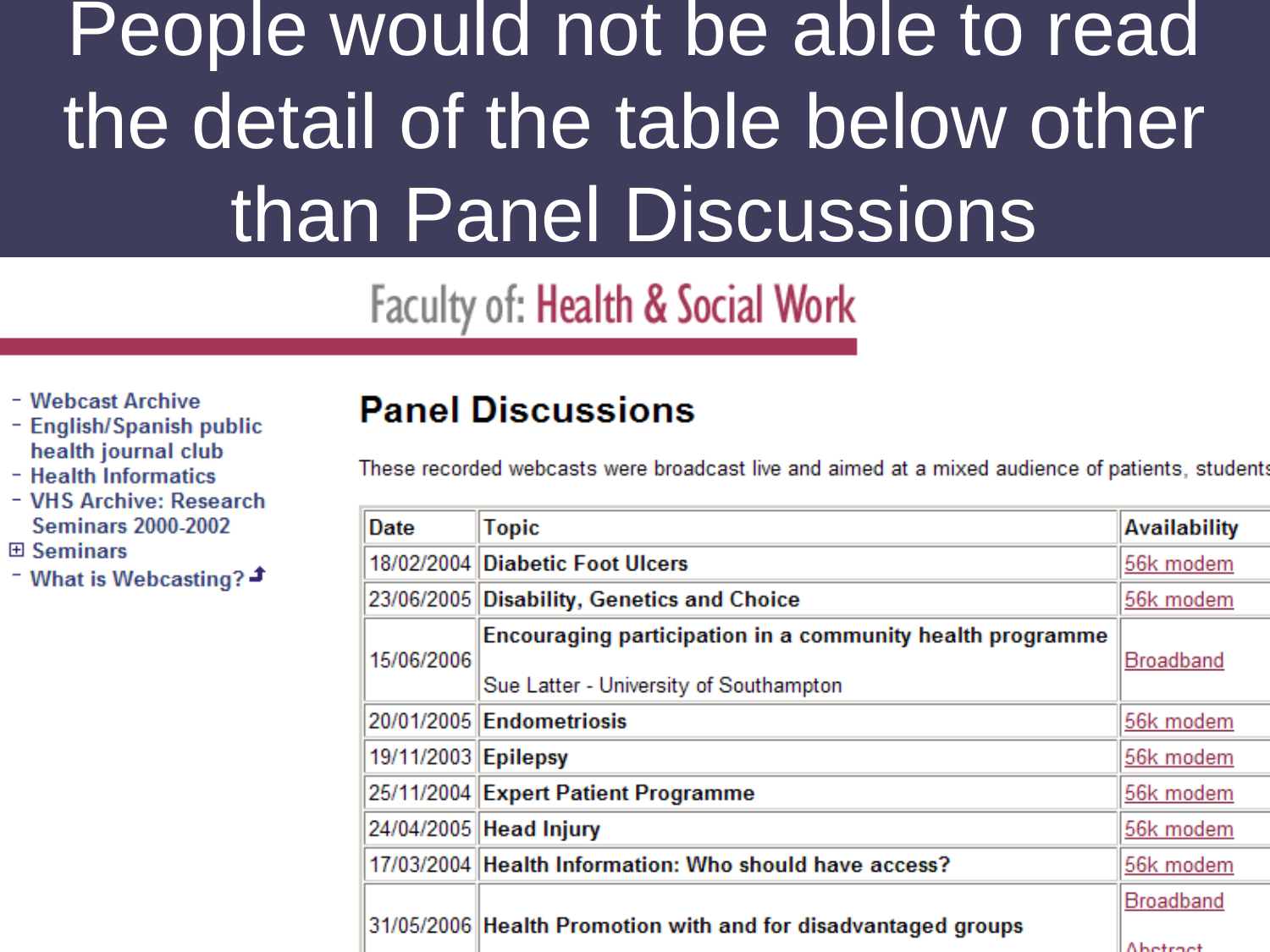

# People would not be able to read the detail of the table below other than Panel Discussions

## Slide 15
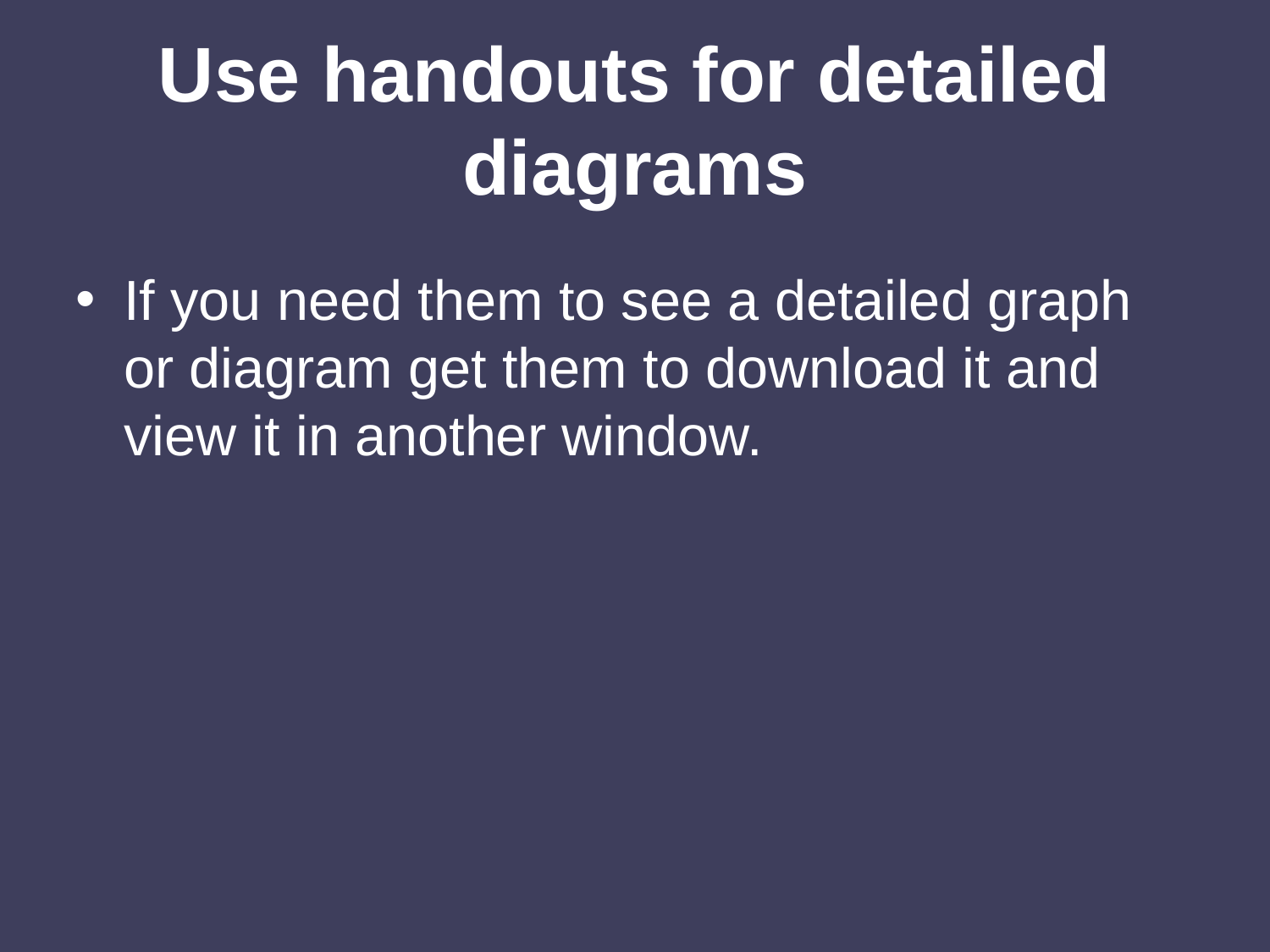

# Use handouts for detailed diagrams
If you need them to see a detailed graph or diagram get them to download it and view it in another window.

## Slide 16
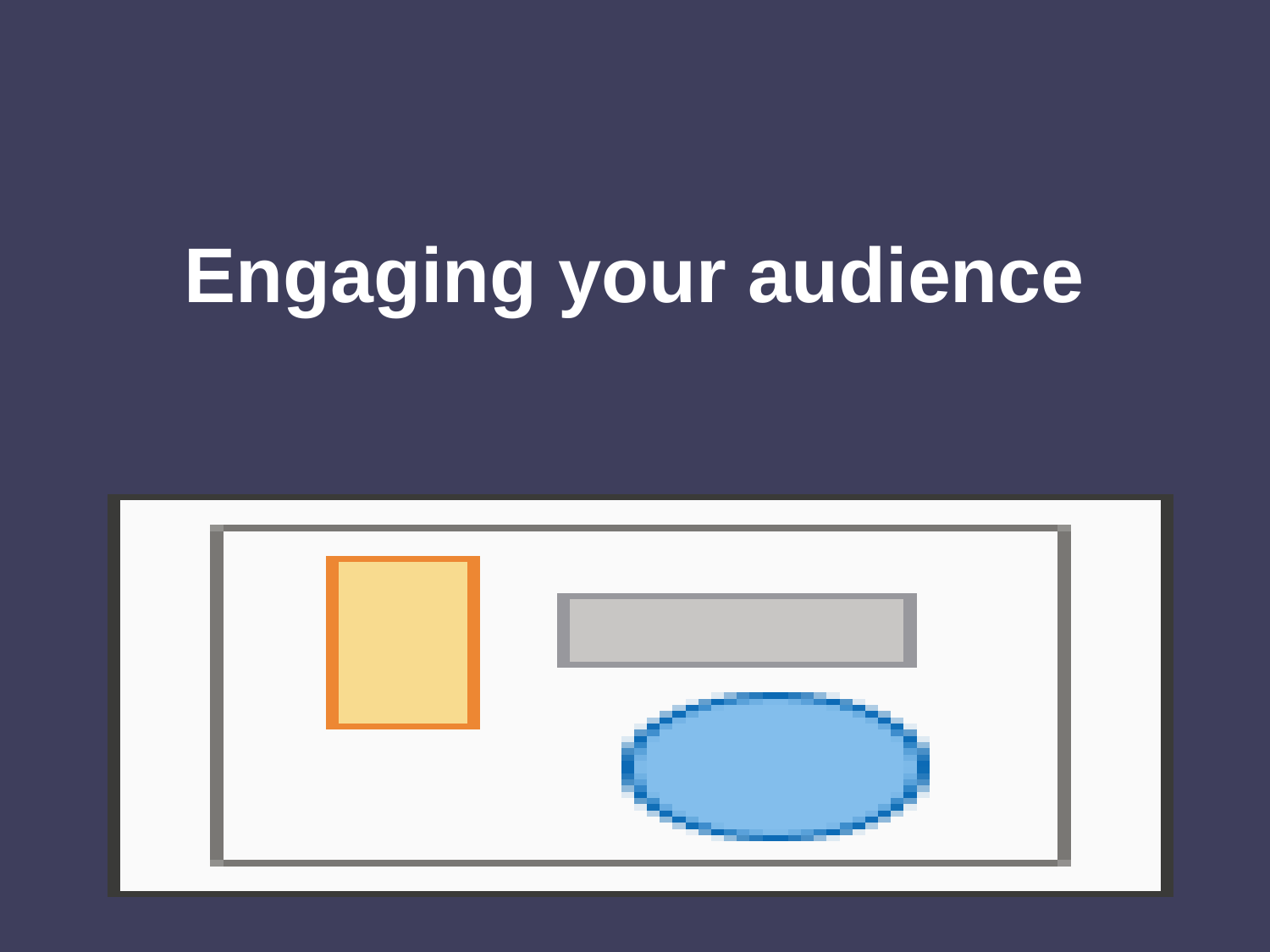

# Engaging your audience

## Slide 17
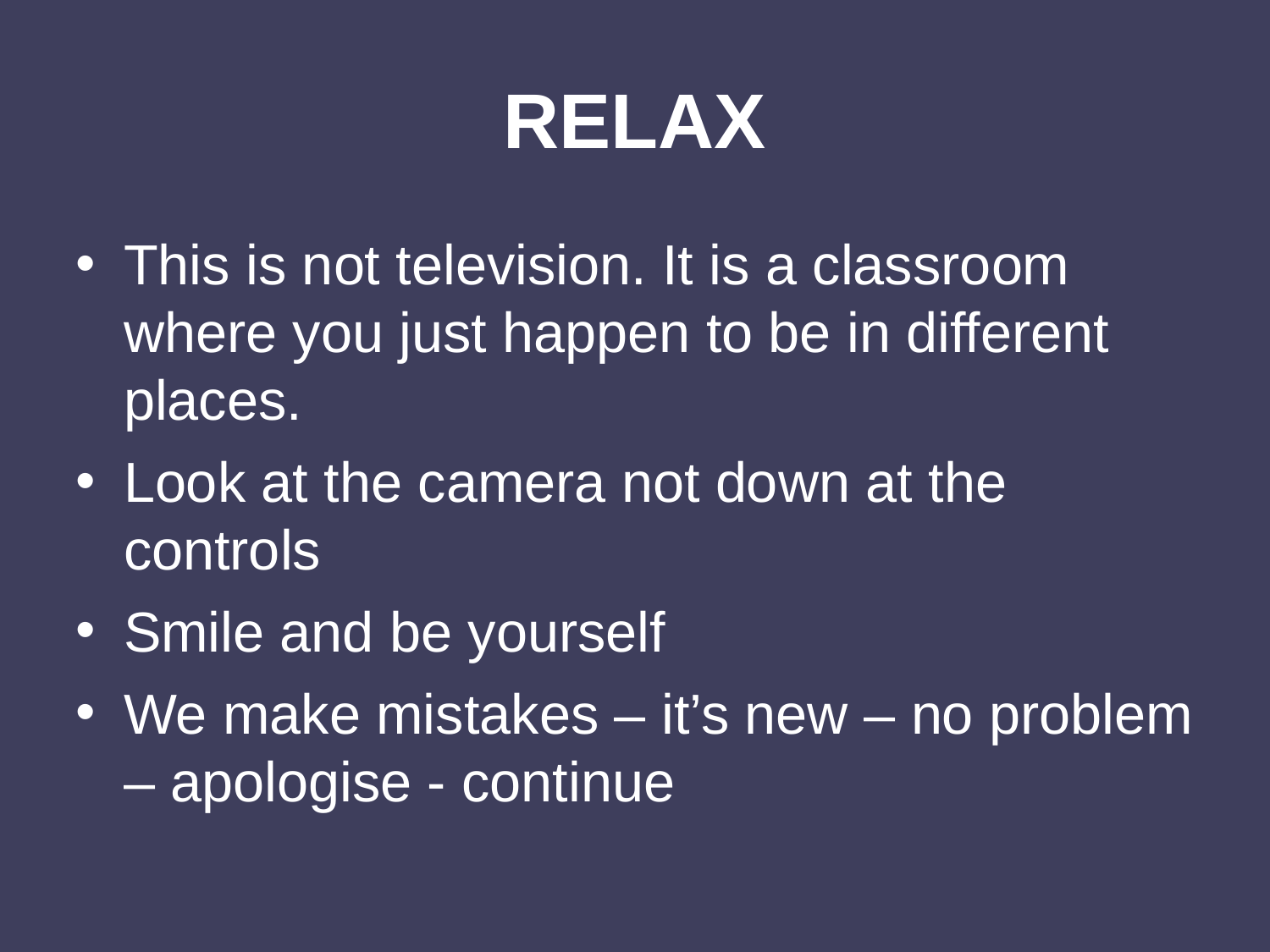

# RELAX
This is not television. It is a classroom where you just happen to be in different places.
Look at the camera not down at the controls
Smile and be yourself
We make mistakes – it’s new – no problem – apologise - continue

## Slide 18
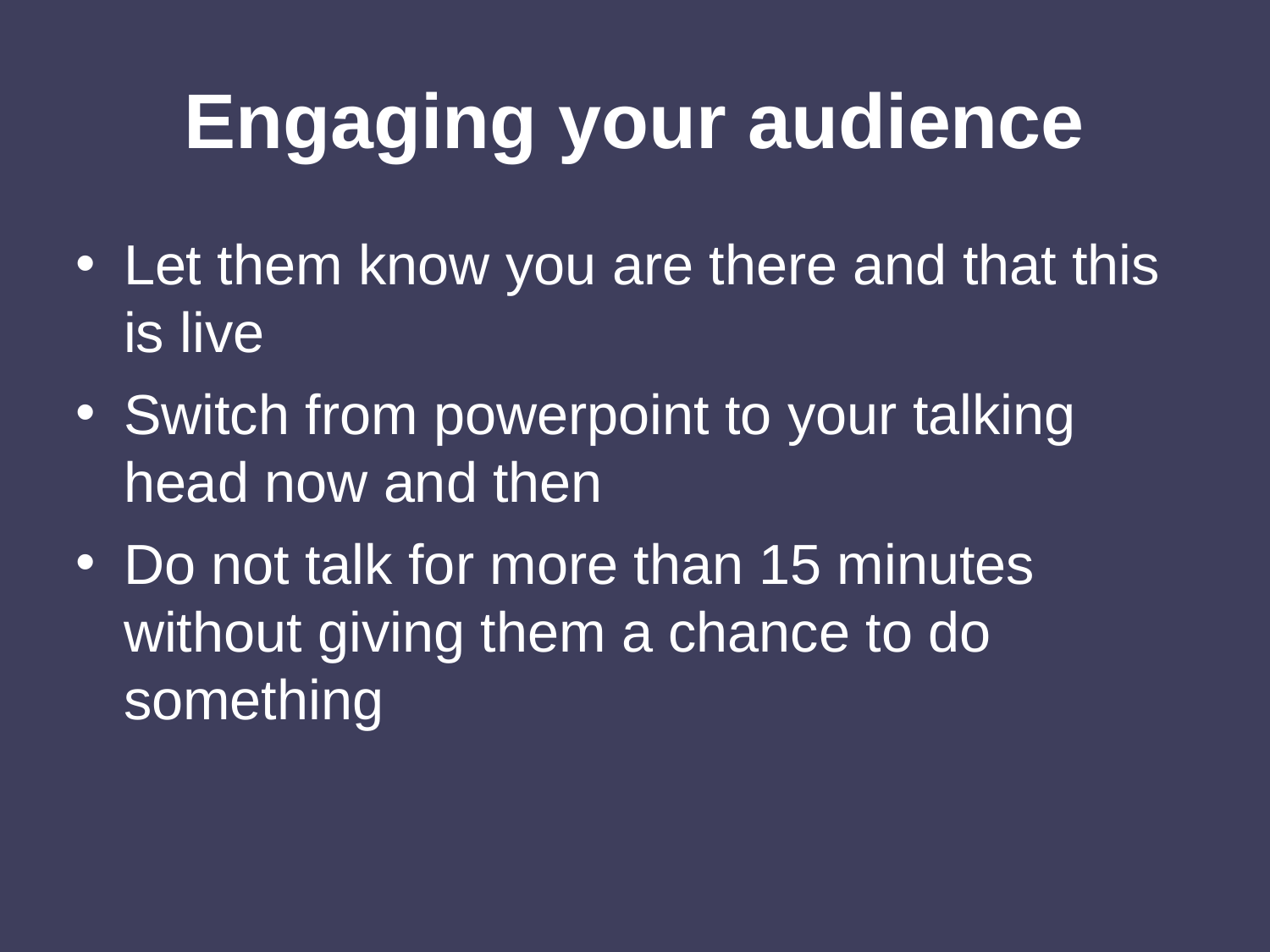

# Engaging your audience
Let them know you are there and that this is live
Switch from powerpoint to your talking head now and then
Do not talk for more than 15 minutes without giving them a chance to do something

## Slide 19
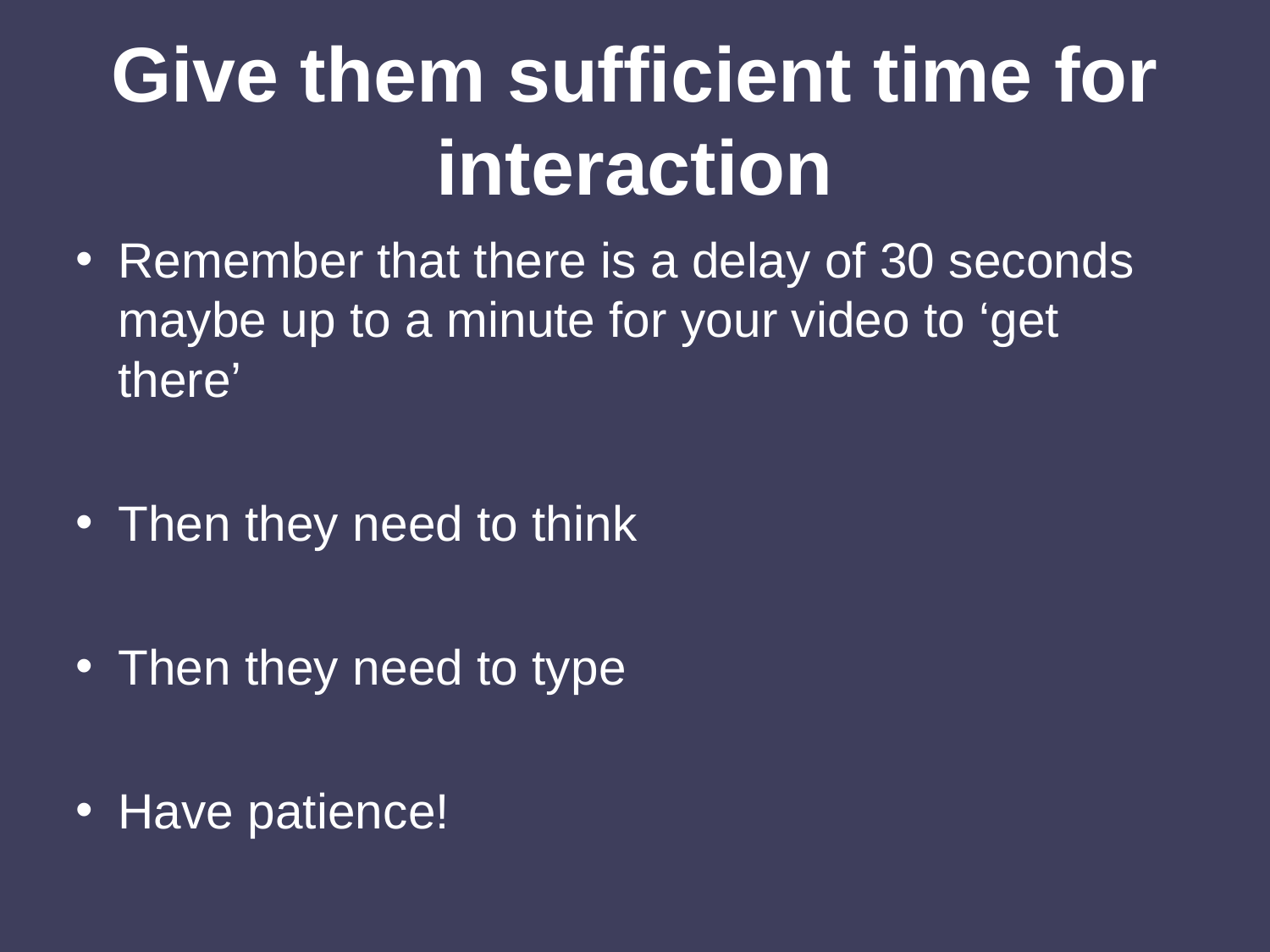

# Give them sufficient time for interaction
Remember that there is a delay of 30 seconds maybe up to a minute for your video to ‘get there’
Then they need to think
Then they need to type
Have patience!

## Slide 20
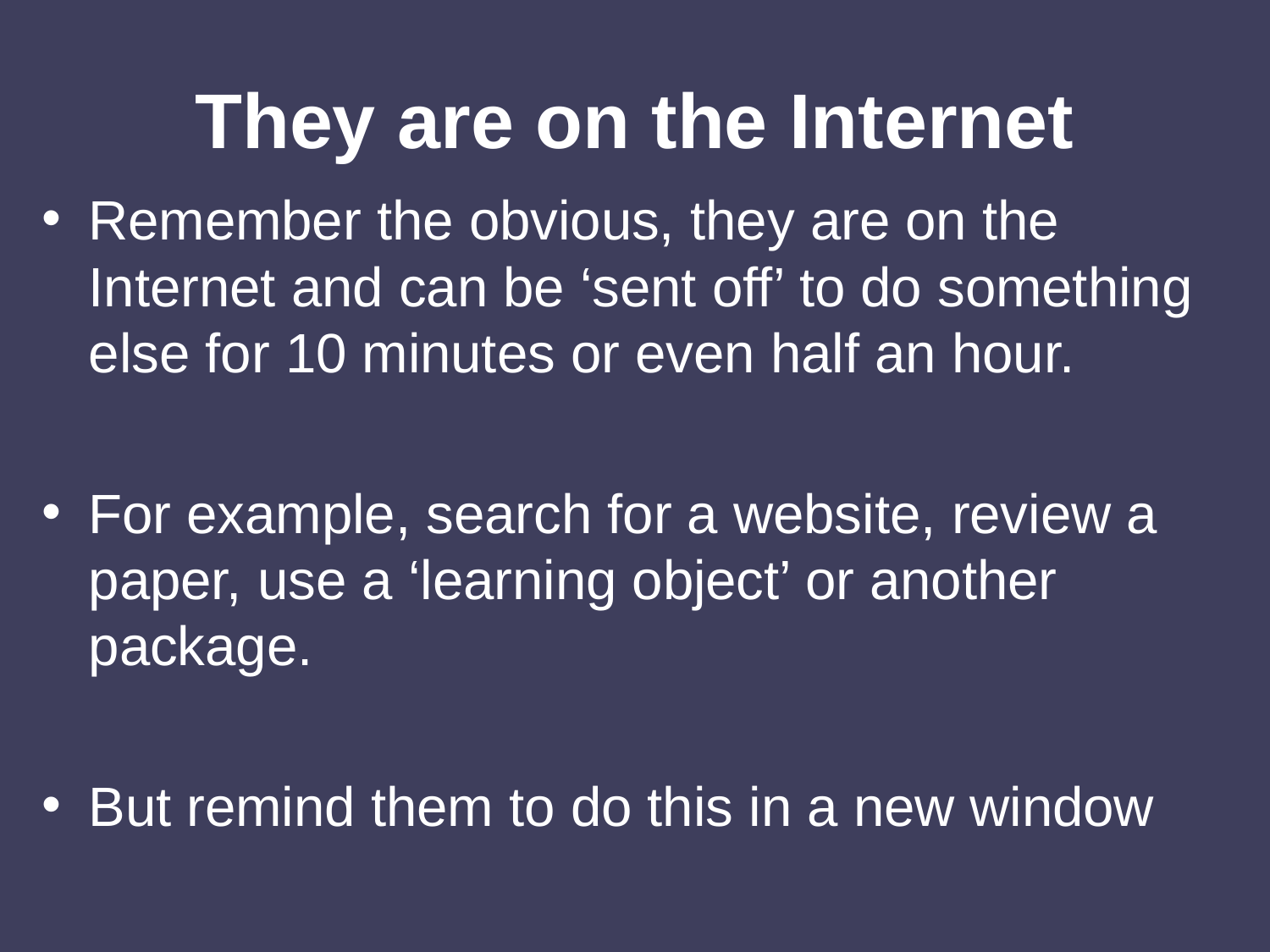

# They are on the Internet
Remember the obvious, they are on the Internet and can be ‘sent off’ to do something else for 10 minutes or even half an hour.
For example, search for a website, review a paper, use a ‘learning object’ or another package.
But remind them to do this in a new window

## Slide 21
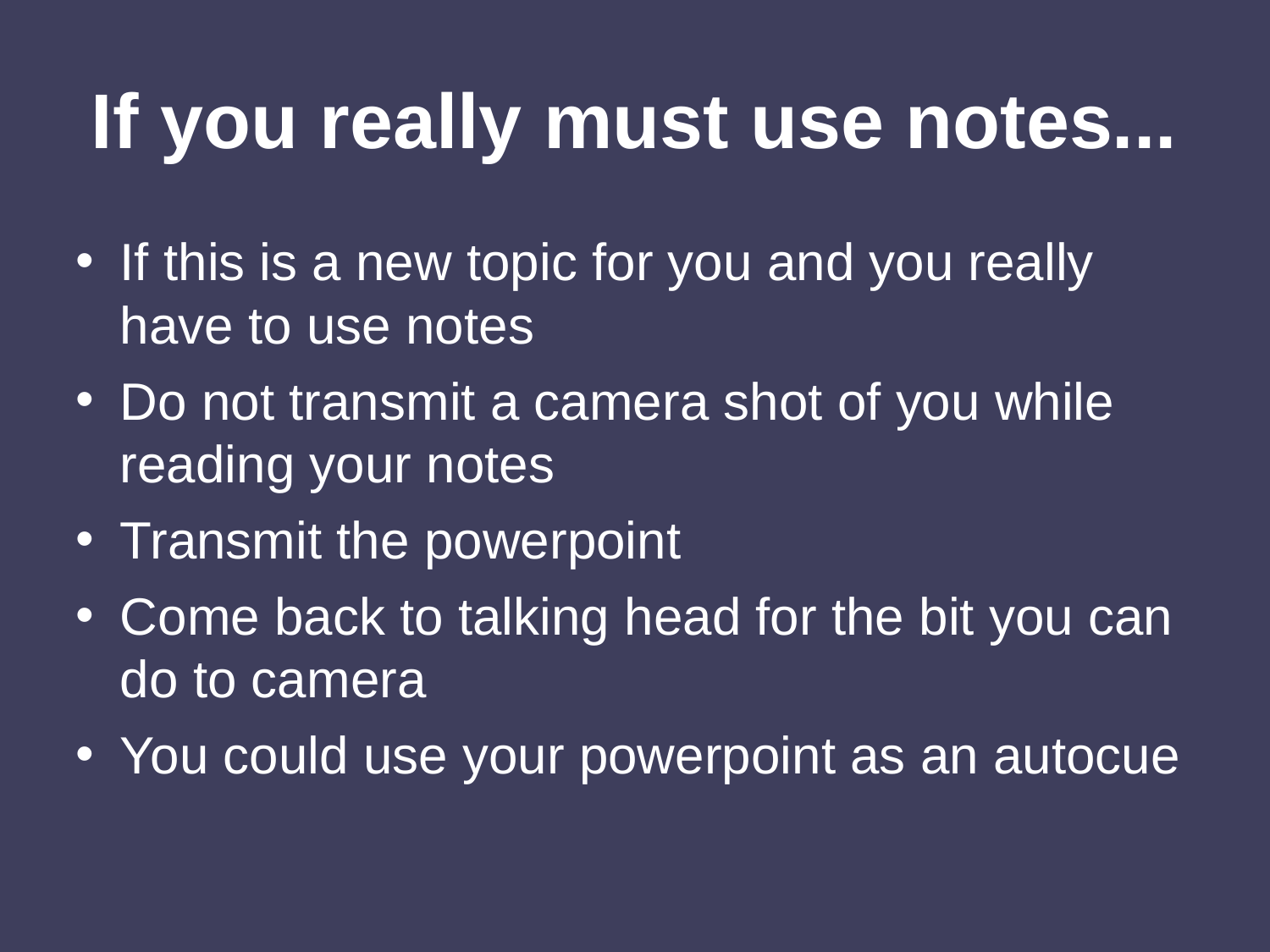

# If you really must use notes...
If this is a new topic for you and you really have to use notes
Do not transmit a camera shot of you while reading your notes
Transmit the powerpoint
Come back to talking head for the bit you can do to camera
You could use your powerpoint as an autocue

## Slide 22
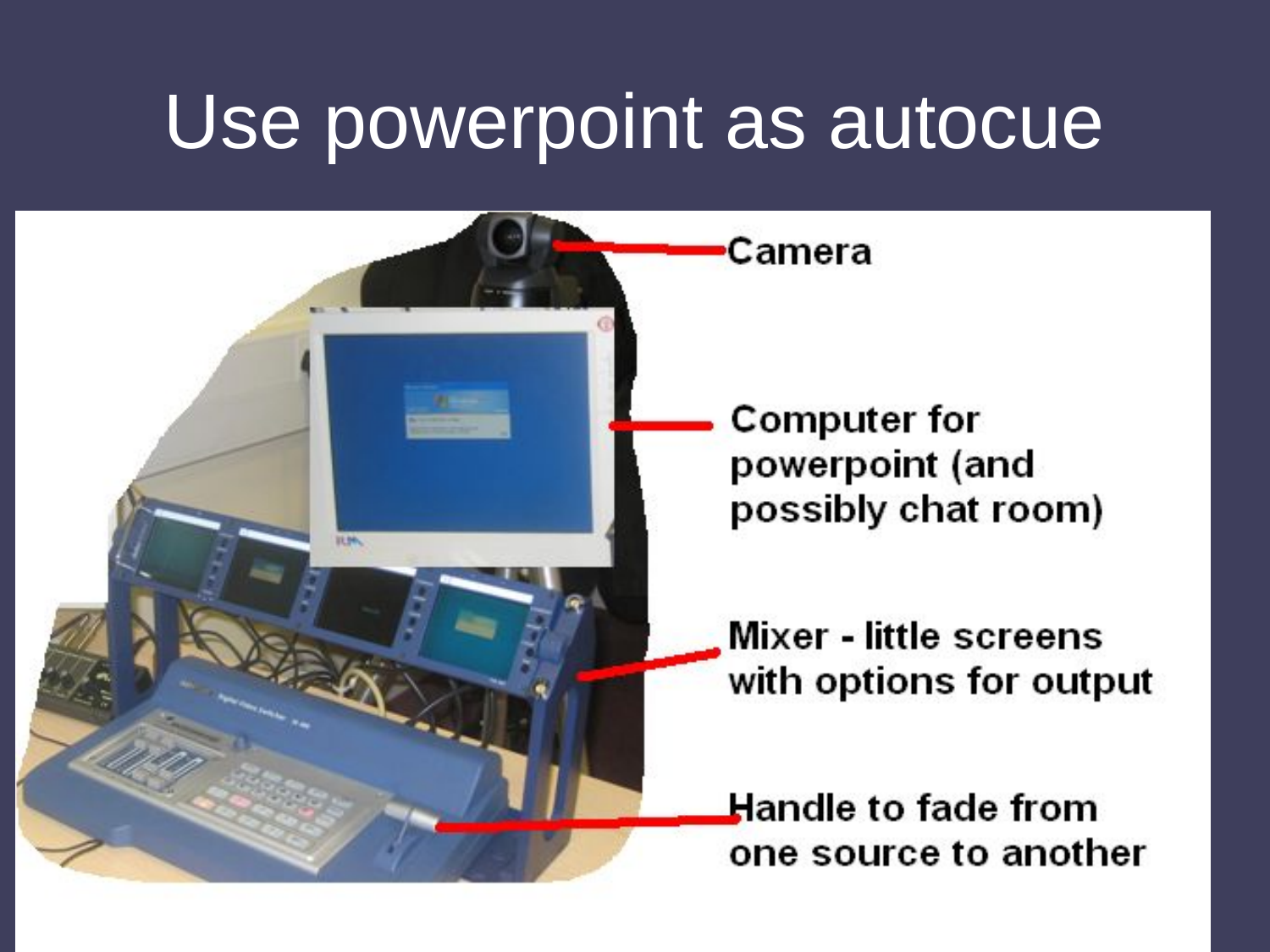

# Use powerpoint as autocue

## Slide 23
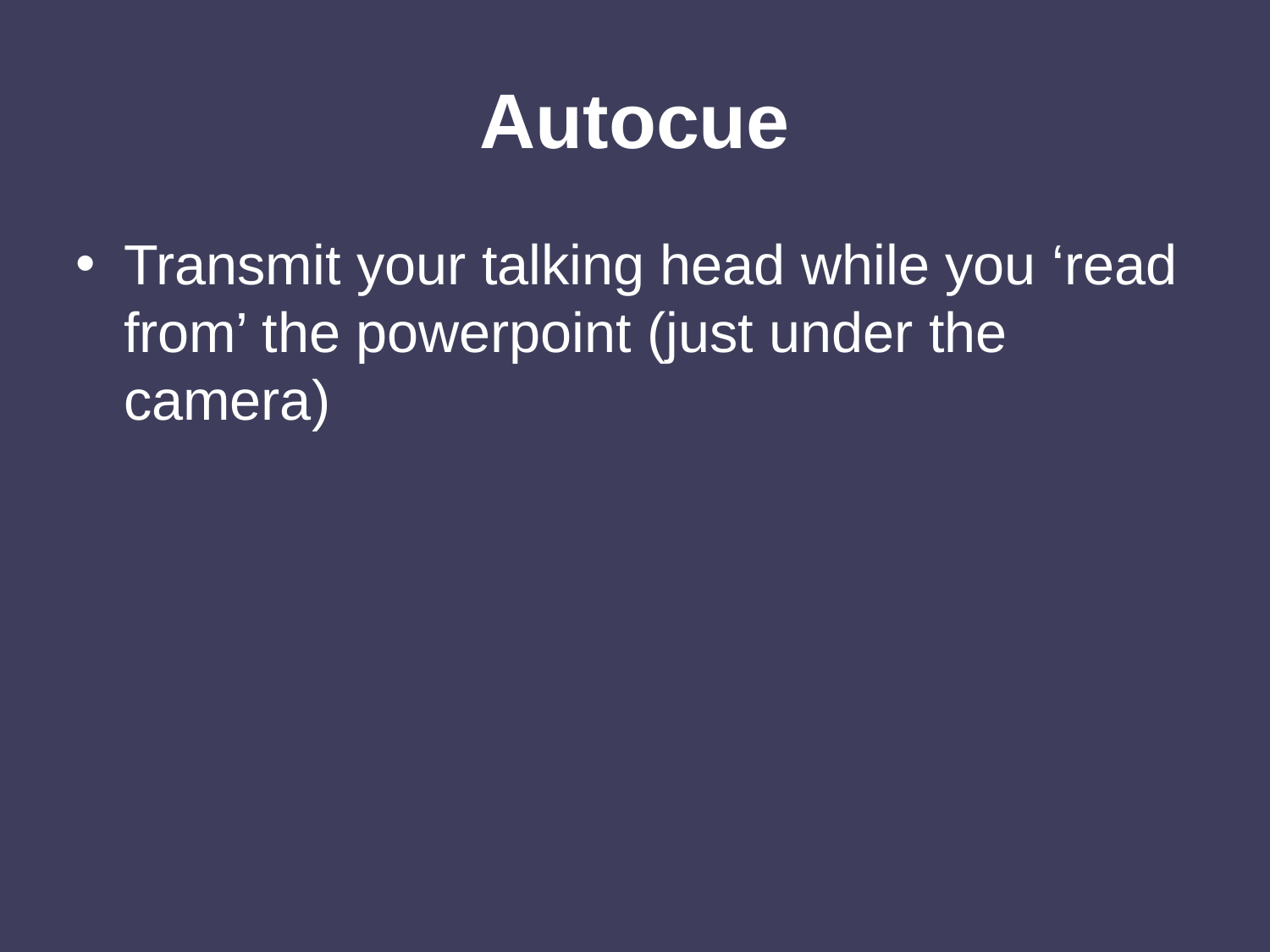

# Autocue
Transmit your talking head while you ‘read from’ the powerpoint (just under the camera)

## Slide 24
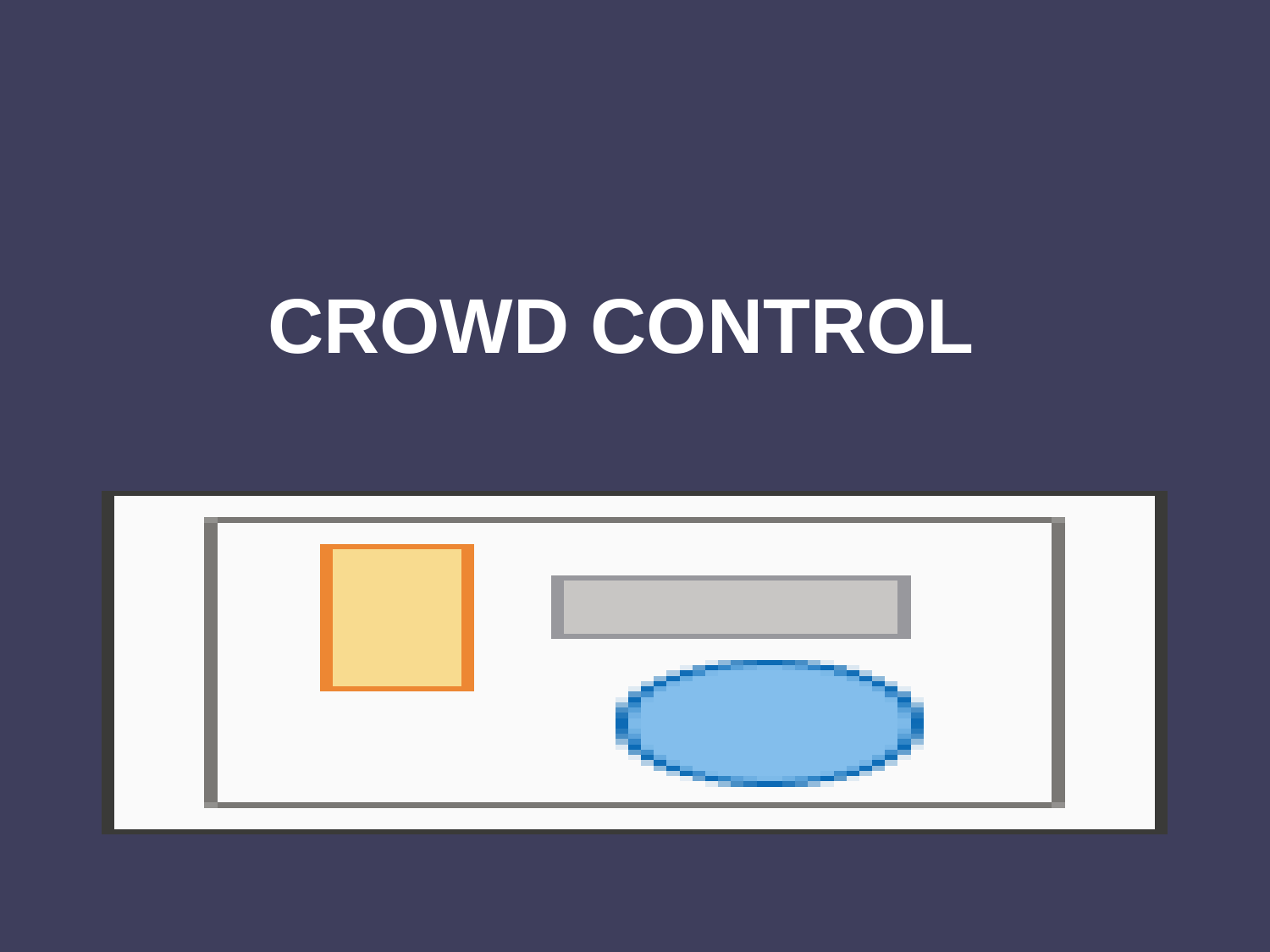

# CROWD CONTROL

## Slide 25
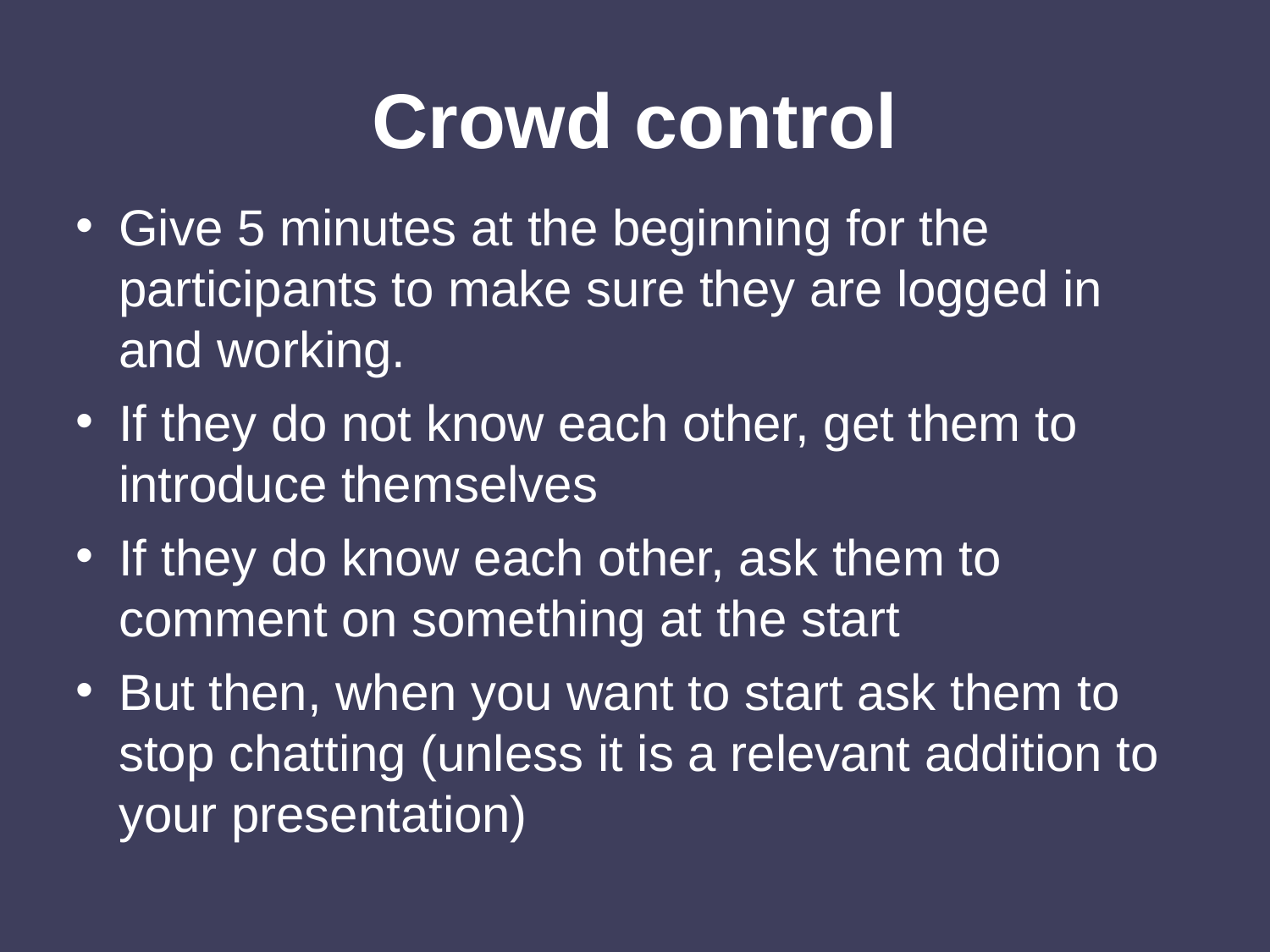

# Crowd control
Give 5 minutes at the beginning for the participants to make sure they are logged in and working.
If they do not know each other, get them to introduce themselves
If they do know each other, ask them to comment on something at the start
But then, when you want to start ask them to stop chatting (unless it is a relevant addition to your presentation)

## Slide 26
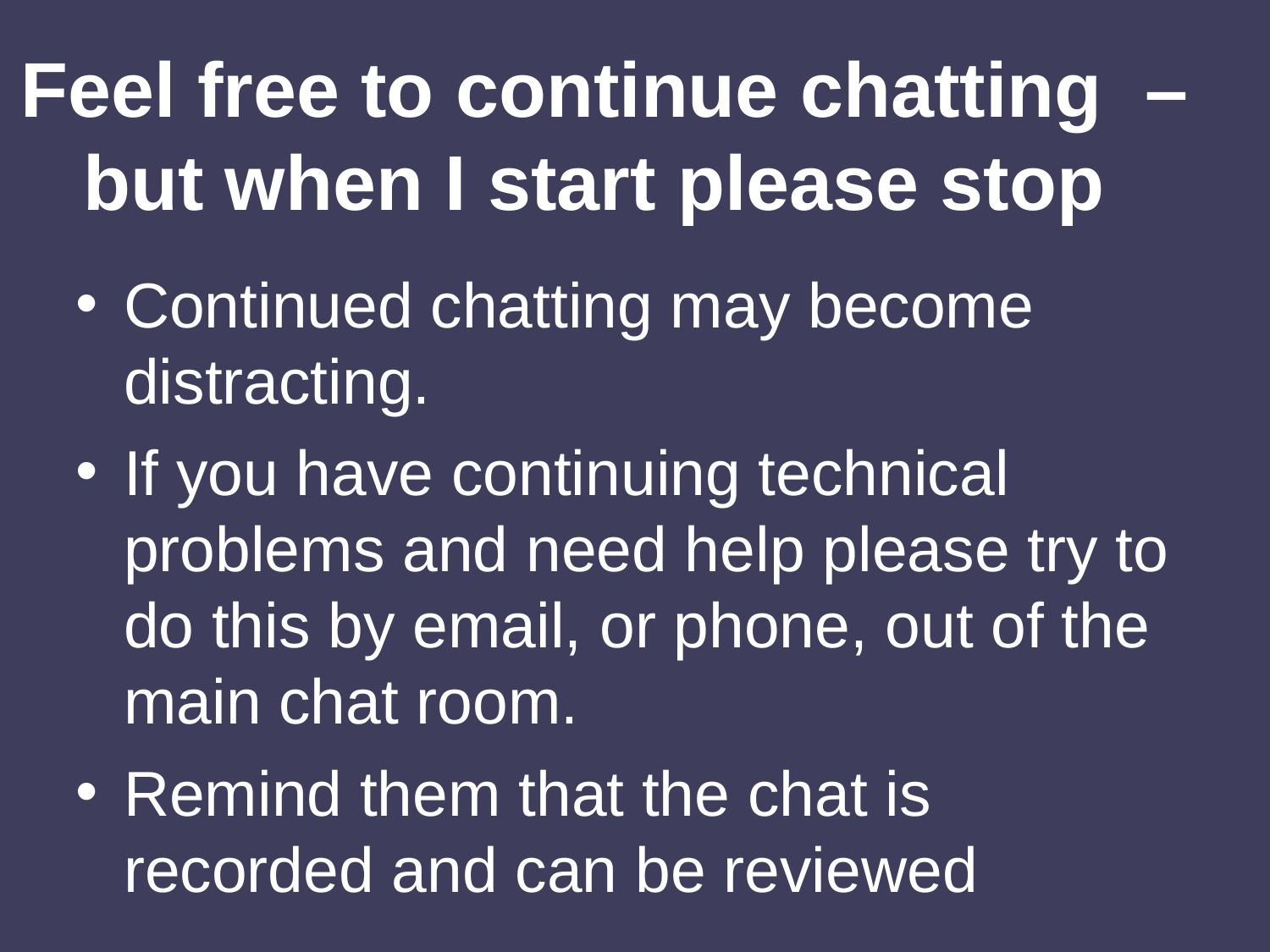

# Feel free to continue chatting – but when I start please stop
Continued chatting may become distracting.
If you have continuing technical problems and need help please try to do this by email, or phone, out of the main chat room.
Remind them that the chat is recorded and can be reviewed

## Slide 27
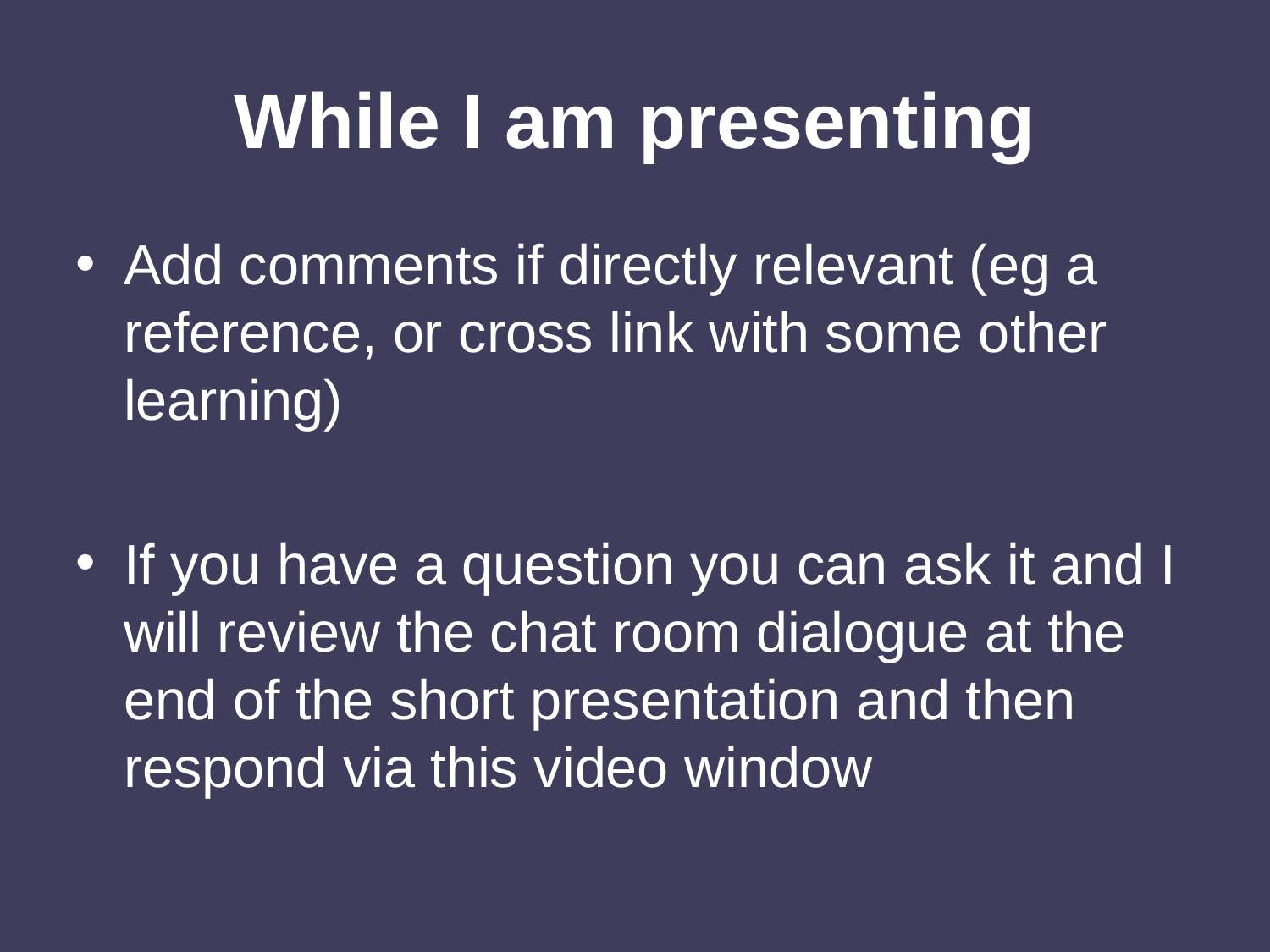

# While I am presenting
Add comments if directly relevant (eg a reference, or cross link with some other learning)
If you have a question you can ask it and I will review the chat room dialogue at the end of the short presentation and then respond via this video window

## Slide 28
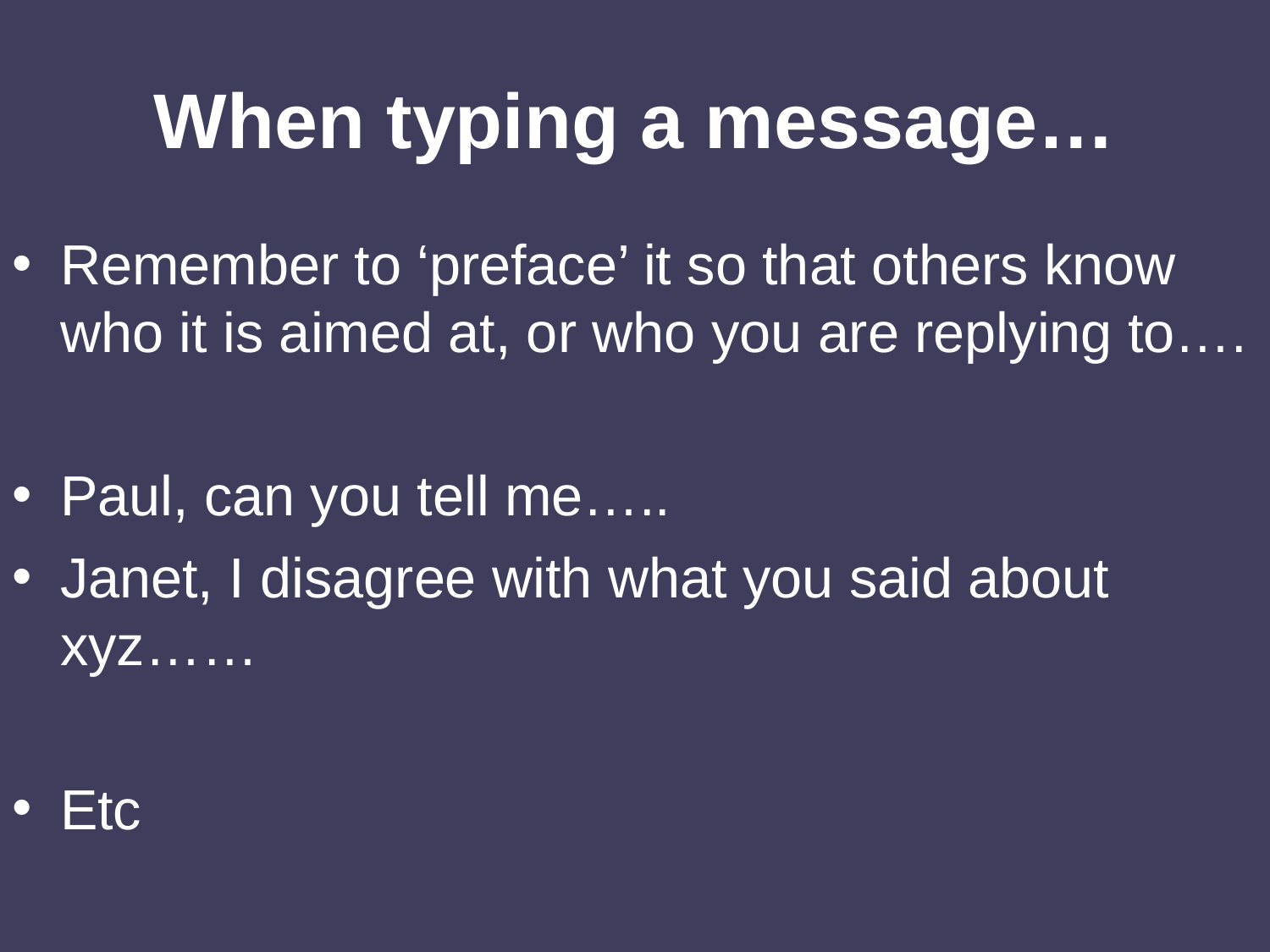

# When typing a message…
Remember to ‘preface’ it so that others know who it is aimed at, or who you are replying to….
Paul, can you tell me…..
Janet, I disagree with what you said about xyz……
Etc

## Slide 29
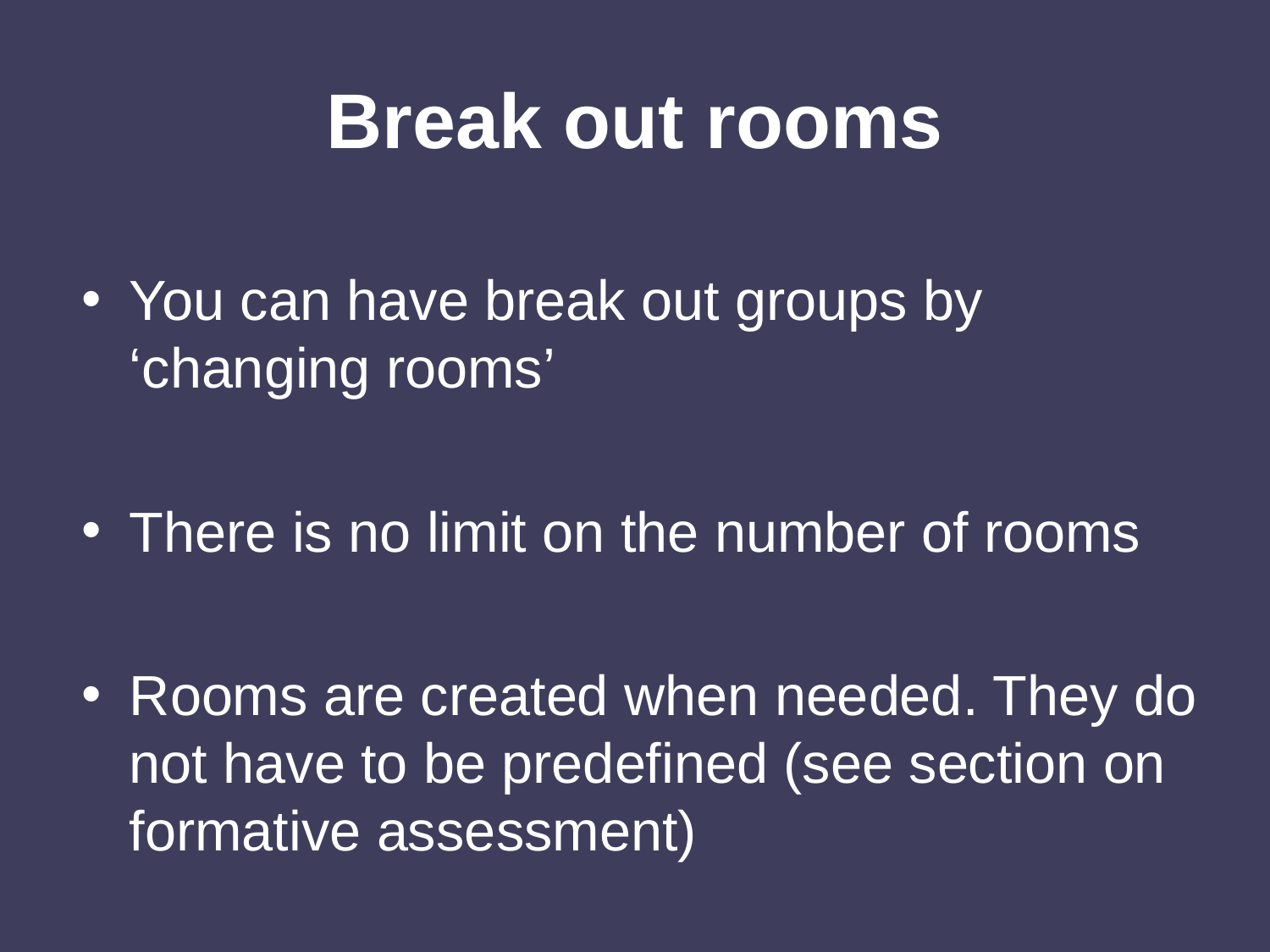

# Break out rooms
You can have break out groups by ‘changing rooms’
There is no limit on the number of rooms
Rooms are created when needed. They do not have to be predefined (see section on formative assessment)

## Slide 30
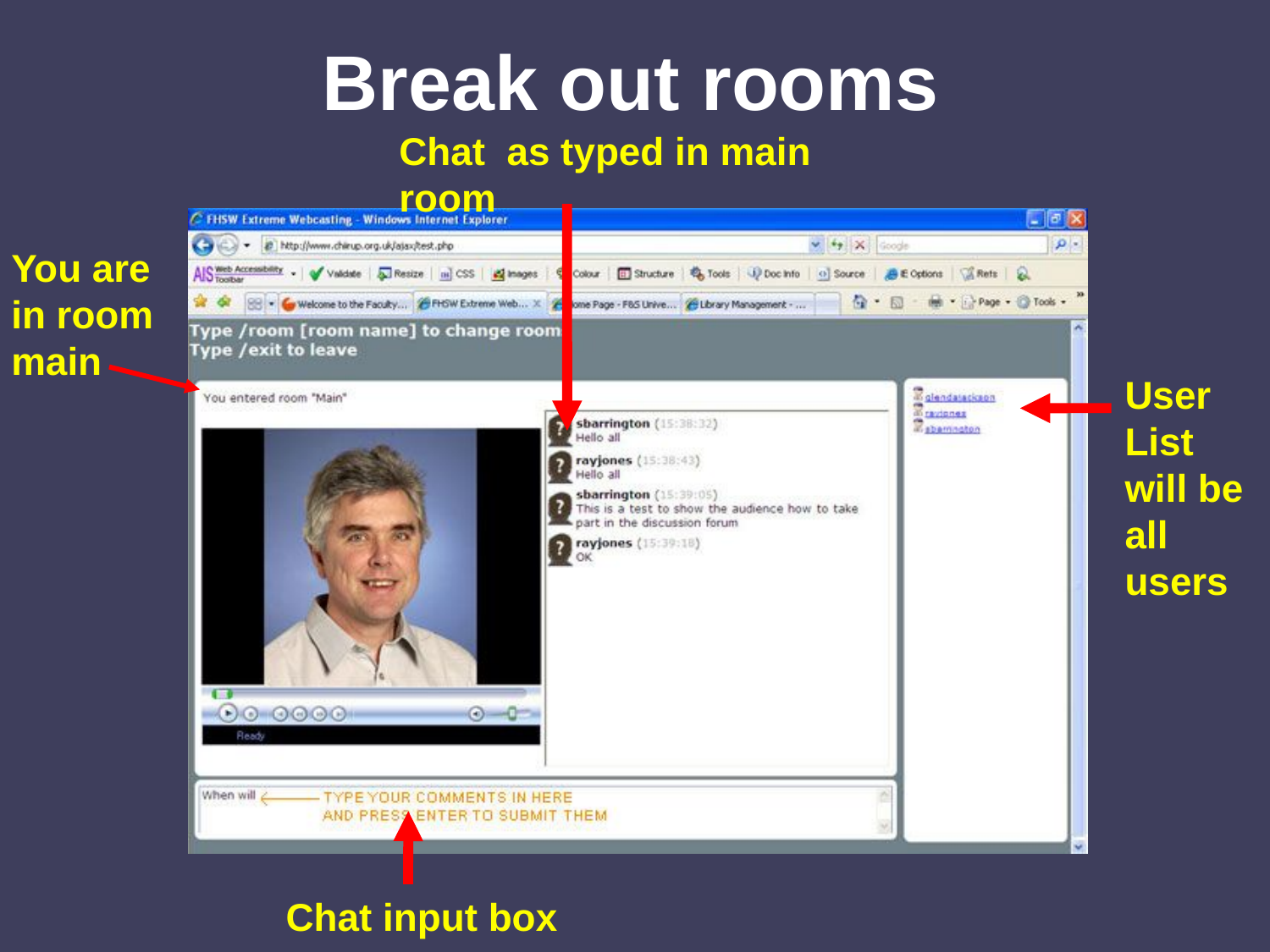

# Break out rooms
Chat as typed in main room
You are in room main
User List will be all users
Chat input box

## Slide 31
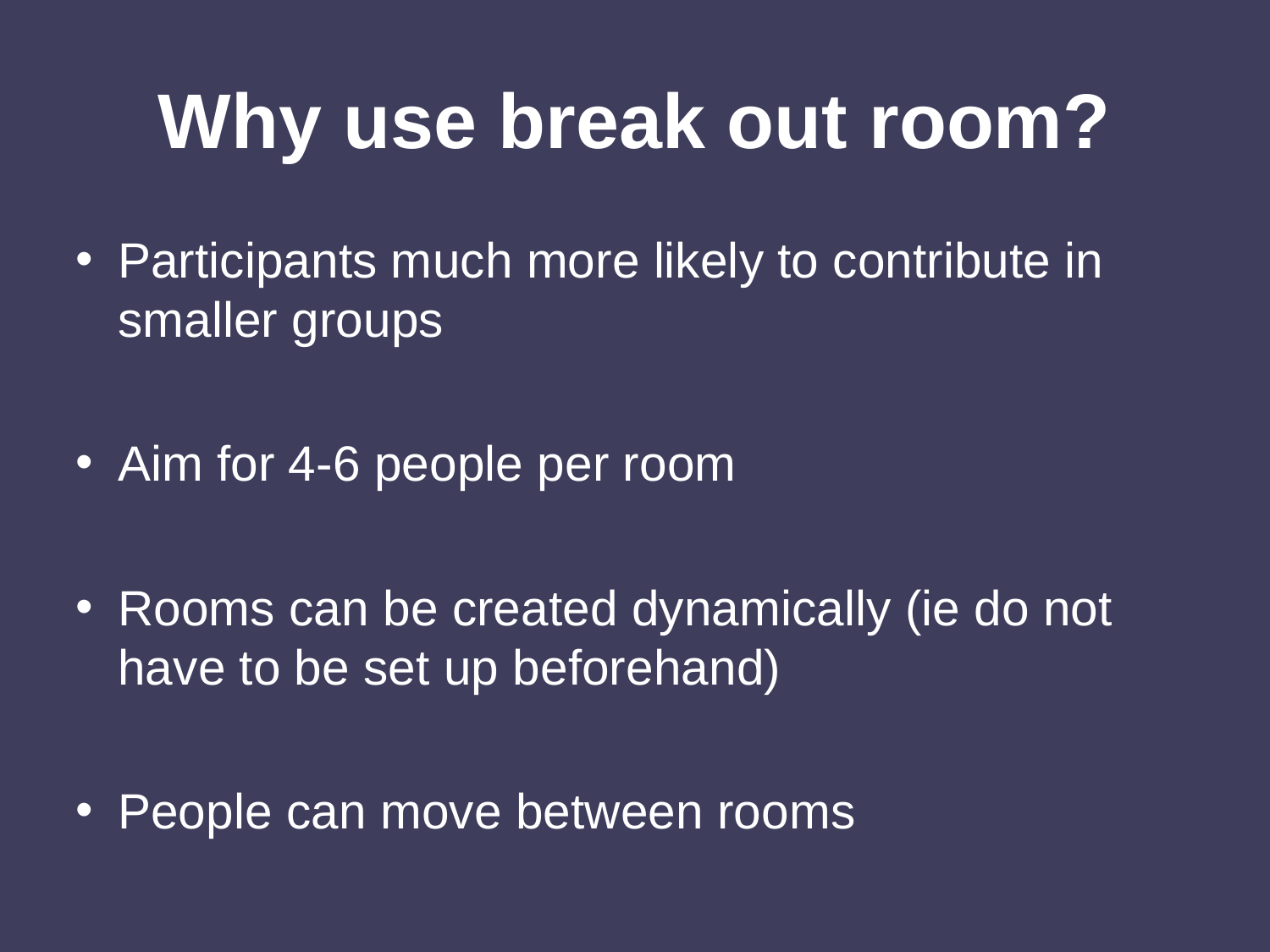

# Why use break out room?
Participants much more likely to contribute in smaller groups
Aim for 4-6 people per room
Rooms can be created dynamically (ie do not have to be set up beforehand)
People can move between rooms

## Slide 32
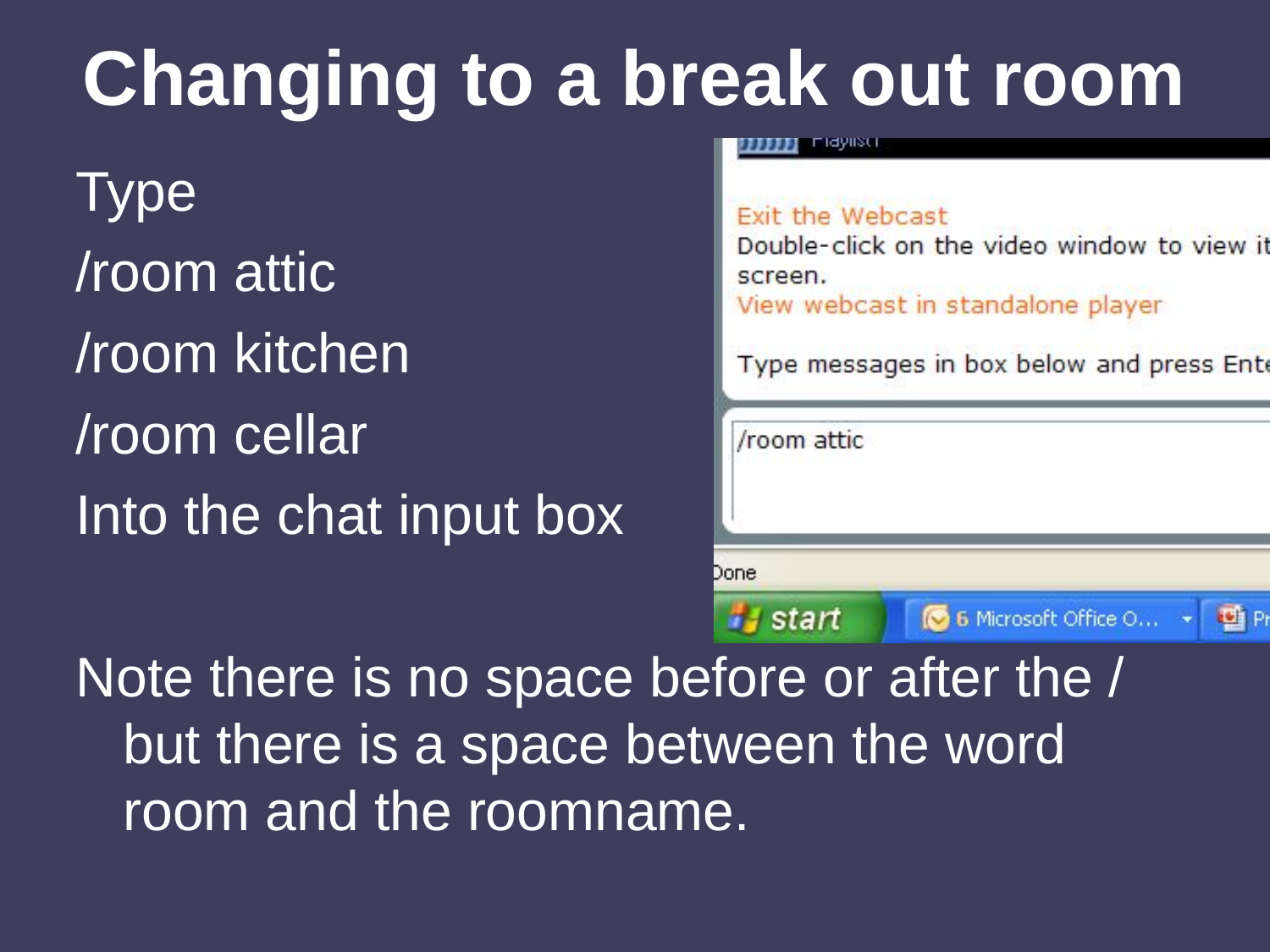

# Changing to a break out room
Type
/room attic
/room kitchen
/room cellar
Into the chat input box
Note there is no space before or after the / but there is a space between the word room and the roomname.

## Slide 33
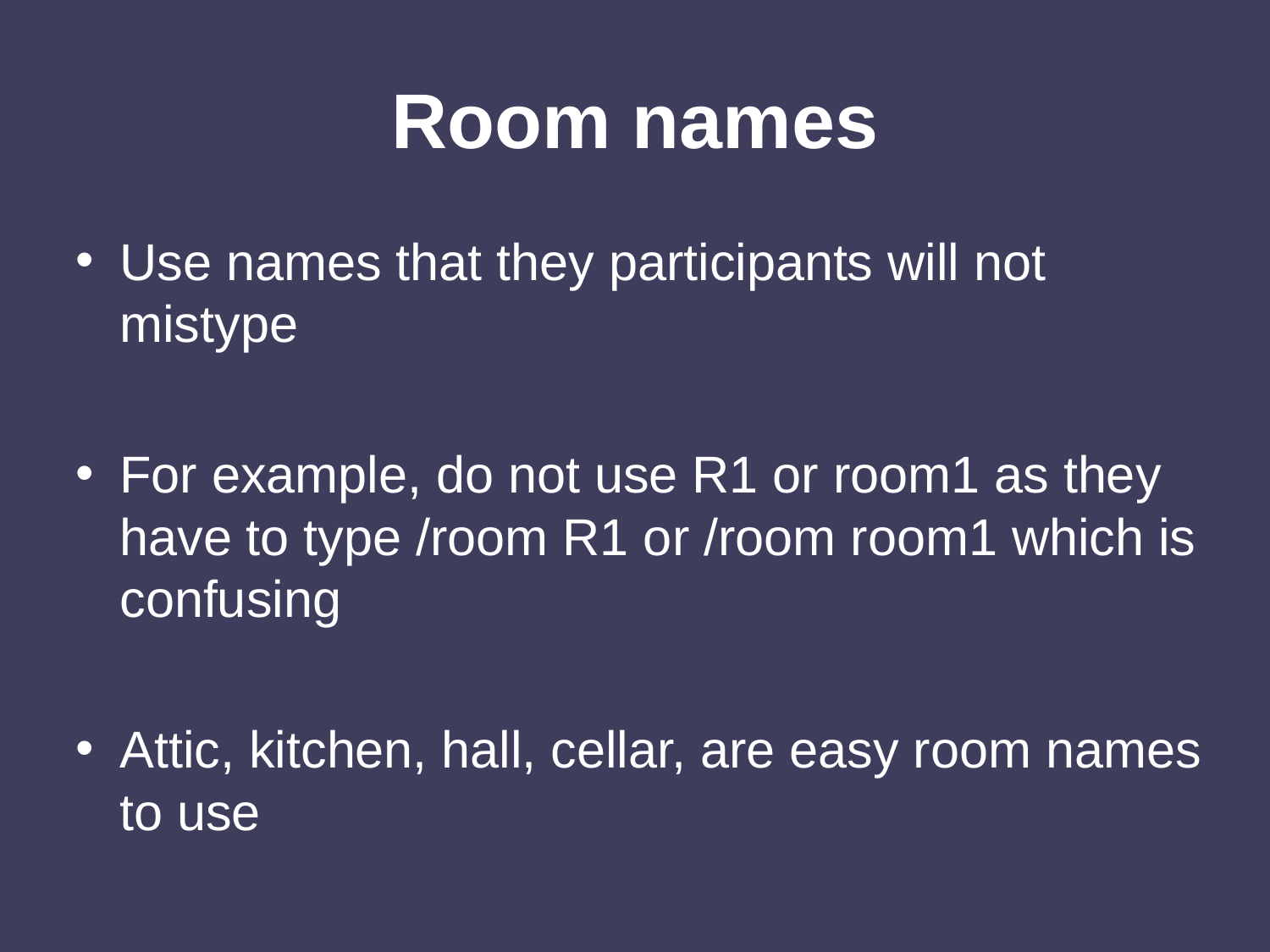

# Room names
Use names that they participants will not mistype
For example, do not use R1 or room1 as they have to type /room R1 or /room room1 which is confusing
Attic, kitchen, hall, cellar, are easy room names to use

## Slide 34
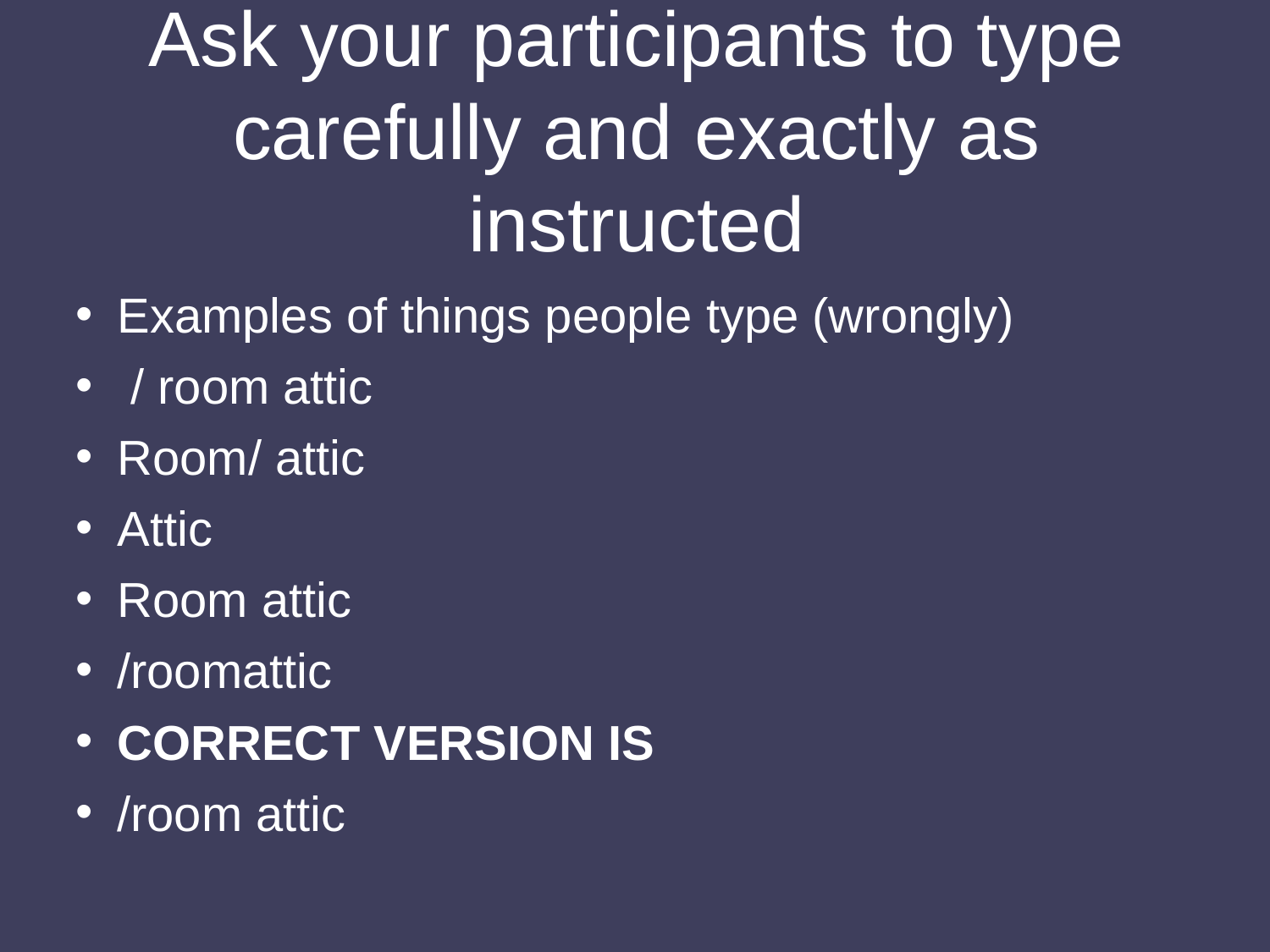

# Ask your participants to type carefully and exactly as instructed
Examples of things people type (wrongly)
 / room attic
Room/ attic
Attic
Room attic
/roomattic
CORRECT VERSION IS
/room attic

## Slide 35
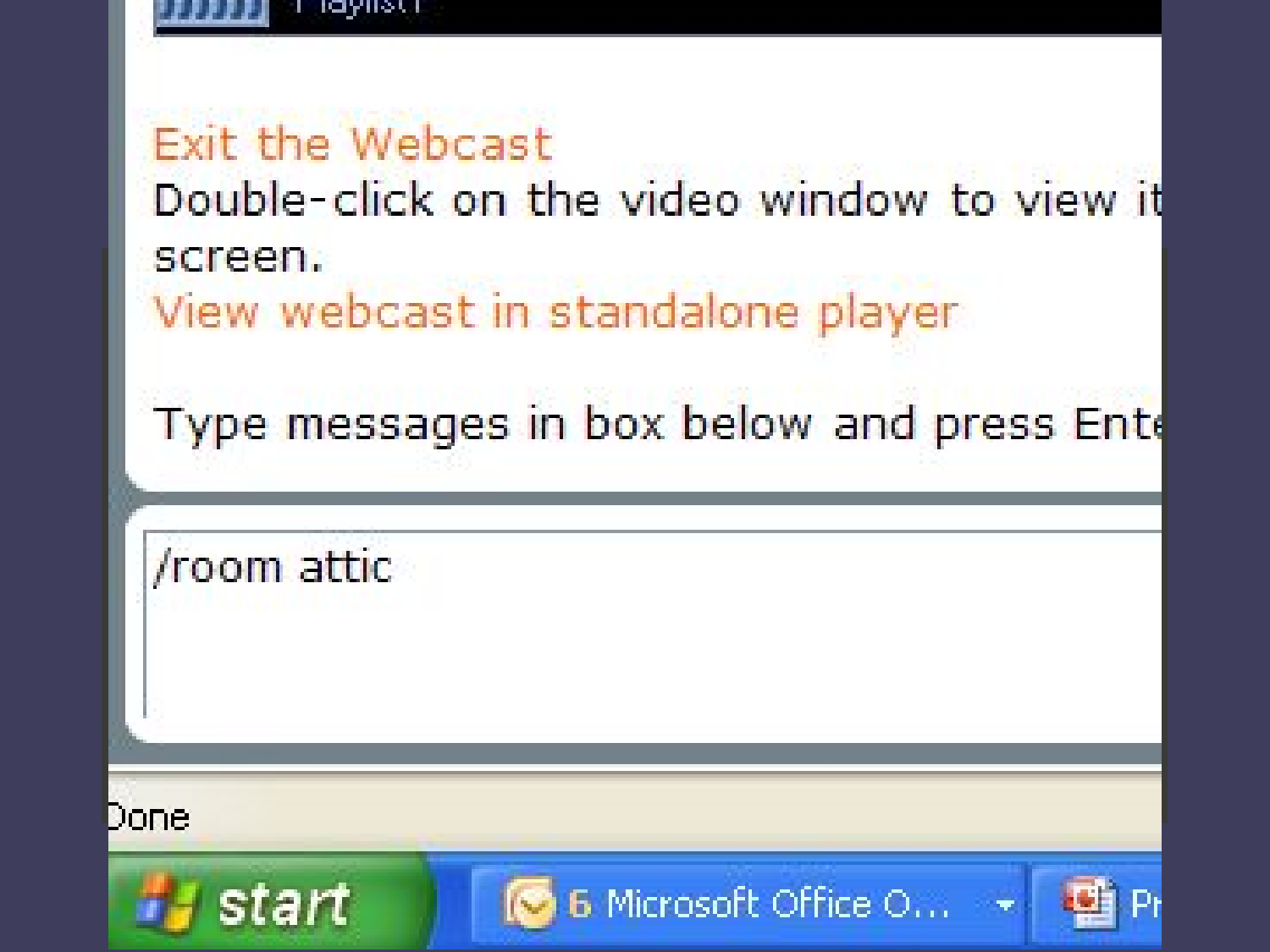

#

## Slide 36
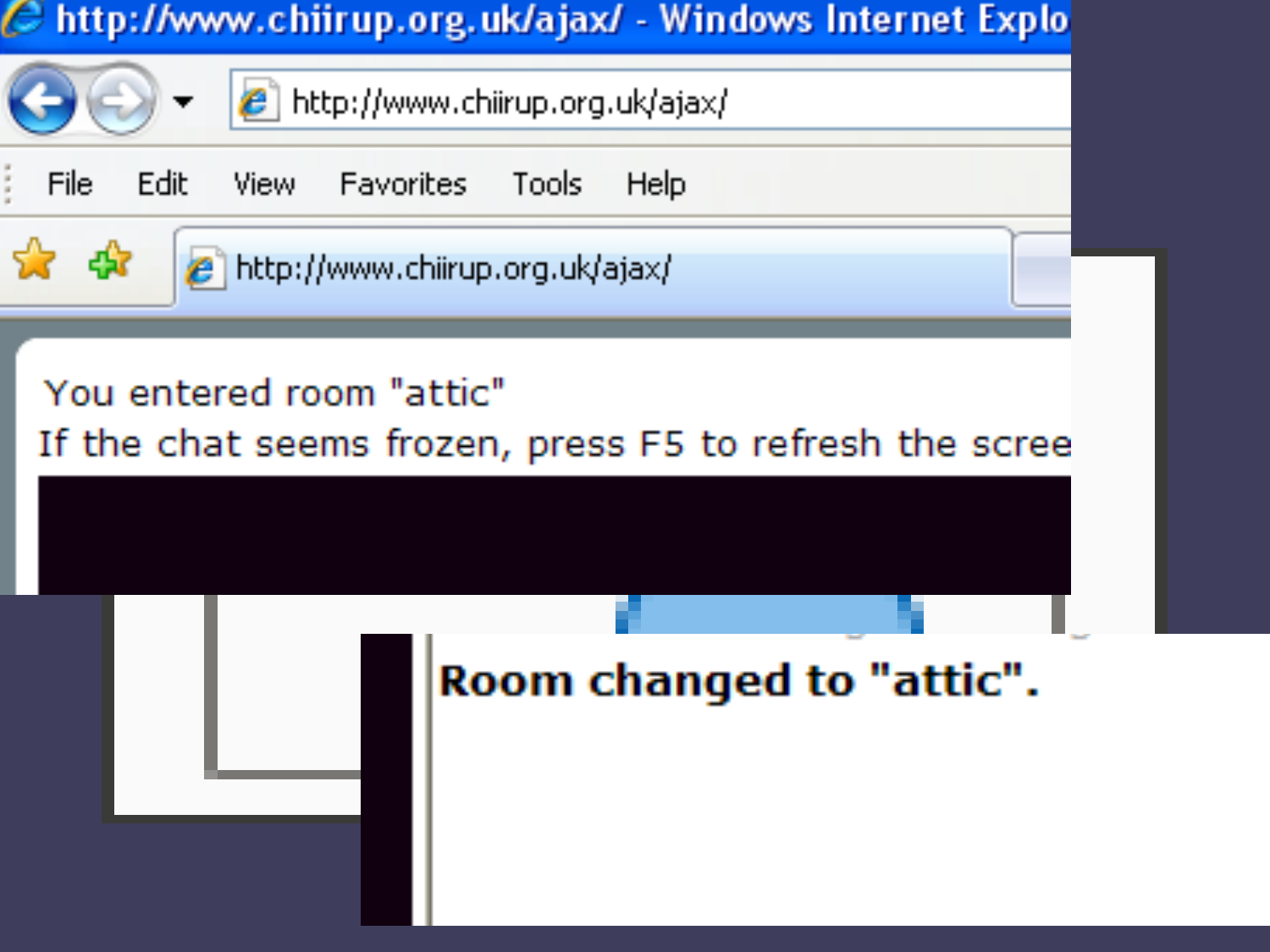

#

## Slide 37
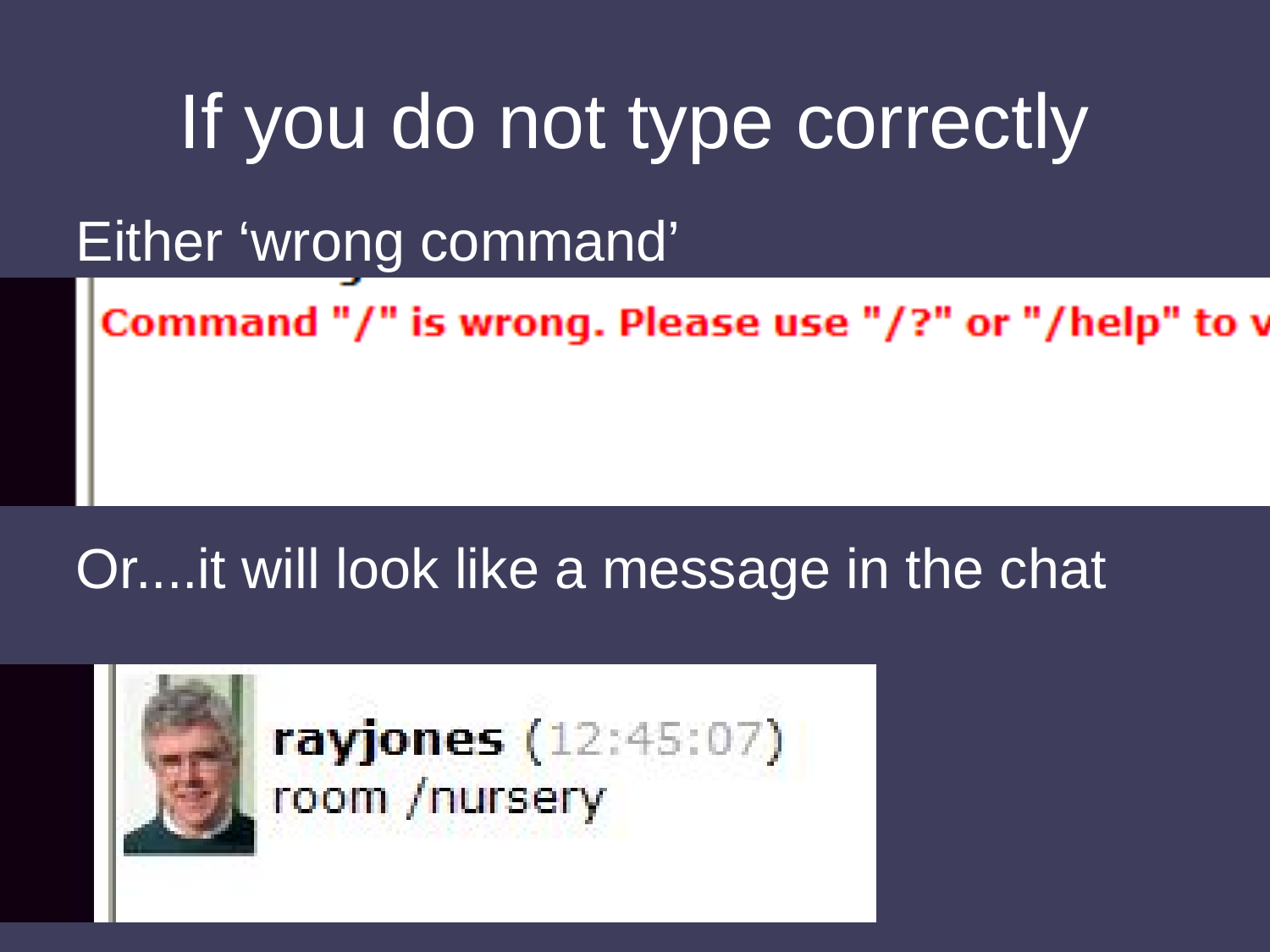

# If you do not type correctly
Either ‘wrong command’
Or....it will look like a message in the chat

## Slide 38
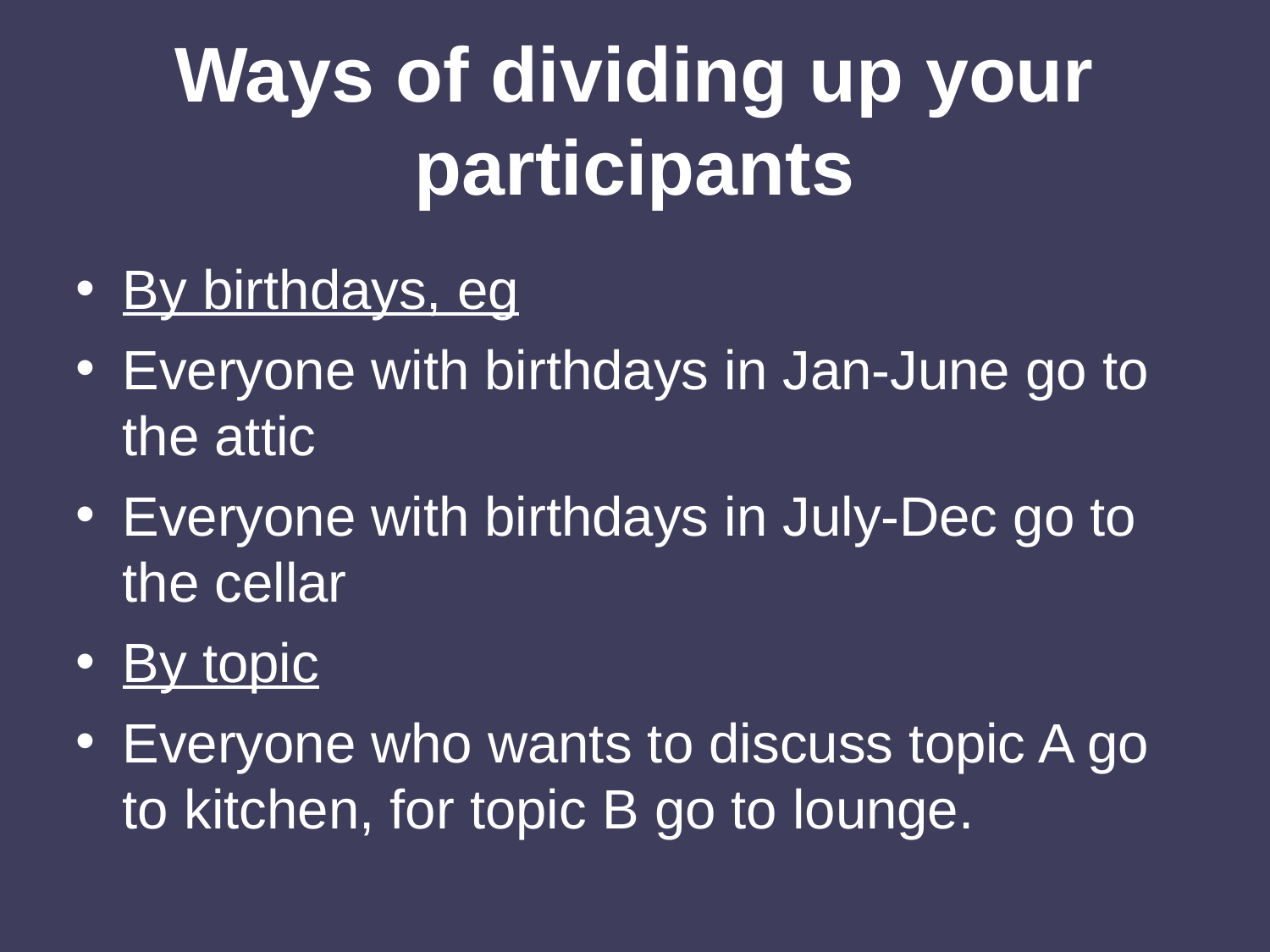

# Ways of dividing up your participants
By birthdays, eg
Everyone with birthdays in Jan-June go to the attic
Everyone with birthdays in July-Dec go to the cellar
By topic
Everyone who wants to discuss topic A go to kitchen, for topic B go to lounge.

## Slide 39
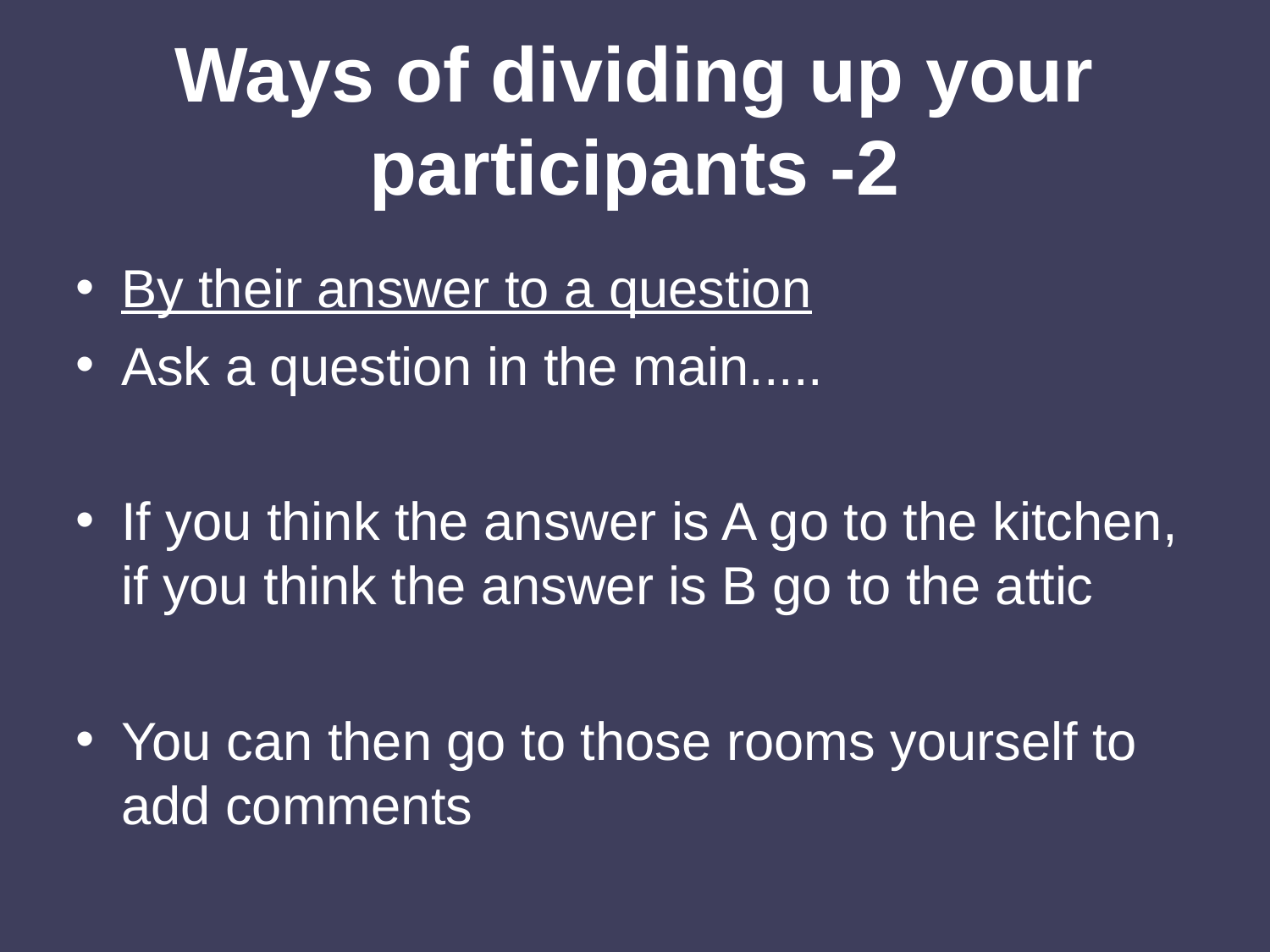

# Ways of dividing up your participants -2
By their answer to a question
Ask a question in the main.....
If you think the answer is A go to the kitchen, if you think the answer is B go to the attic
You can then go to those rooms yourself to add comments

## Slide 40
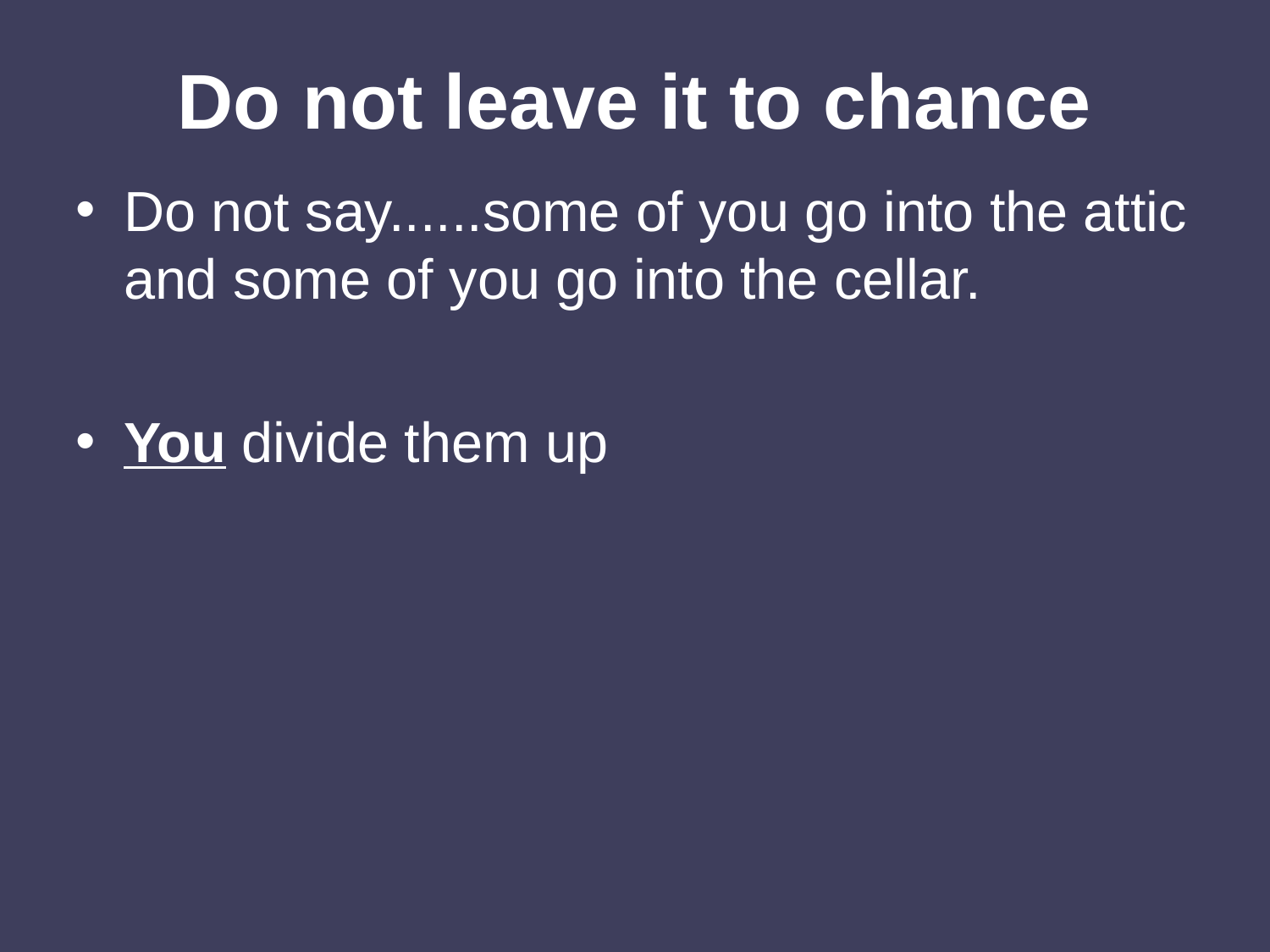

# Do not leave it to chance
Do not say......some of you go into the attic and some of you go into the cellar.
You divide them up

## Slide 41
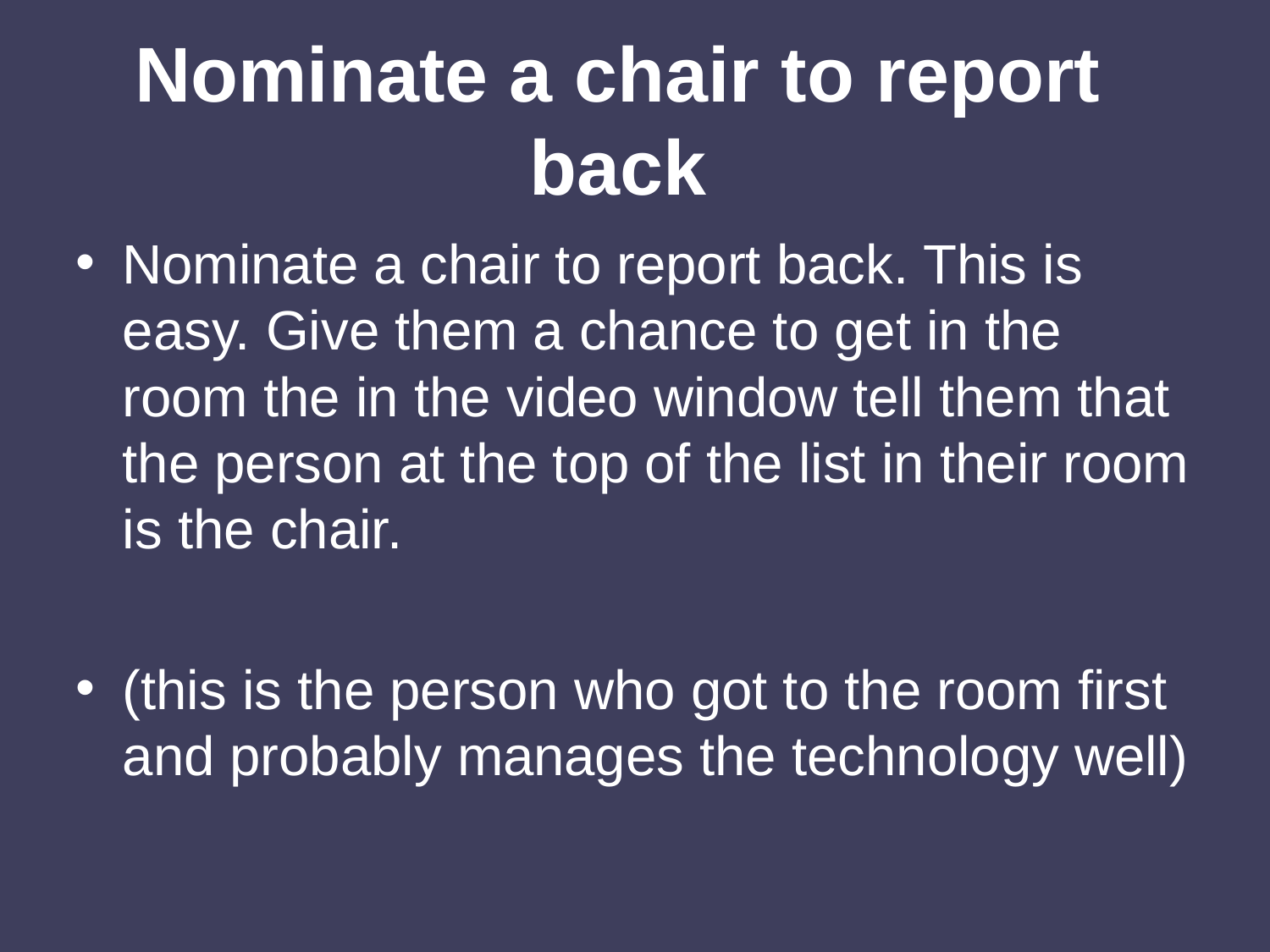

# Nominate a chair to report back
Nominate a chair to report back. This is easy. Give them a chance to get in the room the in the video window tell them that the person at the top of the list in their room is the chair.
(this is the person who got to the room first and probably manages the technology well)

## Slide 42
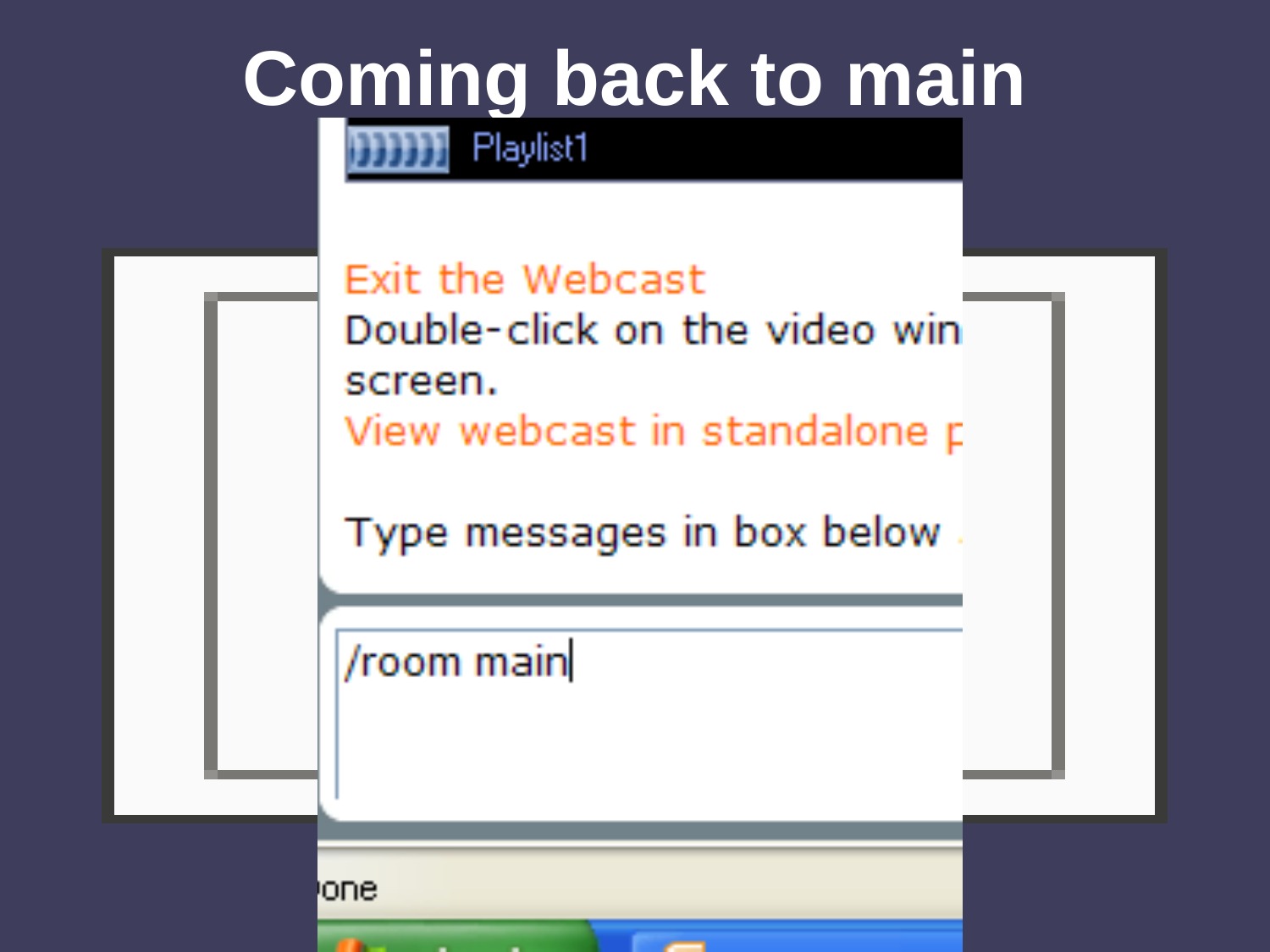

# Coming back to main

## Slide 43
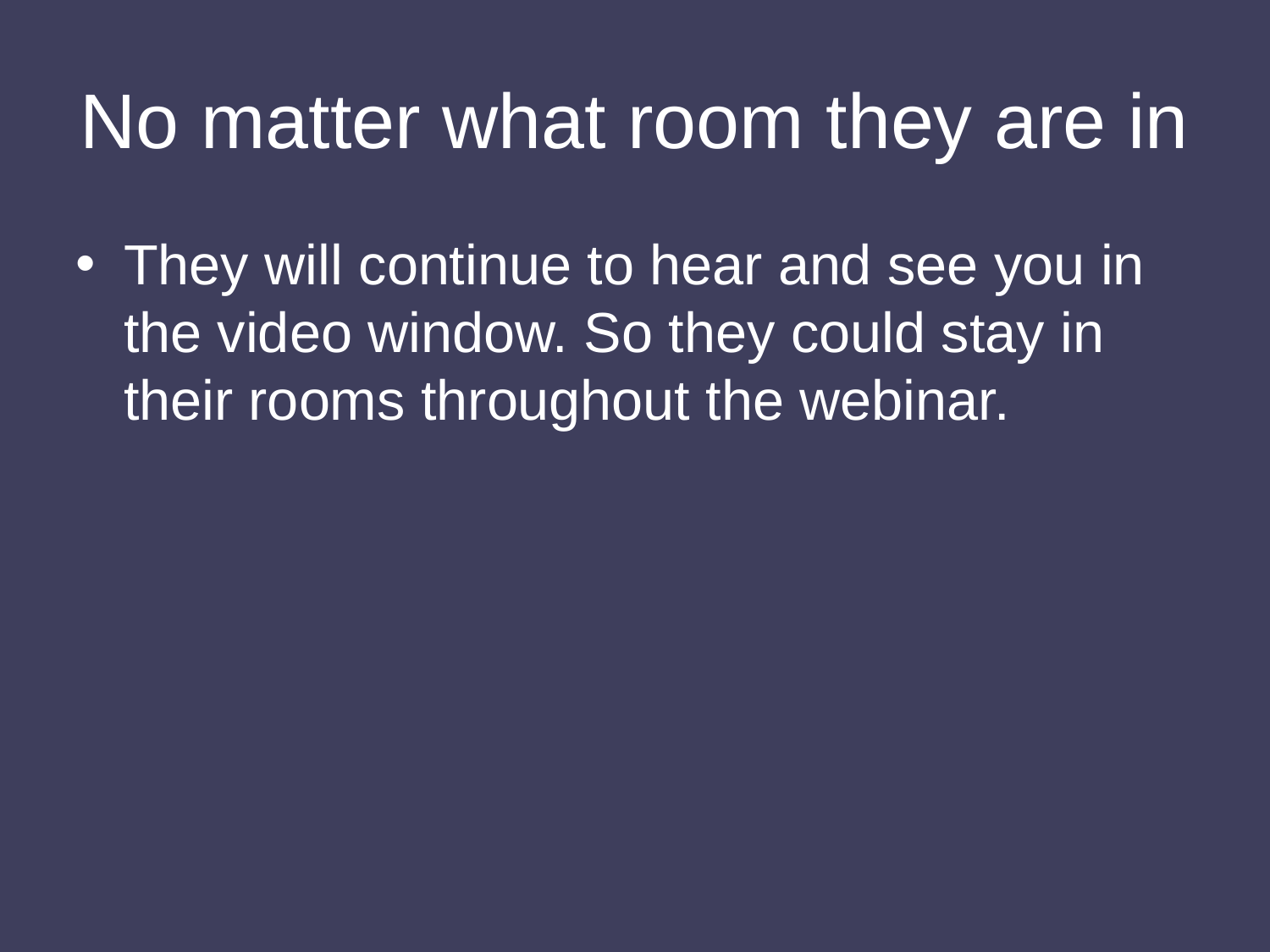

# No matter what room they are in
They will continue to hear and see you in the video window. So they could stay in their rooms throughout the webinar.

## Slide 44
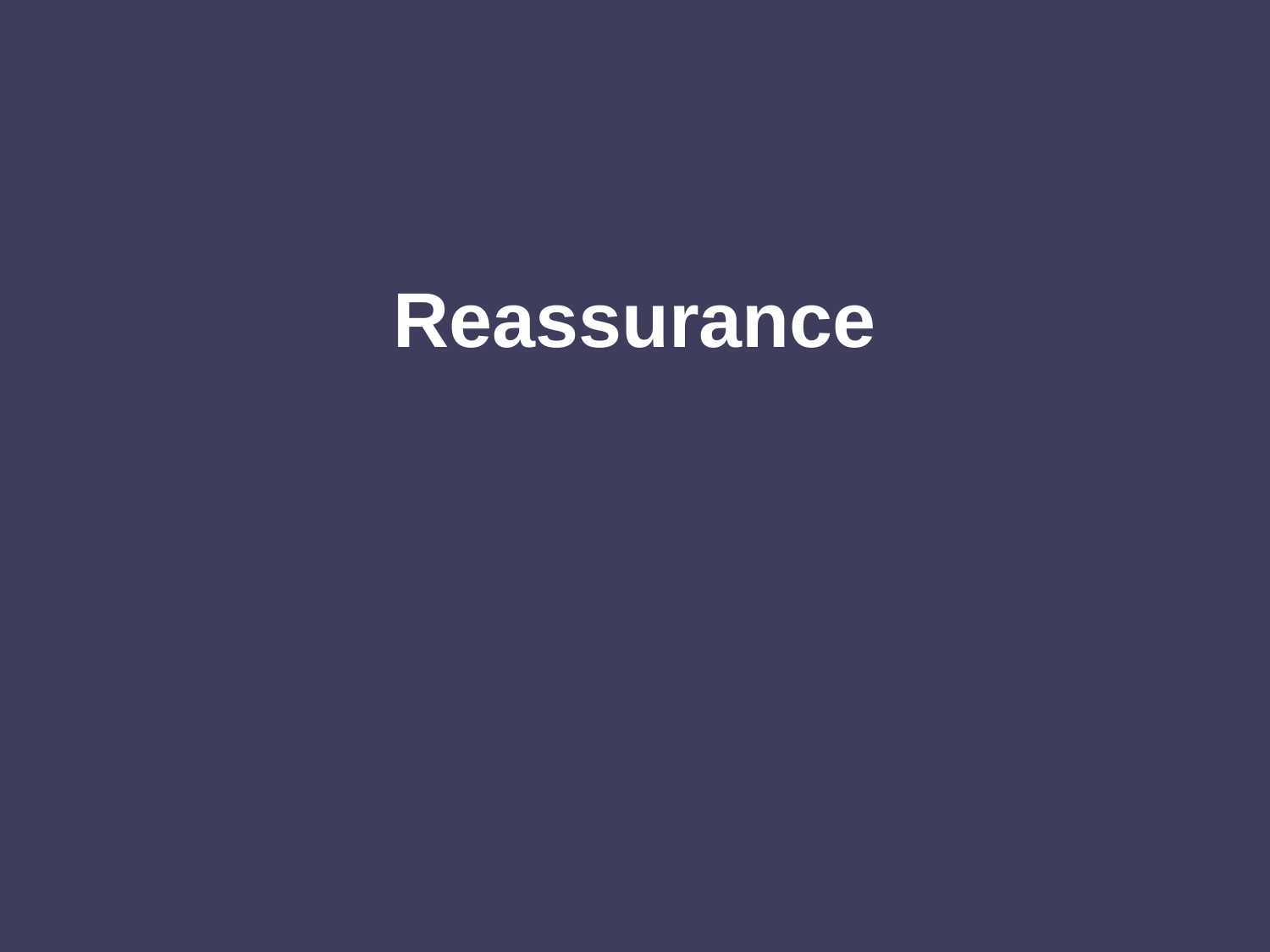

# Reassurance

## Slide 45
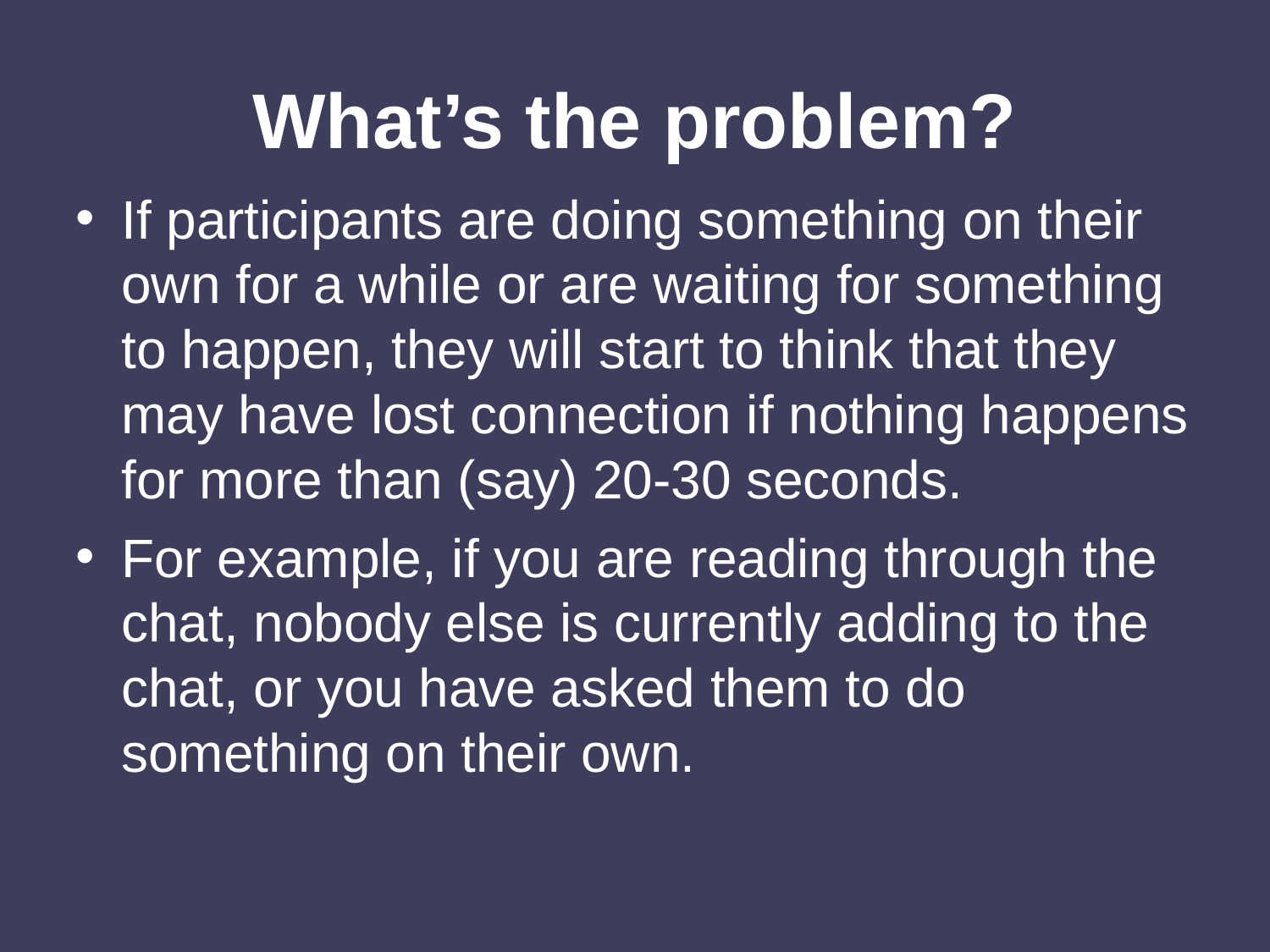

# What’s the problem?
If participants are doing something on their own for a while or are waiting for something to happen, they will start to think that they may have lost connection if nothing happens for more than (say) 20-30 seconds.
For example, if you are reading through the chat, nobody else is currently adding to the chat, or you have asked them to do something on their own.

## Slide 46
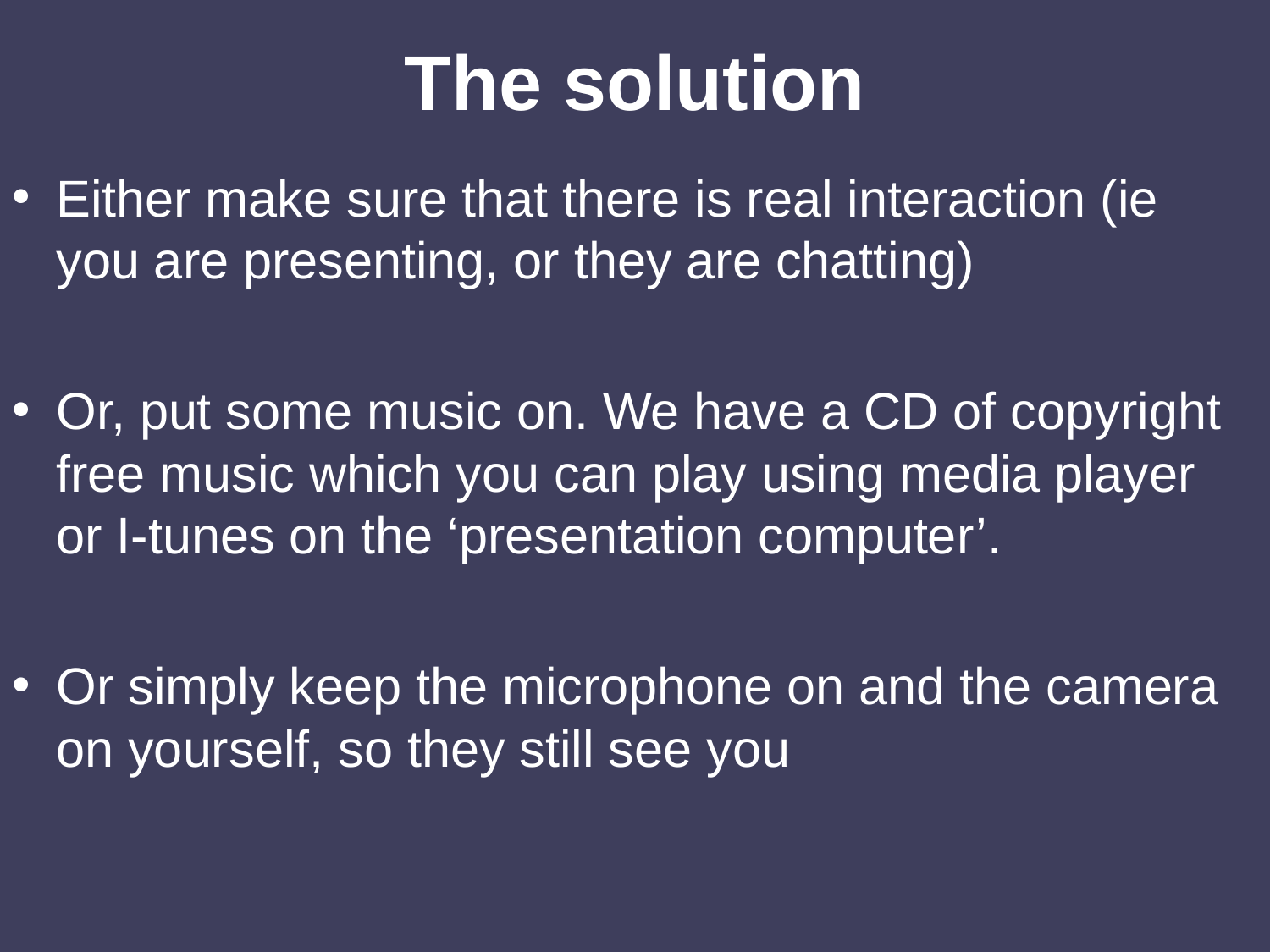

# The solution
Either make sure that there is real interaction (ie you are presenting, or they are chatting)
Or, put some music on. We have a CD of copyright free music which you can play using media player or I-tunes on the ‘presentation computer’.
Or simply keep the microphone on and the camera on yourself, so they still see you

## Slide 47
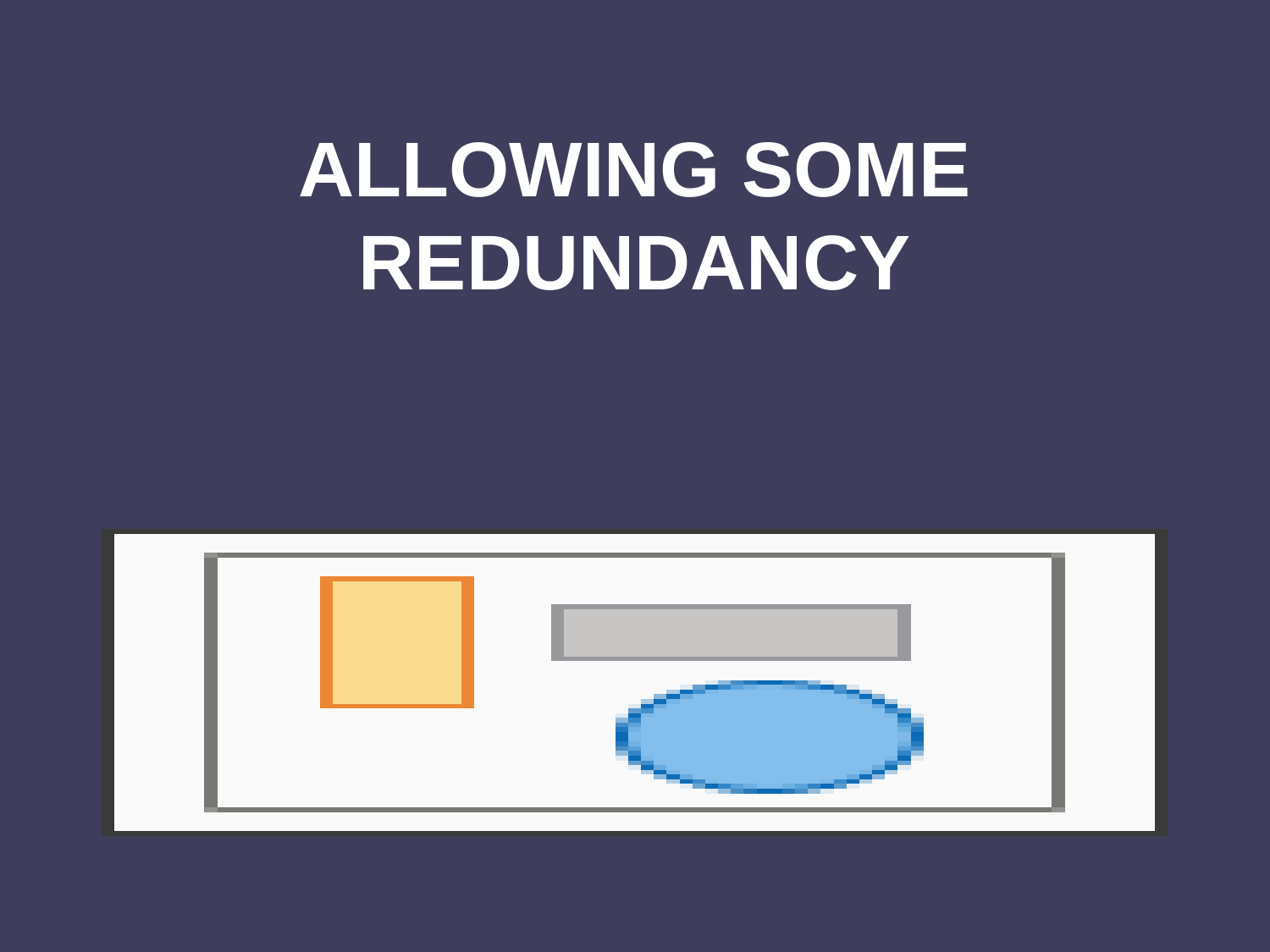

# ALLOWING SOME REDUNDANCY

## Slide 48
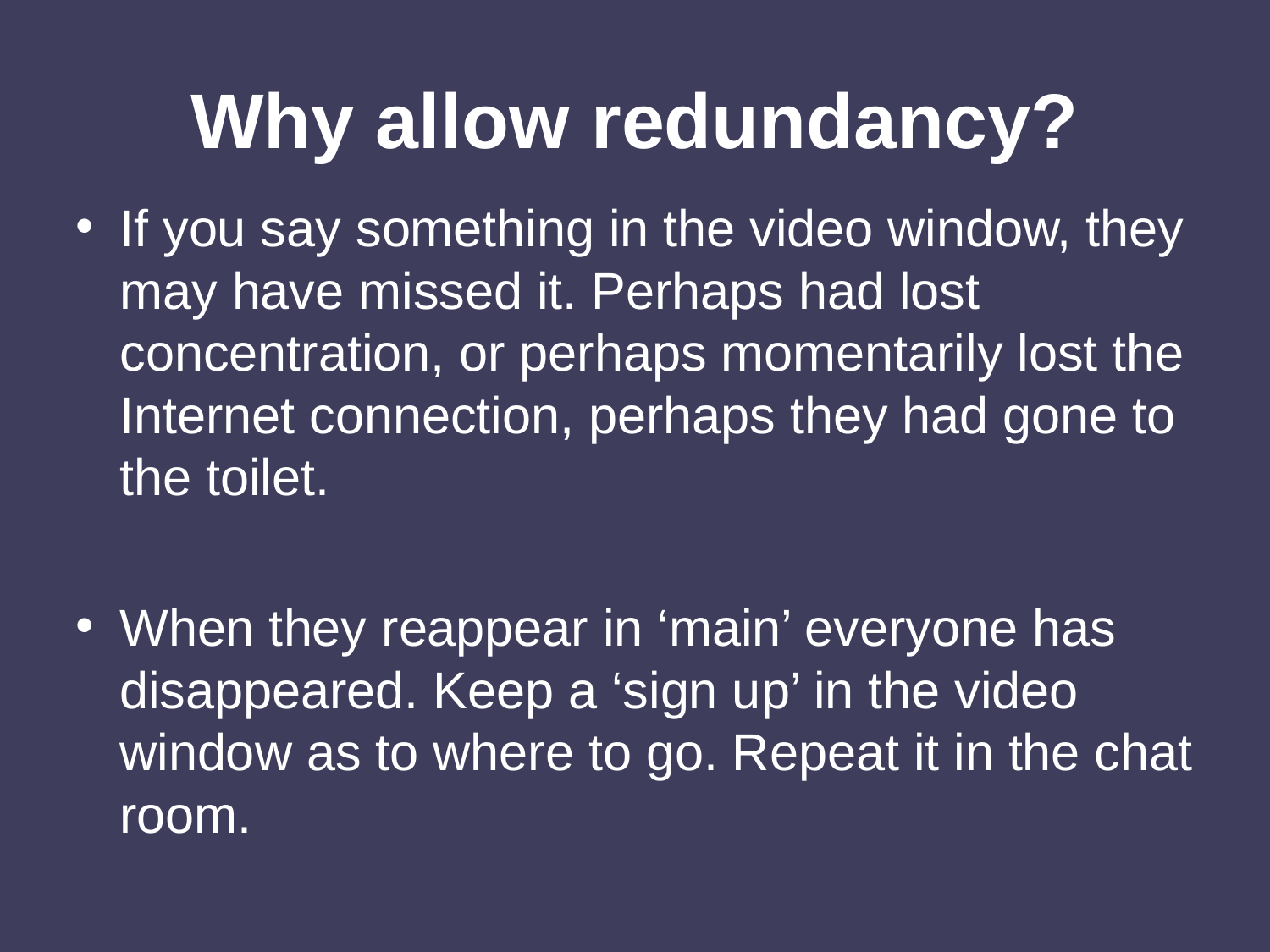

# Why allow redundancy?
If you say something in the video window, they may have missed it. Perhaps had lost concentration, or perhaps momentarily lost the Internet connection, perhaps they had gone to the toilet.
When they reappear in ‘main’ everyone has disappeared. Keep a ‘sign up’ in the video window as to where to go. Repeat it in the chat room.

## Slide 49
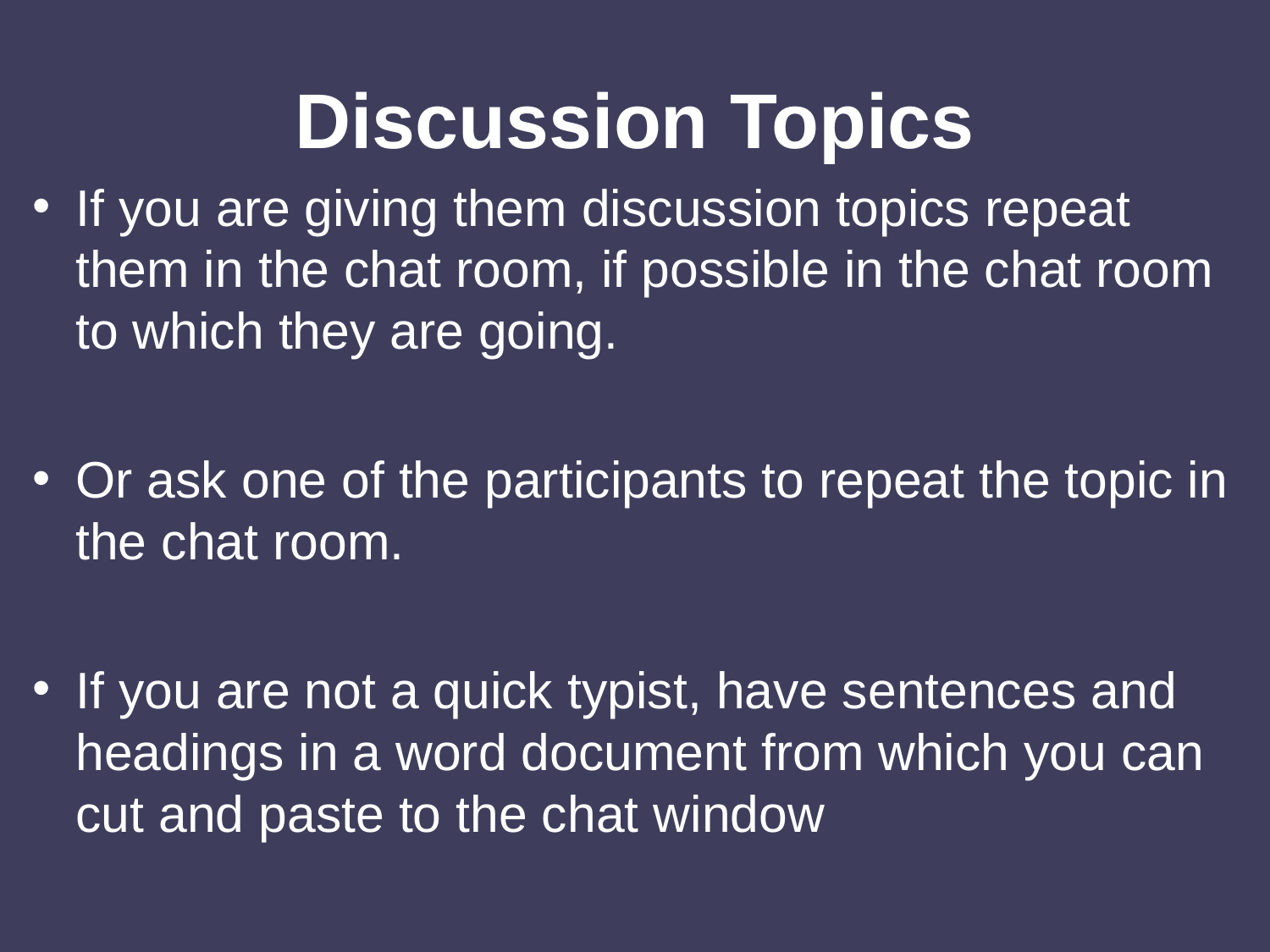

# Discussion Topics
If you are giving them discussion topics repeat them in the chat room, if possible in the chat room to which they are going.
Or ask one of the participants to repeat the topic in the chat room.
If you are not a quick typist, have sentences and headings in a word document from which you can cut and paste to the chat window

## Slide 50
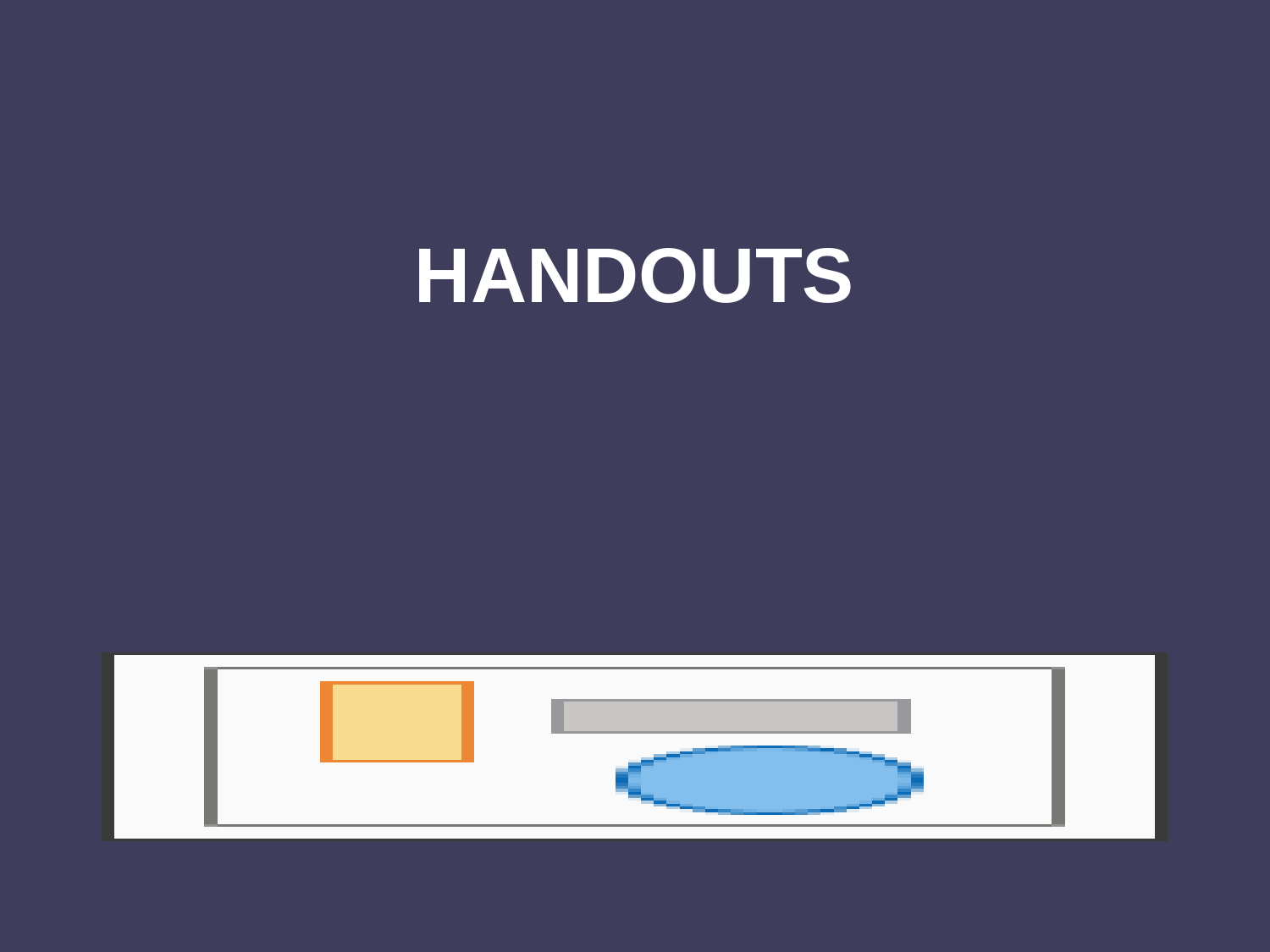

# HANDOUTS

## Slide 51
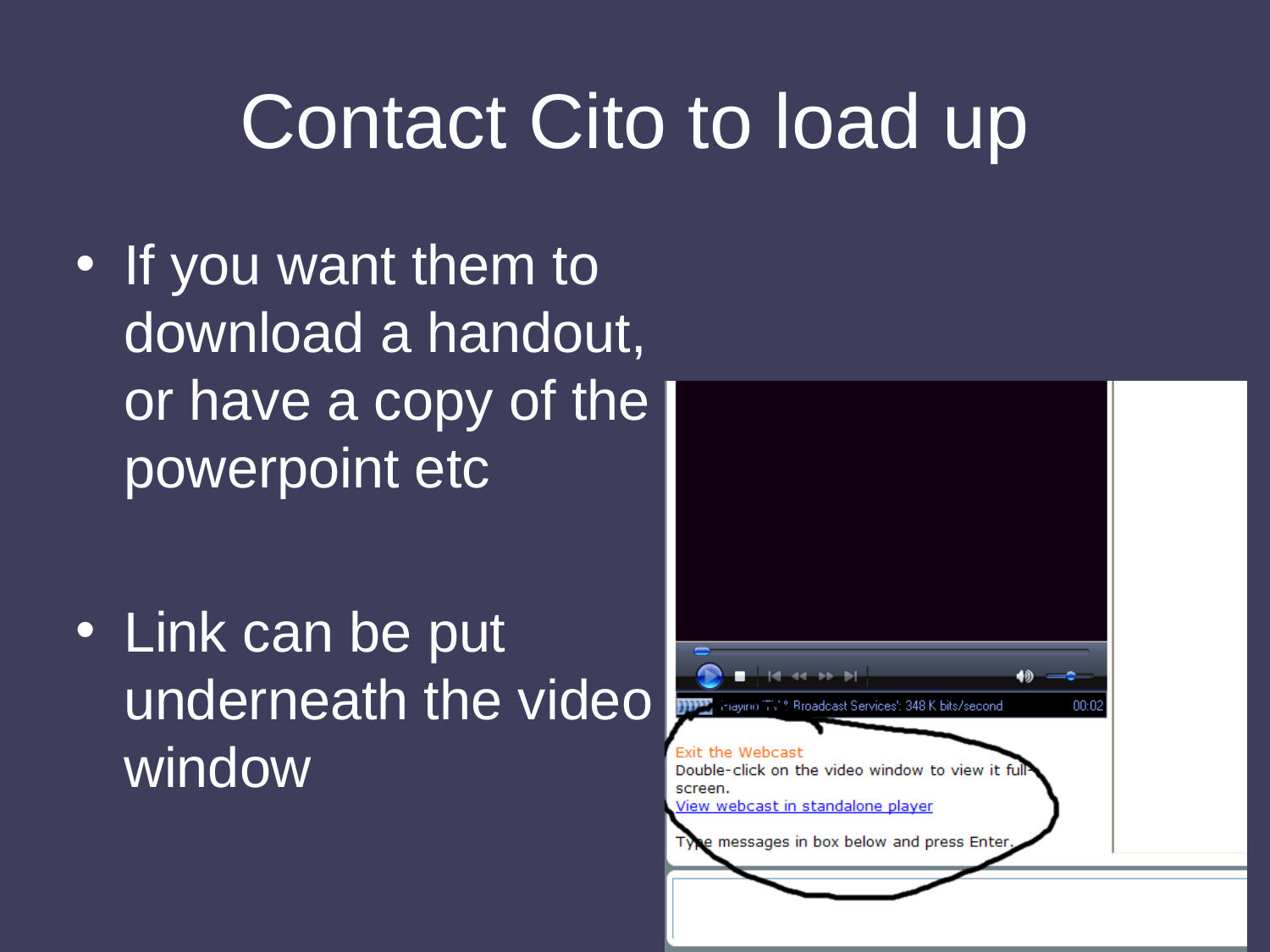

# Contact Cito to load up
If you want them to download a handout, or have a copy of the powerpoint etc
Link can be put underneath the video window

## Slide 52
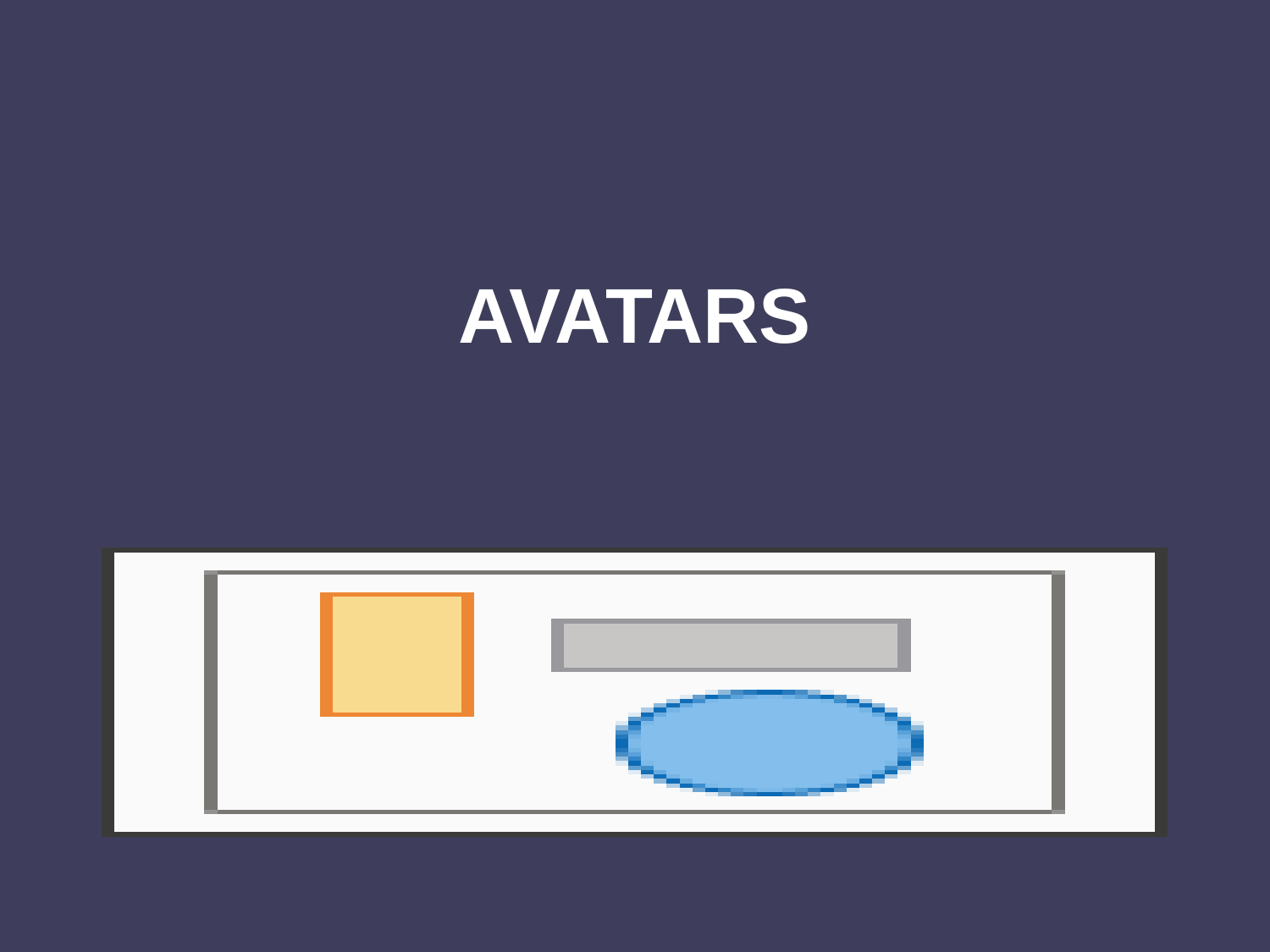

# AVATARS

## Slide 53
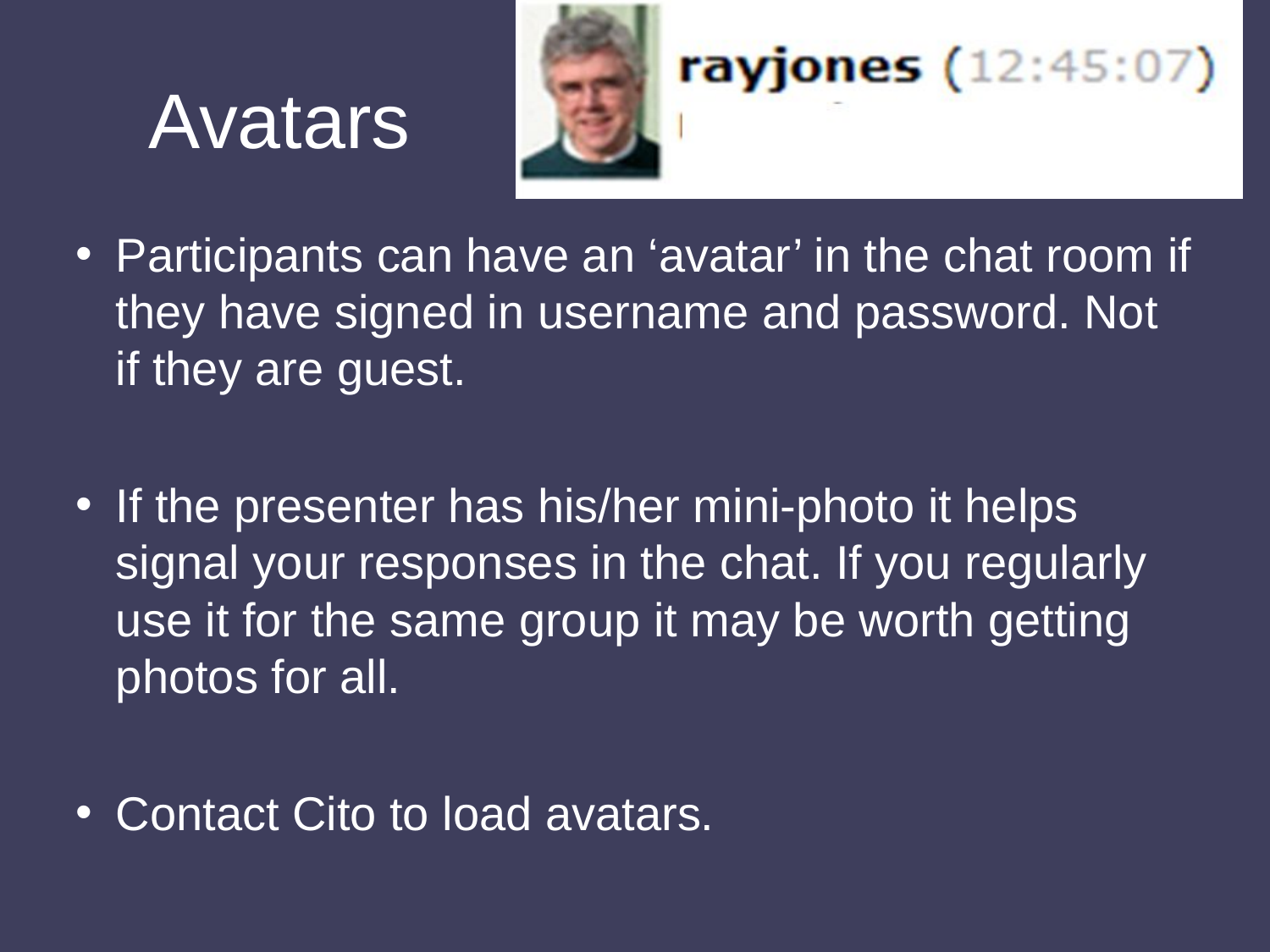

# Avatars
Participants can have an ‘avatar’ in the chat room if they have signed in username and password. Not if they are guest.
If the presenter has his/her mini-photo it helps signal your responses in the chat. If you regularly use it for the same group it may be worth getting photos for all.
Contact Cito to load avatars.

## Slide 54
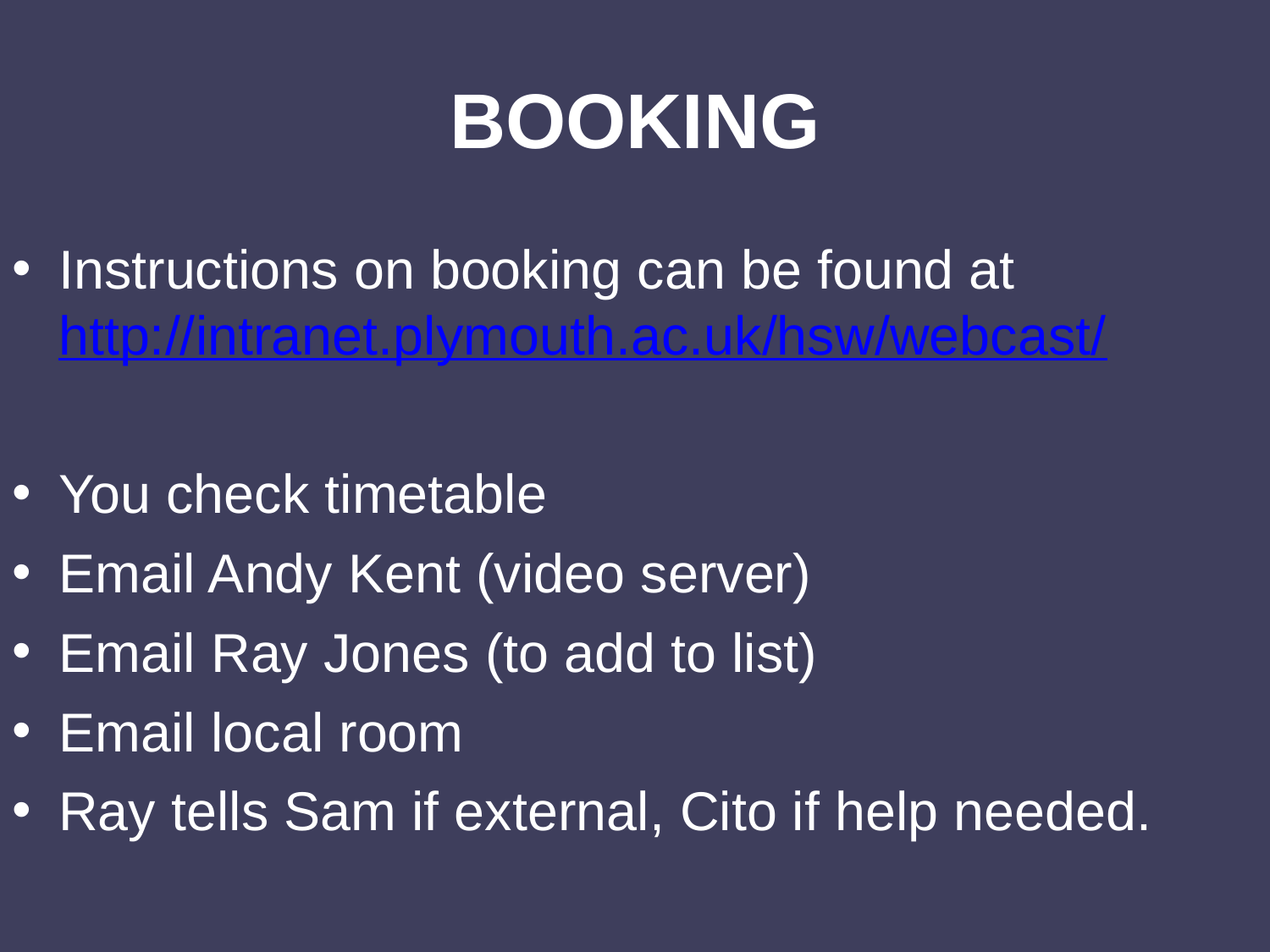

# BOOKING
Instructions on booking can be found at http://intranet.plymouth.ac.uk/hsw/webcast/
You check timetable
Email Andy Kent (video server)
Email Ray Jones (to add to list)
Email local room
Ray tells Sam if external, Cito if help needed.

## Slide 55
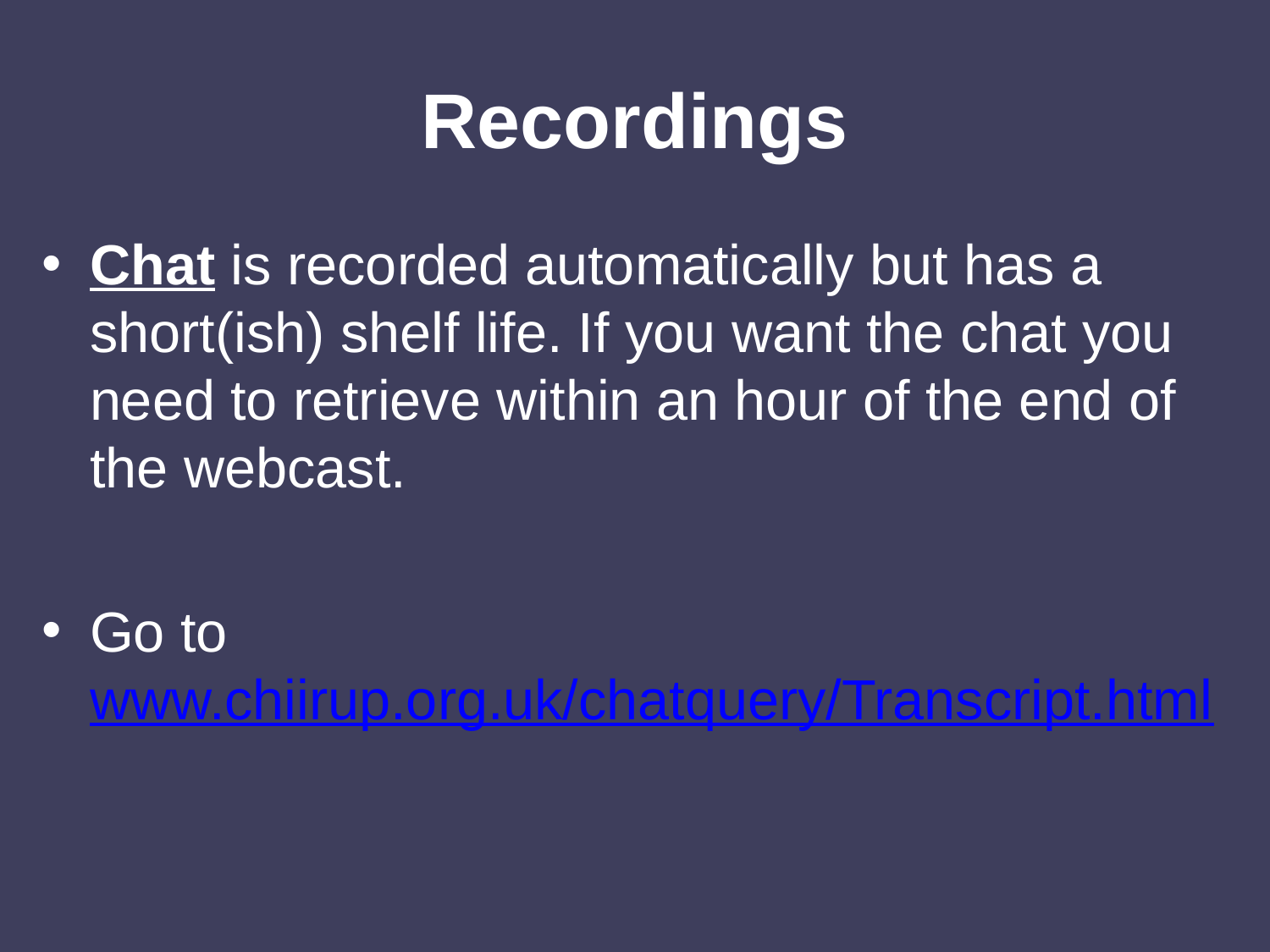

# Recordings
Chat is recorded automatically but has a short(ish) shelf life. If you want the chat you need to retrieve within an hour of the end of the webcast.
Go to www.chiirup.org.uk/chatquery/Transcript.html

## Slide 56
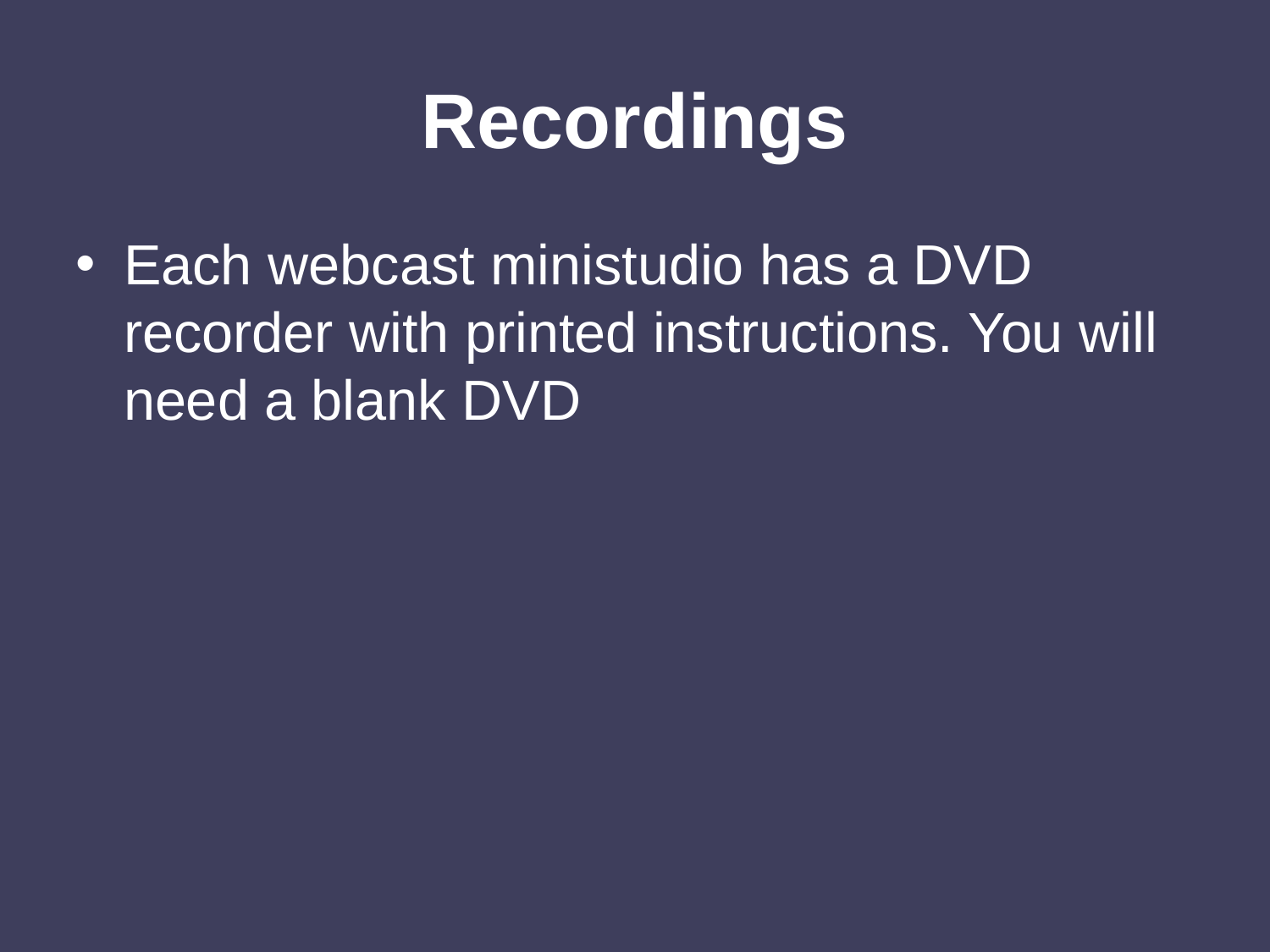

# Recordings
Each webcast ministudio has a DVD recorder with printed instructions. You will need a blank DVD

## Slide 57
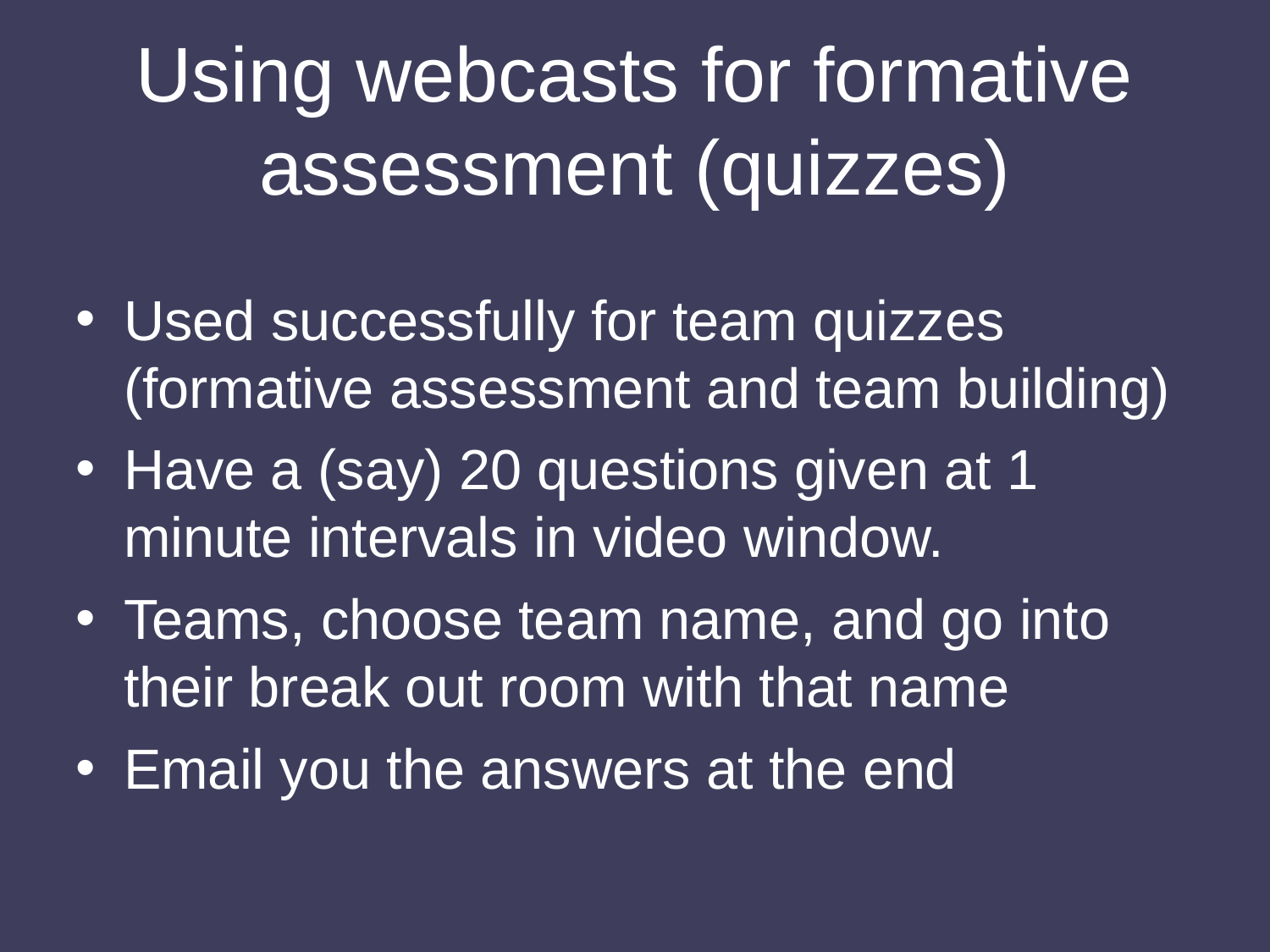

# Using webcasts for formative assessment (quizzes)
Used successfully for team quizzes (formative assessment and team building)
Have a (say) 20 questions given at 1 minute intervals in video window.
Teams, choose team name, and go into their break out room with that name
Email you the answers at the end
